# The MEKK1 PHD ubiquitinates TAB1 to activate MAPKs in response to cytokines

**DOI:** 10.15252/embj.201488351

**Published:** 2014-09-26

**Authors:** Nikolaos Charlaftis, Tesha Suddason, Xuefeng Wu, Saba Anwar, Michael Karin, Ewen Gallagher

**Affiliations:** 1 Department of Medicine, Imperial College LondonLondon, UK; 2 Laboratory of Gene Regulation and Signal Transduction, Departments of Pharmacology and Pathology, University of California San Diego, School of MedicineSan Diego, CA, USA

**Keywords:** differentiation, signalling, stem cell, tumourigenesis, ubiquitin

## Abstract

Unlike the other MAP3Ks, MEKK1 (encoded by *Map3k1*) contains a PHD motif. To understand the role of this motif, we have created a knockin mutant of mouse *Map3k1* (*Map3k1*^*m*^^*PHD*^) with an inactive PHD motif. *Map3k1*^*m*^^*PHD*^ ES cells demonstrate that the MEKK1 PHD controls p38 and JNK activation during TGF-β, EGF and microtubule disruption signalling, but does not affect MAPK responses to hyperosmotic stress. Protein microarray profiling identified the adaptor TAB1 as a PHD substrate, and TGF-β- or EGF-stimulated *Map3k1*^*m*^^*PHD*^ ES cells exhibit defective non-canonical ubiquitination of MEKK1 and TAB1. The MEKK1 PHD binds and mediates the transfer of Lys63-linked poly-Ub, using the conjugating enzyme UBE2N, onto TAB1 to regulate TAK1 and MAPK activation by TGF-β and EGF. Both the MEKK1 PHD and TAB1 are critical for ES-cell differentiation and tumourigenesis. *Map3k1*^*m*^^*PHD*^^/+^ mice exhibit aberrant cardiac tissue, B-cell development, testis and T-cell signalling.

## Introduction

Mitogen-activated protein kinases (MAPKs) are involved in numerous cellular processes including cell death, proliferation, embryonic stem (ES) cell differentiation, migration and lymphocyte development (Chen *et al*, 2001; Xia & Karin, 2004; Karin & Gallagher, 2005; Raman *et al*, 2007). MEK Kinase 1 (MEKK1) is a member of the MAPK Kinase (MAP2K) Kinase (MAP3K) family that can regulate c-Jun N-terminal kinase (JNK) and p38 by phosphorylation of their upstream MAP2Ks (MAP2K4 and MAP2K7) activation loop (Weston & Davis, 2002; Karin & Gallagher, 2005). *Map3k1*^*ΔKD*^ mice, lacking the MEKK1 kinase domain, have demonstrated the importance of MEKK1 signalling in B-cell germinal centre formation, cell cycle progression, antibody production, tumour necrosis factor (TNF) receptor (TNFR)-dependent JNK activation and keratinocyte migration (Xia *et al*, 2000; Zhang *et al*, 2003; Gallagher *et al*, 2007). CD4^+^ T cells from *Map3k1*^*ΔKD*^ mice display an elevated production of T helper (Th) 2 cell cytokines, and mechanistically MEKK1 triggers JNK1-dependent negative regulation of the Homologous to the E6-AP Carboxyl Terminus (HECT) E3 Ubiquitin (Ub) ligase Itch (Gao *et al*, 2004; Gallagher *et al*, 2006). Ablation of the *Map3k1* in ES cells has revealed a role for MEKK1 in MAPK activation by epidermal growth factor (EGF), LPA, cold shock, microtubule disruption and hyperosmotic stress (Gibson *et al*, 1999; Yujiri *et al*, 1999).

Lys48-linked Ub chains can modify protein targets for degradation by the 26S proteasome, while non-canonical Ub conjugation controls protein–protein interactions and modifies the biochemical activity of the target protein (Hochstrasser, 1996; Kravtsova-Ivantsiv & Ciechanover, 2012). Recent research has demonstrated that both Lys63-linked and linear poly-Ub chains are critical for the control of nuclear factor κ-light-chain-enhancer of activated B cells (NF-κB) and MAPK activation in cells (Matsuzawa *et al*, 2008; Karin & Gallagher, 2009; Ikeda *et al*, 2010; Walczak *et al*, 2012). Conjugation of Ub to target proteins requires the concentrated activities of a Ub-activating enzyme (E1), a Ub-conjugating enzyme (E2) and an E3 Ub ligase (Gao & Karin, 2005; Kravtsova-Ivantsiv & Ciechanover, 2012).

In addition to functioning as a protein kinase, MEKK1, uniquely among the MAP3Ks, exhibits E3 Ub ligase activity (Lu *et al*, 2002; Witowsky & Johnson, 2003). This is achieved by the plant homeodomain (PHD), present within the MEKK1 amino-terminal regulatory region, that closely resembles a really interesting new gene (RING) finger (Lu & Hunter, 2009). Overexpression of MEKK1 in cell lines was shown to negatively regulate ERK expression in response to osmotic stress and c-Jun following EGF receptor (EGFR) signalling (Lu *et al*, 2002; Xia *et al*, 2007). In addition, MEKK1 also undergoes PHD-dependent auto-ubiquitination (Lu *et al*, 2002; Witowsky & Johnson, 2003; Gallagher *et al*, 2007). Following T-cell receptor (TCR) engagement, MEKK1 is modified by Lys63-linked poly-Ub and this correlates with p38 and JNK activation (Matsuzawa *et al*, 2008; Wang *et al*, 2008; Enzler *et al*, 2009). In addition, full-length MEKK1 may also be targeted and cleaved by caspases in response to some forms of cellular stress (Cardone *et al*, 1997).

Transforming growth factor-β (TGF-β) activated kinase 1 (TAK1, encoded by *Map3k7*) was identified as a MAP3K that becomes activated following a variety of mitogenic stimuli, including TGF-β and bone morphogenetic protein (Yamaguchi *et al*, 1995). TAK1 interacts with and is activated by TAK1-binding proteins (TABs) (Shibuya *et al*, 1996; Takaesu *et al*, 2000). TAB1 is distinct among the TABs in containing a protein phosphatase 2C (PP2C)-like region and can bind TRAF6, p38 and TAK1 (Shibuya *et al*, 1996; Ge *et al*, 2002; Kang *et al*, 2006). Along with protein–protein interactions with TABs, non-canonical ubiquitination is a critical component in TAK1 activation (Wang *et al*, 2001). TAB1 can activate TAK1 by overexpression of its C-terminal 68 residues (Shibuya *et al*, 1996; Ono *et al*, 2001). TAB2 and TAB3 can be recruited to Lys63-linked poly-Ub chains by their zinc finger (ZnF) motifs (Kanayama *et al*, 2004). Genetic analysis has demonstrated that both TAK1 and TAB1 are critical for mammalian embryogenesis (Shim *et al*, 2005). MAPK signalling from the CD40 cytokine receptor requires both MEKK1 and TAK1, though what interplay occurs between these MAP3Ks, and why MEKK1, but not TAK1, contains a PHD motif remains to be clarified (Matsuzawa *et al*, 2008).

To better assess the role of the PHD in MAPK signalling, we have utilised gene targeting to create the *Map3k1*^*mPHD*^ allele and found that an intact MEKK1 PHD is necessary for p38 and JNK activation by TGF-β and EGF in response to microtubule disruption in ES cells. We also found that the MEKK1 PHD mediates the Lys63-linked poly-ubiquitination of the adaptor TAB1 following cytokine stimulation at key lysines, thereby controlling the interaction between full-length TAB1 and TAK1, and activates TAK1, JNK and p38. The MEKK1 PHD is also required for TAB1-dependent ES-cell differentiation and tumour development in mice. *Map3k1*^*mPHD/+*^ mice have cardiac fibrosis and muscle damage, reduced B-cell development beyond the pro-B-cell stage, condensed and fewer numbers of Leydig cells and reduced Itch activation in T cells.

## Results

### Gene targeting of the PHD

**Figure 1:**
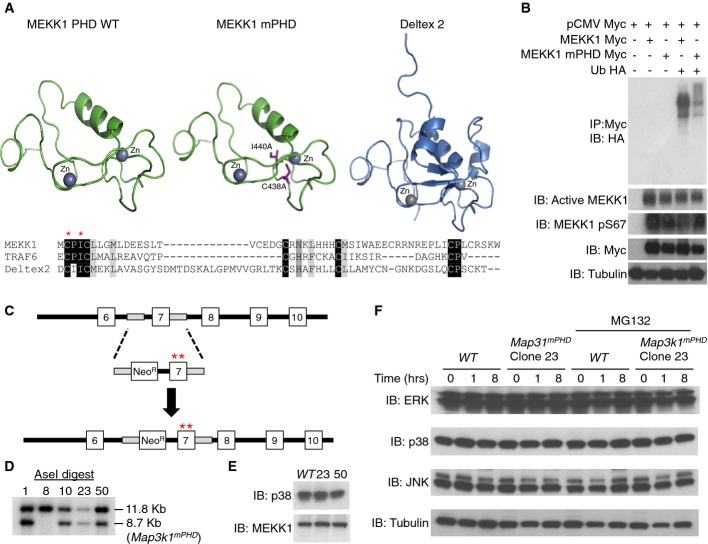
Gene targeting and analysis of the MEKK1 PHD Molecular modelling of MEKK1 PHD. The mouse MEKK1 PHD sequence (residues 437–490) was submitted to the Phyre^2^ server to produce the WT MEKK1 PHD model. The side chain of residues 438 and 440 was then manually truncated to an alanine to create the mutant MEKK1 PHD model. As a comparison the structure of the mouse Deltex 2 RING (residues 407–470) is shown. The amino acid sequence alignment compares MEKK1 PHD, TRAF6 RING and Deltex 2 RING, with the conserved residues corresponding to MEKK1 residues 438 and 440 indicated by asterisks.The MEKK1 mPHD (C438A, I440A) mutant is unable to undergo auto-ubiquitination. HEK 293 cells were transiently transfected with WT MEKK1-Myc or MEKK1 C438A, I440A-Myc and Ub-HA as indicated. After 48 h the cells were lysed and analysed by immunoblotting (IB) with anti-HA antibody. MEKK1 phosphorylation was detected with anti-active MEKK1 (phospho T1381) and anti-phospho S67 antibodies. Anti-Myc antibody was used to immunoprecipitate (IP) MEKK1 or to detect total MEKK1. Anti-tubulin antibody was used as a loading control.Strategy for generating *Map3k1*^*mPHD*^ knockin mice.Targeted ES cells were genotyped by Southern blotting to confirm the in-frame insertion of the mPHD mutation into *Map3k1* exon 7.MEKK1 expression is similar between WT and *Map3k1*^*mPHD*^ ES cell clones. ES cell clones were lysed and analysed by IB using the indicated antibodies.MAPK stability is not critically dependent on the PHD. ES cell clones were left unstimulated or stimulated for up to 8 h with 500 mM sorbitol in the presence or absence of 25 μM MG132. Lysates were analysed by IB with the indicated antibodies. Data information: Results are representative of three independent experiments. Source data are available online for this figure.

To design a loss-of-function mutation within the MEKK1 PHD, the mouse MEKK1 PHD amino acid sequence was analysed by Phyre^2^ software, and its structure was modelled upon the known structure of the Deltex 2 RING E3 Ub ligase (Fig 1A) (Kelley & Sternberg, 2009). Sequence alignment of the RING motifs from MEKK1 (residues 437–490), TRAF6 (residues 69–107) and Deltex 2 (residues 407–470) revealed conserved cysteine and isoleucine residues at positions 438 and 440, respectively, of MEKK1, within the RING structure that binds E2 Ub-conjugating enzymes (UBEs) (Yin *et al*, 2009). We hypothesised that mutation of MEKK1 residues C438 and I440 into alanine residues (the MEKK1 mPHD mutant) would disrupt the function of the PHD as an E3 Ub ligase.

To test whether the MEKK1 mPHD retains E3 Ub ligase function, we transfected HEK 293 cells with full-length MEKK1 PHD and mPHD. The experiment demonstrated that MEKK1 auto-ubiquitination by the PHD was significantly reduced relative to the WT motif (Fig 1B). Relative MEKK1 phosphorylation at residues S67 and T1381 (which both reflect MEKK1 activation) was similar between MEKK1 WT and mPHD, indicating that the PHD motif is not involved in the mechanism of overexpressed MEKK1 activation and auto-phosphorylation, as previously reported (Supplementary Fig S1A) (Gallagher *et al*, 2002; Lu *et al*, 2002; Matsuzawa *et al*, 2008). To understand the mechanism by which MEKK1 acts as an E3 Ub ligase, we performed *in vitro* ubiquitination assays testing E2 conjugating enzymes that can act in concert with UBE1 and the MEKK1 PHD (Supplementary Fig S1B). The MEKK1 PHD underwent strong auto-ubiquitination in the presence of UBE2D2, UBE2D3 or UBE2N:UBE2V1 (Supplementary Fig S1B) and predominantly formed Lys63-linked poly-Ub. Conversely, a relatively small amount of linear Ub chains were generated in ubiquitination assays with the MEKK1 PHD (Supplementary Fig S1C). Analysis of deubiquitinating enzymes (DUBs) (Reyes-Turcu *et al*, 2009) that can act as deubiquitinating peptidases for auto-ubiquitinated MEKK1 identified Ub-specific proteases (USPs) 2, 7 and 8 (Supplementary Fig S1D). Yeast two-hybrid analysis demonstrated that residues 1–719 of the MEKK1 amino-terminal regulatory domain bind to UBE2N and that this interaction is abolished by the mPHD mutation, which is located within this fragment (Supplementary Fig S1E).

To examine the physiological consequences of the MEKK1 mPHD mutation in mammalian biology, we generated *Map3k1*^*mPHD*^ knockin mice, utilising a targeting vector containing a *loxP*-flanked neomycin resistance cassette and a mutated *Map3k1* exon 7 to insert the mPHD mutation into the *Map3k1* locus on chromosome 13 (Fig 1C). *Map3k1*^*mPHD*^ ES cell clones were genotyped by Southern blotting and genomic PCR for the in-frame insertion of the mPHD mutation into mouse chromosome 13 (Fig 1D). *Map3k1*^*mPHD*^ ES cells were found to express full-length MEKK1 at the same amount as the WT protein (Fig 1E).

It was reported that overexpression of the MEKK1 PHD motif can negatively regulate ERK2 expression following hyperosmotic stress (Lu *et al*, 2002). To test MEKK1-dependent negative regulation of ERK2, we treated *Map3k1*^*mPHD*^ ES cells with sorbitol over 8 h (Fig 1F). ERK stability was unaltered in *Map3k1*^*mPHD*^ relative to WT ES cells (Fig 1F), and the protein stability of p38 and JNK was also unchanged in *Map3k1*^*mPHD*^ ES cells (Fig 1F).

### Impaired TGF-β, EGF and nocodazole-induced JNK and p38 activation in *Map3k1*^*mPHD*^ ES cells

**Figure 2:**
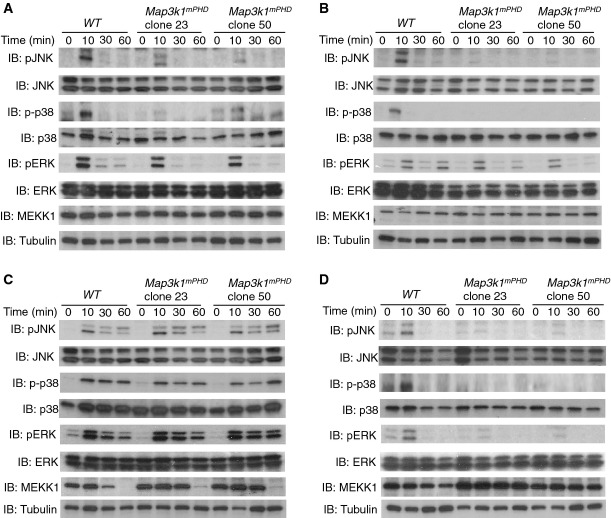
*Map3k1*^*mPHD*^ ES cells exhibit defective JNK and p38 activation following TGF-β, EGF and nocodazole stimulation A–D WT and *Map3k1*^*mPHD*^ ES cells were kept on low serum and stimulated with (A) TGF-β (10 ng/ml), (B) EGF (100 ng/ml), (C) sorbitol (500 mM) or (D) nocodazole (0.5 μg/ml) for 10, 30 and 60 min or left unstimulated. Cells were lysed and analysed by IB using the indicated antibodies. Data information: Results are representative of three independent experiments. Source data are available online for this figure.

We next tested whether MAPK activation was altered between *Map3k1*^*mPHD*^ and WT ES cells stimulated with TGF-β, EGF, hyperosmotic stress and the microtubule-disrupting agent nocodazole (Fig 2A–D). Microarray profiling of pluripotent *Map3k1*^*mPHD*^ and WT ES cells revealed normal expression of TGF-β receptors (TGFβRs) (Supplementary Fig S7A and B and Supplementary Table S1). *Map3k1*^*mPHD*^ ES cells exhibited impaired JNK and p38, but unaltered ERK, activation after TGF-β stimulation (Fig 2A). SMAD expression and phosphorylation, however, were unaffected in *Map3k1*^*mPHD*^ relative to WT ES cells (Supplementary Fig S2A). Similarly, microarray profiling revealed normal expression of EGF family receptors (EGFRs) (Supplementary Table S1), but *Map3k1*^*mPHD*^ ES cells have significantly impaired JNK and p38 activation after EGF stimulation (Fig 2B). ERK, JNK and p38 activation were all unaltered in *Map3k1*^*mPHD*^ ES cells treated with sorbitol to induce hyperosmotic stress (Fig 2C). We did note, however, that full-length MEKK1 protein expression was unstable following treatment with sorbitol, with its expression significantly reduced after 30 min, and after several hours, MEKK1 expression was no longer detected (Supplementary Fig S2B). Although the kinetics of MEKK1 degradation were altered in *Map3k1*^*mPHD*^ relative to WT cells, mutation of the MEKK1 PHD was insufficient to prevent its degradation after a few hours (Supplementary Fig S2B). *Map3k1*^*mPHD*^ ES cells show impaired ERK, JNK and p38 activation after incubation with nocodazole (Fig 2D). From these experiments, we can propose a new model whereby the MEKK1 PHD controls downstream MAPK activation following cytokine stimulation and microtubule disruption, but is not critical for MAPK activation in response to hyperosmotic stress (Supplementary Fig S2C).

### Identification of novel PHD substrates

**Figure 3:**
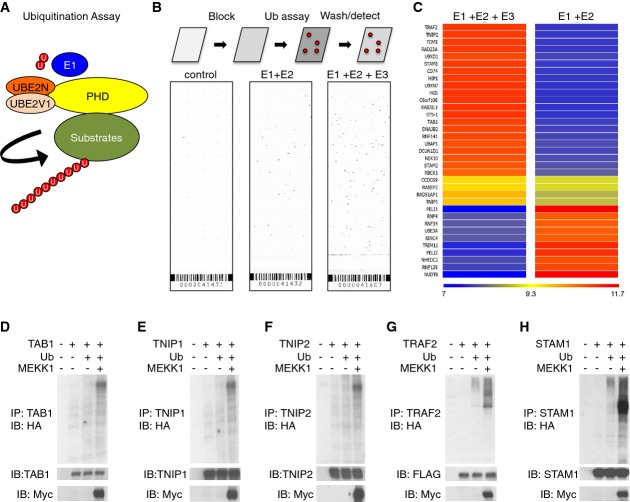
Protein array screening for MEKK1 PHD substrates A Schematic illustrating the protein array screen for MEKK1 PHD substrates using UBE1, UBE2N:UBE2V1 and MEKK1 PHD in a ubiquitination assay. B Ubiquitination assays were performed comprising UBE1, UBE2N:UBE2V1 and MEKK1 PHD or UBE1 and UBE2N:UBE2V1 in the presence of biotin-Ub and profiled using Protoarray Human Protein Microarrays v.5. C A heat map comparing hits between UBE1 +  UBE2N:UBE2V1 and UBE1 +  UBE2N:UBE2V1 +  MEKK1 PHD reactions. D–H Ubiquitination of (D) TAB1, (E) TNIP1, (F) TNIP2, (G) TRAF2, and (H) STAM1 by MEKK1. HEK 293 cells were transfected as indicated, lysates prepared and analysed by IP and IB with the indicated antibodies. Data information: Results are representative of three independent experiments. Source data are available online for this figure.

Since the defects present in *Map3k1*^*mPHD*^ ES cells demonstrate that the MEKK1 PHD is not essential for MAPK stability, but is important for JNK and p38 activation, we used protein microarray profiling of 9,400 full-length human proteins in ubiquitination reactions to identify new MEKK1 PHD substrates (Fig 3A and B). Bioinformatics analysis of the protein array data revealed 55 proteins as potential substrates for a ubiquitination reaction comprising UBE2N:UBE2V1 (Supplementary Table S2), and 82 proteins as potential substrates for a ubiquitination reaction containing UBE2N:UBE2V1 and the MEKK1 PHD (Supplementary Table S3). To compare the results from the two array screens, a heat map of the data sets was created using GeneSpring software (Fig 3C).

Pathway analysis of the hits from the protein array screen identified TAB1 as a critical component of the TGF-β signal transduction pathway (Supplementary Fig S3A), where we identified defective MAPK signalling in *Map3k1*^*mPHD*^ ES cells (Fig 2A). In addition to TAB1, a number of the protein array hits, including TNF receptor-associated factor 2 (TRAF2), TNFAIP3 interacting protein 1 (TNIP1), TNFAIP3 interacting protein 2 (TNIP2) and Signal-Transducing Adaptor Molecule 1 (STAM1), are also classified as signal transduction adaptors and were selected along with TAB1 as possible PHD substrates that might mediate MEKK1 PHD-dependent TGF-β or EGF MAPK activation in ES cells, and to validate our Ub substrate screening in orthogonal assays.

Overexpressed TAB1, TRAF2, TNIP1, TNIP2 and STAM1 proteins were then purified from HEK 293 cells and examined in ubiquitination assays with UBE1, UBE2N:UBE2V1 and MEKK1 PHD or MEKK1 mPHD. In all cases, enhanced poly-Ub modification was detected when the MEKK1 PHD was used as an E3 Ub ligase, whereas the MEKK1 mPHD mutant was unable to enhance the poly-Ub of any of the proteins tested, confirming the protein array screening by an orthogonal assay approach (Supplementary Fig S3B–F). We then examined whether MEKK1 acts as an E3 Ub ligase towards these proteins in cells by cotransfection of TAB1, TNIP1, TNIP2, TRAF2 and STAM1 with MEKK1 and HA-Ub into HEK 293 cells. Immunoprecipitation and Western analysis indicated that poly-Ub of all five proteins was strongly enhanced by coexpression with MEKK1 (Fig 3D–H).

### PHD-dependent TAB1 Lys63-linked Ub is critical for EGF and TGF-β signalling

**Figure 4:**
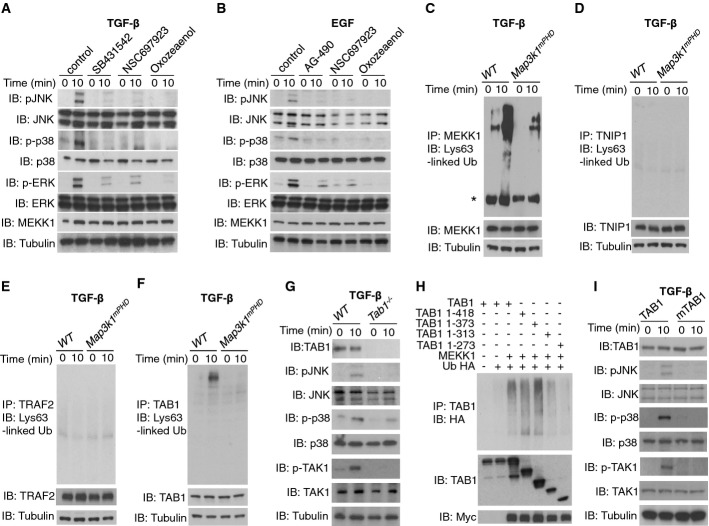
MEKK1 PHD dependence of TGF-β-stimulated TAK1 and MAPK signalling A WT ES cells were rested in low serum conditions and stimulated for 10 min with TGF-β (10 ng/ml) in the presence or absence of DMSO (control), SB431542, NSC697923, (5Z)-7-Oxozeaenol (Oxozeaenol) or left unstimulated. Lysates were made and analysed by IB using the indicated antibodies. B WT ES cells were rested in low serum and stimulated for 10 min with EGF in the presence or absence of DMSO (control), AG-490, NSC697923, (5Z)-7-Oxozeaenol (Oxozeaenol) or left unstimulated. Lysates were prepared and analysed as above. C–F WT or *Map3k1*^*mPHD*^ ES cells kept in low serum were stimulated or not for 10 min with TGF-β (10 ng/ml). Lysates were prepared and IP and IB performed using the indicated antibodies (* indicates a non-specific band). G WT or *Tab1*^*−/−*^ ES cells were analysed as above by the indicated antibodies before and after TGF-β (10 ng/ml) stimulation. H HEK 293 cells were transfected with the indicated constructs and lysates made. IP and IB were performed using the indicated antibodies. I *Tab1*^*−/−*^ ES cells were transfected with TAB1 or mTAB1 as indicated, rested in low serum and stimulated or not for 10 min with TGF-β (10 ng/ml). Lysates were made and analysed as above. Data information: Results are representative of three experiments. Source data are available online for this figure.

We next analysed the MEKK1 PHD as an E3 Ub ligase in EGF and TGF-β signalling. Pre-treatment of TGF-β-stimulated ES cells with small molecule inhibitors of TGFβR, UBE2N or TAK1 inhibited JNK and p38 activation (Fig 4A). Similarly, pre-treatment of EGF-stimulated ES cells with inhibitors of EGFR, UBE2N or TAK1 inhibited EGF-mediated JNK and p38 activation (Fig 4B). Next, WT and *Map3k1*^*mPHD*^ ES cells were stimulated with TGF-β and endogenous MEKK1, TRAF2, TAB1, TNIP1, TNIP2 and STAM1 were immunoprecipitated and then immunoblotted with a Lys63-linked Ub antibody. While we detected Lys63-linked Ub upon MEKK1 and TAB1 in TGF-β stimulated WT ES cells, no Lys63-linked Ub was detected upon TRAF2, TNIP1, TNIP2 and STAM1 (Fig 4C–F and unpublished observations). The Lys63-linked Ub of MEKK1 and TAB1 was strongly reduced in *Map3k1*^*mPHD*^ ES cells (Fig 4C and F). An intact MEKK1 PHD is critical for the Lys63-linked ubiquitination of TAB1 (Supplementary Fig S4A), and MEKK1 and TAB1 coimmunoprecipitated when coexpressed in HEK 293 cells (Supplementary Fig S4B).

To understand the role TAB1 plays in EGF and TGF-β signalling in ES cells, we generated *Tab1*^*−/−*^ ES cells, utilising a targeting vector containing a *loxP*-flanked neomycin resistance cassette and a mutated *Tab1* exon 1 to insert a stop codon and disrupt the *Tab1* coding sequence on chromosome 15 (Supplementary Fig S4C and D). *Tab1*^*−/−*^ ES cells displayed defective TAK1 and MAPK activation following TGF-β or EGF stimulation (Fig 4G and Supplementary Fig S4E), indicating for the first time that TAB1 is critical for EGF signalling. To map MEKK1-dependent ubiquitination sites within TAB1, we utilised a sequential series of TAB1 deletion mutants and found that TAB1 residues 1–373 were strongly ubiquitinated, whereas 1–313 were weakly ubiquitinated and 1–273 were barely ubiquitinated (Fig 4H and Supplementary Fig S4F). Subsequent mutation of lysines within TAB1 residues 273–373, which contain part of the PP2C-like region of TAB1, at residues K294A, K319A, K335A and K350A blocked the MEKK1-mediated ubiquitination of TAB1, as well as the binding of TAB1 to TAK1 (Supplementary Fig S4G and H). Add-back of WT, but not mTAB1 (K294A, K319A, K335A and K350A), into *Tab1*^*−/−*^ ES cells restored TAK1 and MAPK activation by TGF-β (Fig 4I). TAB1 immunoprecipitates with endogenous MEKK1 in ES cells stimulated by TGF-β (Supplementary Fig S4I). To determine whether endogenous TAK1 is activated in a manner dependent upon the MEKK1 PHD, *Map3k1*^*mPHD*^ ES cells were stimulated with TGF-β and showed defective TAK1 phosphorylation relative to WT cells (Supplementary Fig S4J).

### The MEKK1:TAB1 signalling complex recruits TAB2 by its ZnF motif

**Figure 5:**
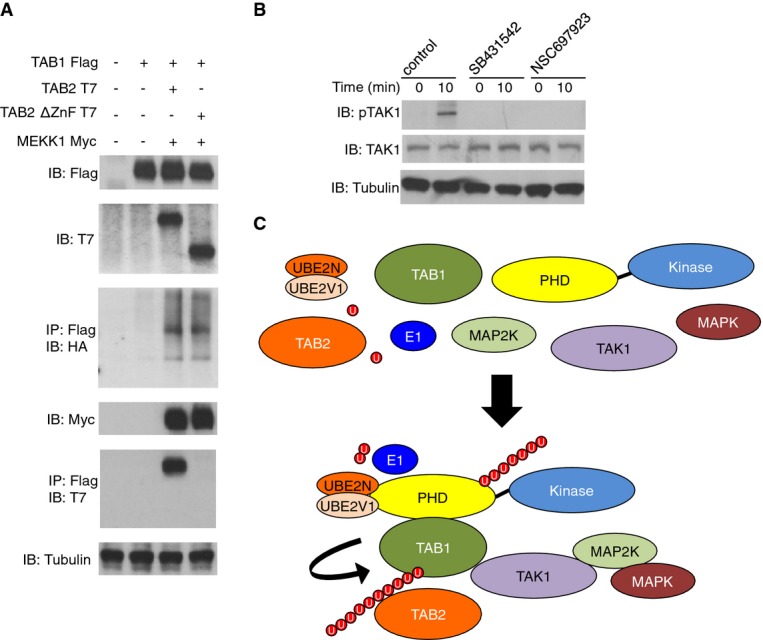
TAB2 interacts with TAB1 ubiquitinated by the MEKK1 PHD motif HEK 293 cells were transiently transfected as indicated along with HA-Ub. 48 h later cells were lysed and analysed by IP and IB using the indicated antibodies.WT ES cells were rested in low serum conditions and stimulated for 10 min with TGF-β (10 ng/ml) in the presence or absence of DMSO (control), SB431542, NSC697923 or left unstimulated. Lysates were made and analysed by IB using the indicated antibodies.Schematic diagram showing the formation of the MEKK1, TAK1 and TABs signalling complex. Source data are available online for this figure.

Although not a MEKK1 PHD motif substrate (Fig 3), TAB2 can bind poly-Ub via its ZnF motif (Kanayama *et al*, 2004). Thus, we tested whether TAB2 binds TAB1 coexpressed with MEKK1 by its ZnF motif. TAB2, but not a TAB2 mutant lacking the ZnF motif, purified with TAB1 coexpressed with, and also ubiquitinated by, MEKK1 (Fig 5A). TAK1 activation by TGF-β is critically regulated by UBE2N and TGFβR activity (Fig 5B). To identify TAK1 activation *in vitro* by the MEKK1 PHD:TAB1 complex, we utilised a two-step approach comprising a ubiquitination assay followed by a kinase assay (Supplementary Fig S5A). The MEKK1 PHD, but not MEKK1 mPHD, was able to ubiquitinate TAB1 and Ub-modified TAB1 potentiated TAK1 activation (Supplementary Fig S5B). TAB1, but not mTAB1, was ubiquitinated by MEKK1 and enhanced TAK1 activation by TAB1 (Supplementary Fig S5C). The MEKK1 PHD ubiquitinates TAB1 with Lys63-linked Ub to potentiate TAK1 activation, and TAB2 can be recruited to this signalling complex in a manner dependent upon its ZnF motif (Fig 5C).

### Pluripotent *Map3k1*^*mPHD*^ ES cells exhibit a defective gene expression signature

**Figure 6:**
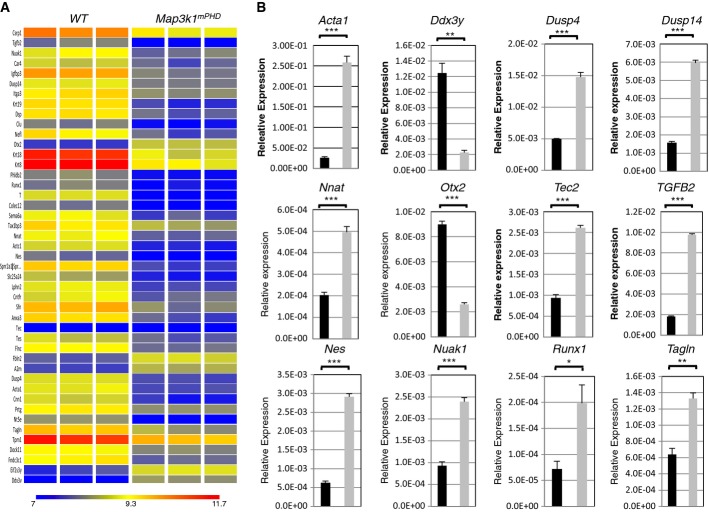
Altered gene expression in *Map3k1*^*mPHD*^ ES cells Heat map showing differential gene expression between pluripotent WT and *Map3k1*^*mPHD*^ ES cells.Confirmation of selected microarray hits by real-time PCR. (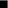) *WT* and (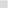) *Map3k1*^*mPHD*^ ES cells RNAs were analysed by real-time PCR. The average relative expression (± SEM) of the indicated mRNA from three independent experiments was statistically analysed, where appropriate, by two-tailed Student's *t*-test (*P ≤ 0.05; **P ≤ 0.01; ***P ≤ 0.001). Source data are available online for this figure.

To identify intrinsic gene expression defects within pluripotent *Map3k1*^*mPHD*^ ES cells, WT and *Map3k1*^*mPHD*^ ES cell cDNA were analysed using GeneChip Mouse Gene 1.0 ST arrays. A heat map of the gene expression profiles was generated with GeneSpring software (Fig 6A). 56 genes were found to be downregulated, and 12 genes were upregulated more than twofold in *Map3k1*^*mPHD*^ cells compared to WT ES cells grown in the presence of serum and LIF (Supplementary Fig S6A and Supplementary Table S1). A few of these genes, namely *Acta1*, *Ddx3Y*, *Dusp4*, *Dusp14*, *Nnat*, *Otx2*, *Tec*, *TGFB2*, *Nes*, *Nuak1*, *Runx1* and *Tagin*, were selected, and their mRNA expression levels confirmed by orthogonal real-time PCR profiling (Fig 6B). Bioinformatics analysis of these hits revealed that they belong to 22 different classes (including cytoskeletal proteins, signalling molecules, cytokines and transcription factors) and are implicated in seven different molecular functions, including the TGF-β signalling pathway (Supplementary Fig S6B). Importantly, the pluripotency genes *Nanog* and *Oct4* were unaltered between WT, *Tab1*^*−/−*^ and *Map3k1*^*mPHD*^ ES cells growing in serum and LIF, indicating that the MEKK1 PHD is not critical for maintaining ES cells in a pluripotent state (Supplementary Fig S6C). WT, *Tab1*^*−/−*^ and *Map3k1*^*mPHD*^ ES cells had significantly reduced expression of *Nanog* and *Oct4* following 9 days of culture under conditions that induce ES cell differentiation (Supplementary Fig S6C). There were no significant differences in the proliferation of WT, *Tab1*^*−/−*^ and *Map3k1*^*mPHD*^ ES cells (Supplementary Fig S6D).

Bioinformatics analysis demonstrated that there is no significant difference in the expression of TGFβRs and EGFRs between WT and *Map3k1*^*mPHD*^ ES cells (Supplementary Fig S7A). Orthogonal real-time PCR analysis demonstrated that there was no gene expression difference in TGFβR expression between WT, *Tab1*^*−/−*^ and *Map3k1*^*mPHD*^ ES cells (Supplementary Fig S7B). When grown in the presence of serum and LIF, there is a small reduction in the amount of phospho-MEKK1 in *Tab1*^*−/−*^ and *Map3k1*^*mPHD*^ ES cells (Supplementary Fig S7C).

### The MEKK1 PHD and TAB1 signalling are critical for ES-cell differentiation

**Figure 7:**
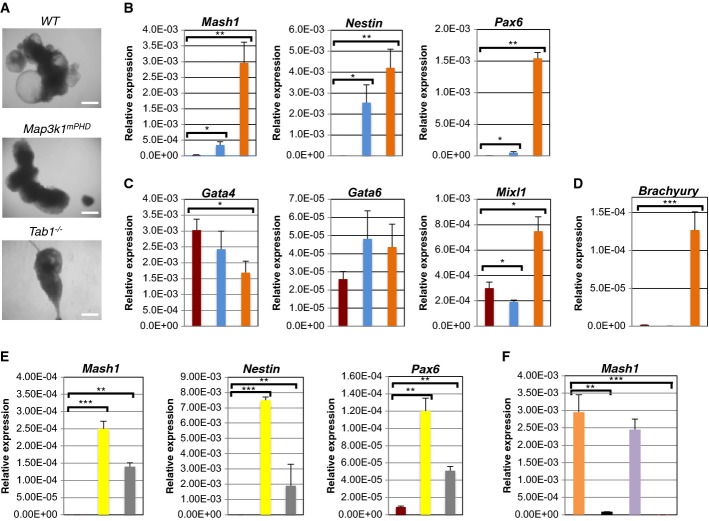
*Map3k1*^*mPHD*^ ES cells exhibit an altered differentiation pattern WT, *Map3k1*^*mPHD*^ and *Tab1*^*−/−*^ ES cells were plated under differentiation conditions without LIF for 6 or 9 days. A Pictures of EBs were taken using an Olympus light microscope after 9 days of differentiation and analysed using Image Pro-Software at 40× magnification. Scale bar is 250 μm. B–D (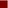) WT, (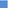) *Map3k1*^*mPHD*^ and (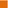) *Tab1*^*−/−*^ ES cells were plated under differentiation conditions for 6 days, and their RNAs analysed by real-time PCR with primers specific for (B) neuroectoderm, (C) endoderm and (D) mesoderm genes. E WT ES cells were differentiated for 6 days in the presence of (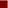) DMSO, (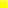) SB203580 or (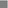) SB431542, and their RNAs analysed by real-time PCR with primers specific for neuroectoderm genes. F *Tab1*^*−/−*^ ES cells were transfected with (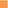) CMV, (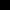) CMV TAB1 or (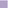) CMV mTAB1 and used alongside (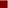) WT ES cells in differentiation assays for 6 days, mRNA was extracted and their RNAs analysed by real-time PCR with primers specific for the neuroectoderm gene *Mash1*. Data information: The average relative expression (± SEM) of the indicated gene mRNA from three independent experiments was statistically analysed, where appropriate, by two-tailed Student's *t*-test (*P ≤ 0.05; **P ≤ 0.01; ***P ≤ 0.001). Source data are available online for this figure.

We performed embryoid body (EB) formation assays with WT, *Tab1*^*−/−*^ and *Map3k1*^*mPHD*^ ES cells to determine whether the MEKK1 PHD motif and TAB1 control ES-cell differentiation (Doetschman *et al*, 1985; Dang *et al*, 2002; Wu *et al*, 2010). EBs were formed by WT, *Map3k1*^*mPHD*^ and *Tab1*^*−/−*^ ES cells, and also by ES cells treated with p38 or TGFβR inhibitors (Fig 7A and unpublished observations). By contrast, long-term treatment of WT ES cells with UBE2N, EGFR or JNK inhibitors reduced ES-cell viability, preventing them from forming EBs in long-term culture (unpublished observations). To assess whether *Map3k1*^*mPHD*^ and *Tab1*^*−/−*^ ES cells exhibit defective ES-cell differentiation, days 6 and 9 EBs were analysed by real-time PCR analysis using neuroectoderm (*Nestin*, *Pax6* and *Mash1*), endoderm (*Mixl1*, *Gata6* and *Gata4*) and mesoderm (*Brachyury*) markers (Fig 7B–D and Supplementary Fig S8A–C). On day 6 post-differentiation, *Map3k1*^*mPHD*^ and *Tab1*^*−/−*^ ES cells displayed significantly elevated expression of the neuroectoderm gene markers *Nestin*, *Pax6* and *Mash1* (Fig 7B), while, by contrast, at day 9, the mesoderm gene marker *Brachyury* was significantly reduced in both *Map3k1*^*mPHD*^ and *Tab1*^*−/−*^ relative to WT ES cells (Supplementary Fig S8C). Chemical inhibition of p38 or TGFβR also elevated expression of the neuroectoderm gene markers at day 6 and reduced day 9 *Brachyury* expression (Fig 7E and Supplementary Fig S8D).

To assess whether changes in ES cell neuroectoderm and mesoderm gene markers were due to altered TAB1 ubiquitination, *Tab1*^*−/−*^ ES cells were transfected with TAB1 or mTAB1 (Supplementary Fig S8E) and EB assays performed (Fig 7F, Supplementary Figs S8F and S9). *Tab1*^*−/−*^ ES cells reconstituted with TAB1, but not mTAB1, expressed similar levels of neuroectoderm and mesoderm markers as WT ES cells (Fig 7F, Supplementary Figs S8F and S9).

### Analysis of *Map3k1*^*mPHD*^ and *Tab1*^*−/−*^ ES-cell tumourigenicity

**Figure 8:**
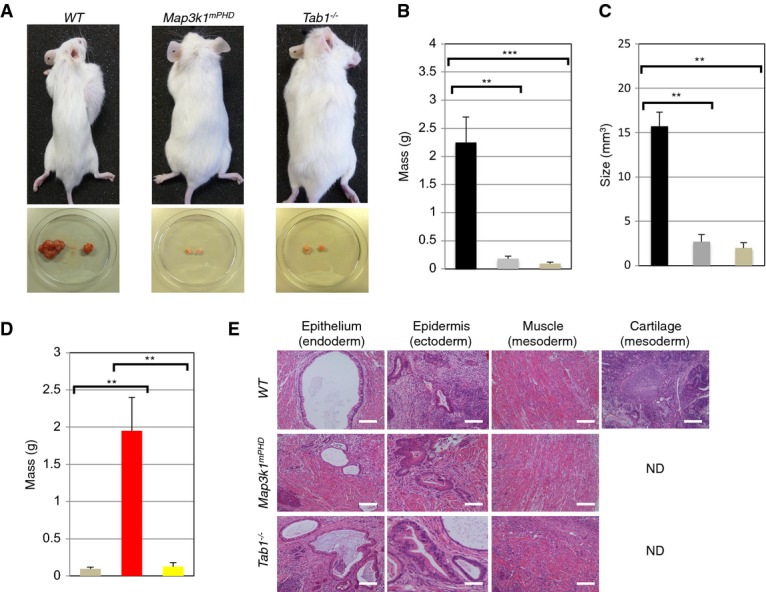
Regulation of ES-cell tumourigenesis by the MEKK1 PHD Analysis of WT, *Map3k1*^*mPHD*^ and *Tab1*^*−/−*^ ES-cell teratoma formation in NOD.CB17-*Prkdc*^*scid*^/lcrCrl recipient mice.Mass (g) of (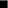) WT, (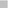) *Map3k1*^*mPHD*^ and (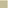) *Tab1*^*−/−*^ tumours formed in the above mouse strain 5 weeks post-transplantation. The average mass (± SEM) of tumours from 3 independent experiments was statistically analysed, where appropriate, by two-tailed Student's *t*-test (**P ≤ 0.01; ***P ≤ 0.001).The average size (± SEM) of (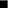) WT, (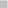) *Map3k1*^*mPHD*^ and (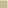) *Tab1*^*−/−*^ tumours from 3 independent experiments was statistically analysed, where appropriate, by two-tailed Student's *t*-test (**P ≤ 0.01).*Tab1*^*−/−*^ ES cells were transfected with (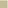) CMV, (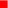) CMV TAB1 or (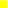) CMV mTAB1 expression vectors and their tumourigenic potential was analysed as above. Average results (SEM) from three independent experiments were statistically analysed by two-tailed Student's *t*-test (**P ≤ 0.01).Analysis of WT, *Map3k1*^*mPHD*^ and *Tab1*^*−/−*^ ES-cell teratomas. Tumours were extracted and analysed by H&E staining (ND indicates tissue not detected). Pictures were taken using an Olympus light microscope, and pictures were analysed using Image Pro-Software at 40× magnification. Scale bar is 70 μm. Data information: Results are representative of three independent experiments. Source data are available online for this figure.

We tested whether transplanted *Map3k1*^*mPHD*^ and *Tab1*^*−/−*^ ES cells, when injected into immunodeficient NOD.CB17-*Prkdc*^*scid*^/lcrCrl recipient mice, exhibited defective teratoma formation. While WT transplanted ES cells were able to form large tumours within 5 weeks of transplantation, *Map3k1*^*mPHD*^ or *Tab1*^*−/−*^ ES cells produced tumours of much smaller size and mass (Fig 8A–C). Add-back of TAB1, but not mTAB1, into *Tab1*^*−/−*^ ES cells restored tumor development to WT levels (Fig 8D). Histological analysis of *Map3k1*^*mPHD*^ and *Tab1*^*−/−*^ ES-cell tumours revealed a deficit in the formation of cartilage-like tissues (Fig 8E).

### Analysis of the *Map3k1*^*mPHD*^ mutation in mice

**Figure 9:**
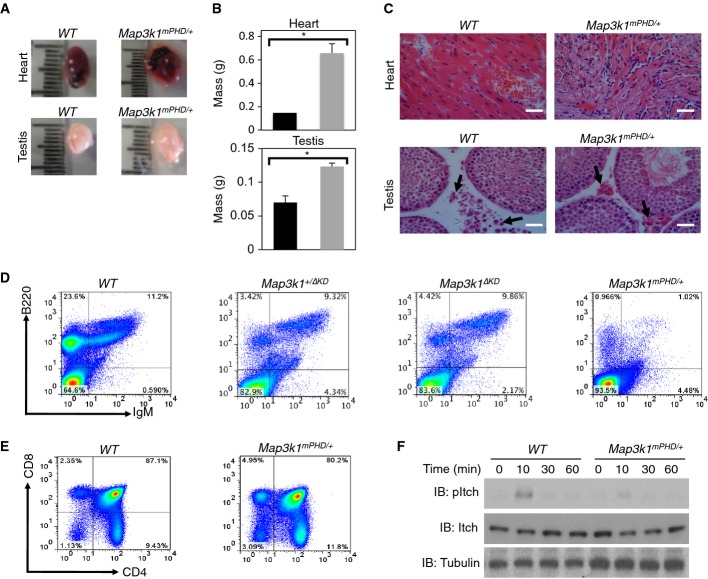
Analysis of *Map3k1*^*mPHD/*+^ mice Testis and heart were extracted from WT and *Map3k1*^*mPHD/*+^ mice.Quantitation of the mass (g) of testis and heart tissues from (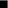) WT and (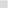) *Map3k1*^*mPHD/*+^ mice. The average mass (± SEM) of testis and heart from 3 independent experiments was statistically analysed, where appropriate, by two-tailed Student's *t*-test (*P ≤ 0.05).Testis and heart were extracted from WT and *Map3k1*^*mPHD/+*^ mice. H&E-stained cardiac and testis tissue sections were prepared from WT and *Map3k1*^*mPHD/*+^ mice. Pictures were taken using an Olympus light microscope, and pictures were analysed using Image Pro-Software at 40× magnification. Scale bar is 50 μm. Arrows indicate Leydig cells in the testis.Bone marrow was harvested from WT, *Map3k1*^*mPHD/*+^, *Map3k1*^+*/ΔKD*^ and *Map3k1*^*ΔKD*^ mice. Cells were stained with antibodies for the B-cell markers B220 and IgM and analysed by FACS.Splenocytes from WT and *Map3k1*^*mPHD/*+^ mice were stained with antibodies for the T-cell markers CD4 and CD8 and analysed by FACS.T cells were purified from WT and *Map3k1*^*m*^^*PHD*^^*/*+^ mice and costimulated with anti-CD3 and anti-CD28 antibodies for 10, 30 and 60 min or left unstimulated. Lysates were made, and IB was performed using the indicated antibodies. Data information: Results are representative of three experiments. Source data are available online for this figure.

*Map3k1*^*mPHD*^ mice are non-viable due to early embryonic lethality, so we analysed *Map3k1*^*mPHD/+*^ heterozygote mice to understand the developmental function of the MEKK1 PHD motif. *Map3k1*^*mPHD/+*^ mice have significantly enlarged testes and hearts (Fig 9A and B). Since *Mekk1*^*−/−*^ mice exhibit cardiac abnormalities (Minamino *et al*, 1999, 2002), we analysed cardiac tissue from *Map3k1*^*mPHD/+*^ mice. H&E staining revealed extensive fibrosis and cardiac muscle damage relative to WT mice, and cardiac enlargement (Fig 9C). Since *Map3k1*^*ΔKD*^ mice have minor abnormalities in the testis, we analysed testis morphology and spermatogenesis of male *Map3k1*^*mPHD/+*^ mice (Warr *et al*, 2011). H&E staining showed condensed and reduced numbers of Leydig cells (Fig 9C).

Since *Map3k1*^*ΔKD*^ mice have defects in B- and T-cell signalling (Gao *et al*, 2004; Gallagher *et al*, 2007), we analysed lymphocytes from *Map3k1*^*mPHD/+*^ mice (Fig 9D and E, and Supplementary Fig S10A). Bone marrow cells from *Map3k1*^*mPHD/+*^ mice were stained with antibodies for mature B-cell markers (B220 and IgM), pro-B-cell markers (IL-7R and CD34), pre-B-cell markers (CD45 and CD38) and immature B-cell markers (CD45 and IgM). *Map3k1*^*mPHD/+*^ mice exhibited reduced numbers of pre-B-cells, immature B cells and mature B cells (Fig 9D and Supplementary Fig S10A), in contrast to *Map3k1*^*+/ΔKD*^ or *Map3k1*^*ΔKD*^ mice, which were similar in numbers to WT (Fig 9E). Thymocytes were analysed for the T-cell markers CD4 and CD8 to determine whether the MEKK1 PHD domain is also essential for T-cell development. No significant differences between the numbers of WT, *Map3k1*^*+/ΔKD*^, *Map3k1*^*ΔKD*^ and *Map3k1*^*mPHD/+*^ single-positive or double-positive thymocytes or total numbers of mature T cells were observed, and the size of the thymus is normal in *Map3k1*^*mPHD/+*^ mice (Fig 9E and Supplementary Fig S10B and C). To analyse whether MEKK1 might be important for MEKK1-dependent regulation of HECT E3 Ub ligase Itch (Gallagher *et al*, 2006; Enzler *et al*, 2009), purified T cells from WT and *Map3k1*^*mPHD/+*^ mice were costimulated by anti-CD3 and anti-CD28 antibodies. Itch phosphorylation was significantly reduced in *Map3k1*^*mPHD/+*^ relative to WT T cells (Fig 9F).

## Discussion

We report that TAB1 is a substrate for non-canonical ubiquitination mediated by the MEKK1 PHD and that this modification enhances MAPK activation by EGF and TGF-β. The kinase activity of MEKK1 is well documented for its role in the regulation of MAP2Ks, and detailed analysis of *Map3k1*^*ΔKD*^ mice has demonstrated important roles for MEKK1 in lymphocyte effector responses, keratinocyte migration and eyelid fusion (Xia *et al*, 2000; Zhang *et al*, 2003; Gao *et al*, 2004; Gallagher *et al*, 2007). Consistent with previous reports, mutation of the MEKK1 PHD reduced its auto-ubiquitination, but had comparatively little impact upon MEKK1 kinase activity (Lu *et al*, 2002; Witowsky & Johnson, 2003). Yet, *Map3k1*^*mPHD*^ ES cells were defective in MAPK activation in response to TGF-β, EGF and microtubule disruption by nocodazole. Thus, the MEKK1 PHD has important signalling functions that are not identical to those of the kinase domain.

Protein microarray screening identified several novel targets for the E3 Ub ligase activity of the MEKK1 PHD. From these targets, we focused on TAB1, since it is a critical adaptor in the TGF-β signalling pathway, and confirmed it as a MEKK1 PHD target protein that was ubiquitinated in WT, but not *Map3k1*^*mPHD*^, cells following TGF-β or EGF stimulation. TAB2 and TAB3 were not substrates for the MEKK1 PHD motif in our protein array analysis. Analysis of *Tab1*^*−/−*^ ES cells revealed that TAB1 was required for EGF- and TGF-β-stimulated TAK1 and MAPK activation. Mapping of the TAB1 residues subjected to ubiquitination by the MEKK1 PHD motif identified TAB1 lysines 294, 319, 335 and 350 whose replacement with alanines abolished TAB1 ubiquitination. TAB1 is known to bind and activate TAK1 or it may signal independently of TAK1 by binding p38 MAPK (Shibuya *et al*, 1996; Wang *et al*, 2001; Ge *et al*, 2002; Kang *et al*, 2006). MEKK1 binds and ubiquitinates TAB1 via its PHD domain to enhance the molecular interaction between TAB1 and TAK1, and the K294A, K319A, K335A and K350A substitutions also diminished the binding of TAB1 to TAK1 and inhibited TAK1 phosphorylation after EGF and TGF-β stimulation. TAB2, although not a MEKK1 PHD substrate, can be recruited to the TAB1:MEKK1 complex, and this interaction is dependent upon the presence of an intact Ub binding ZnF motif within TAB2 (Kanayama *et al*, 2004). Recruitment of TAB2 to the MEKK1:TAB1 signalling complex may facilitate further downstream signalling. Our results reveal that TAB1 ubiquitination is the major conduit for the signalling function of the MEKK1 PHD from TGFβRs. The interdependency between the MEKK1 PHD and TAK1 also explains why either genetic disruption of the MEKK1 PHD or chemical inhibition of TAK1 kinase activity leads to loss of MAPK activation following EGF or TGF-β stimulation of ES cells. Our results suggest that UBE2N is critical for TAK1 and MAPK activation in response to TGF-β. We also suggest that TRAF2 is not critical for TGF-β signalling, unlike its important roles in TNF and CD40 signal transduction (Karin & Gallagher, 2009). Indeed, TRAF6, but not TRAF2, is critical for TGFβR-dependent MAPK activation (Yamashita *et al*, 2008).

Interestingly, the MEKK1 PHD and TAB1 repress neuroectoderm marker expression and enhance long-term mesoderm gene marker expression as ES cells differentiate into EBs. p38α is known to play a role in ES-cell neuroectoderm and mesoderm differentiation (Gaur *et al*, 2010; Barruet *et al*, 2011). Similarly, *Jnk1* and *Jnk2* double deficiency in ES cells reveals a role for the JNK MAPKs in promoting ES-cell differentiation (Xu & Davis, 2010). To date, no compound *Jnk* and *p38*α ES-cell mutations have been reported that would mimic the effects of the MEKK1 PHD or TAB1 ablation. Transplantation of *Tab1*^*−/−*^ or *Map3k1*^*mPHD*^ ES cells into NOD.CB17-*Prkdc*^*scid*^/lcrCrl mice results in tumours of altered tissue composition, reduced size and mass. Importantly, reintroduction of TAB1, but not lysine mutated TAB1, into *Tab1*^*−/−*^ ES cells was able to restore normal ES-cell differentiation and tumour formation, indicating that the TAB1 lysines ubiquitinated by the MEKK1 PHD are critical for its function in ES-cell differentiation and tumour formation.

The analysis of *Map3k1*^*mPHD*^ mice has been complicated by their early embryonic lethality, which is a more severe phenotype than the partial lethality observed in *Map3k1*^*ΔKD*^ mice (Bonnesen *et al*, 2005). Alterations in the regulation of the Ub-proteasome system in combination with aberrant MAPK activation in ES cells provide an explanation for this more severe phenotype (Lu *et al*, 2002). However, *Map3k1*^*mPHD/+*^ mice are viable, and their analysis revealed defects in B-cell development, TCR signal transduction, cardiac tissue and testis development. These results reinforce the critical importance of the MEKK1 PHD in mammalian biology.

## Materials and Methods

### ES cell gene targeting and mice

*Map3k1* kinase-deficient mice (*Map3k1*^*ΔKD*^) were generated as previously described (Gao *et al*, 2004). To create *Map3k1*^*mPHD*^ gene knockin mice, a targeting vector containing a *loxP*-flanked neomycin resistance cassette and a mutated exon 7 was inserted into the *Map3k1* locus on chromosome 13. Targeted *Map3k1*^*mPHD/+*^ and *Map3k1*^*mPHD*^ ES cells were generated according to standard procedures (Gossler *et al*, 1986) and genotyped by Southern blotting or genomic PCR (Ledermann, 2000; Xia *et al*, 2000). Four independently generated *Map3k1*^*mPHD*^ knockin ES cell clones were injected into C57BL/6 blastocysts and the resulting transgenics were genotyped by PCR (Zhang *et al*, 2003). To create *Tab1*^*−/−*^ gene knockout ES cells, a targeting vector containing a *loxP*-flanked neomycin resistance cassette and a mutated exon 1 was inserted in the *Tab1* locus on chromosome 15. Targeted ES cells were generated according to standard procedures (Gossler *et al*, 1986) and genotyped by Southern blotting or genomic PCR (Ledermann, 2000). NOD.CB17-*Prkdc*^*scid*^/lcrCrl mice were purchased from Charles River Laboratories. All mice were bred and maintained under pathogen-free conditions in conventional barrier protection in accordance with the guidelines of the Home Office and Imperial College London.

### Reagents and antibodies

Phospho-SMAD antibody sampler kit (9963) was from Cell Signaling. Mouse anti-TRAF2 (sc-7346), rabbit anti-STAM (sc-33588), goat anti-TAB1 (sc-6052), rabbit anti-MEKK1 (sc-252) and rabbit anti-Abin1 (or TNIP1) (sc-134660) antibodies were from Santa Cruz Biotechnology. Mouse anti-HA (32-6700), mouse anti-Myc (R950-25) and mouse anti-Ub (13-1600) antibodies were from Invitrogen. Rabbit anti-p38 (9212), rabbit anti-pp38 (9211), rabbit anti-SAPK/JNK (9252), rabbit anti-Itch (12117), rabbit anti-TAK1 (4505), rabbit anti-pTAK1 (4508), rabbit anti-pSAPK/JNK (9251) and rabbit anti-Lys63-linked Ub (5621) antibodies were from Cell Signaling. Rabbit anti-pItch (AB10050) antibody was purchased from Merck Millipore. Mouse anti-p-ERK (M8159), rabbit anti-ERK (M5670), mouse anti-tubulin (T5168), mouse anti-T7 (T8823) and rabbit anti-Flag (F7425) antibodies were from Sigma. Anti-CD3 (555273) and anti-CD28 (347690) antibodies were from BD Biosciences. pCMV-Myc mouse MEKK1 and pCMV-Myc mouse MEKK1 C438A, I440A plasmids were purchased from GenScript. pCMV6-XL4 TRAF2, pCMV6-XL4 TNIP2 and pCMV6-Myc TNIP1 were from Origene. pCMV TAB1 and pCMV mTAB1 were purchased from GenScript. Rabbit anti-pMEKK1 was custom made by ThermoScientific. Rabbit anti-phospho MEKK1 was generated as previously described (Matsuzawa *et al*, 2008). TGF-β and EGF (100-35) were purchased from Peprotech. Chemical inhibitors include AG-490 (EGFR inhibitor, Santa Cruz Biotechnology), NSC697923 (UBE2N inhibitor, Millipore), (5Z)-7-Oxozeaenol (TAK1 inhibitor, Sigma), SB431542 (TGFβR inhibitor, Sigma) and SB203580 (p38 MAPK inhibitor, Sigma). Nocodazole and sorbitol were purchased from Sigma.

### Cells and cell culture conditions

HEK 293 cells were maintained in DMEM (22320, Invitrogen) supplemented with 10% FBS (SH3007003, Thermo Scientific) and antibiotics in a humidified atmosphere at 37°C. Cells were passaged every 2–3 days when approaching full confluence. T cells were isolated from mouse splenocytes using CD4 Microbeads (130-049-201, Miltenyi Biotec) according to the manufacturer's instructions (Gallagher *et al*, 2007). Mouse ES cells were grown in mESC medium (Knockout™ DMEM supplemented with 10% FBS, non-essential amino acids solution, l-glutamine, recombinant human LIF and 2-mercaptoethanol) in a humidified atmosphere at 37°C. Cells were passaged every 2 days.

### EB formation assays

ES cells were plated in low attachment 6-well plates in mESC medium with 5% FBS and no LIF at a density of 1 × 10^5^ cells per well (Doetschman *et al*, 1985; Dang *et al*, 2002; Wu *et al*, 2010). They were collected 6 or 9 days later for RNA extraction.

### Tumourigenesis assays

Pluripotent ES cells were injected subcutaneously into the flank of NOD.CB17-*Prkdc*^*scid*^/lcrCrl mice (1 × 10^6^ cells in PBS/animal) (Dressel *et al*, 2008). Mice were monitored daily for 5 weeks before the animals were culled and tumours extracted.

### Yeast two-hybrid

Y190 yeast were transformed and grown as previously described (Gallagher *et al*, 2007).

### Transfection

HEK 293 cells were plated in 6-well plates at a density of 1 × 10^6^ cells per well. The following day cells were transfected with Lipofectamine 2000 (11668-019, Invitrogen) or Jet Prime (114-07, Polyplus) transfection reagents according to the manufacturer's instructions. Cells were collected and lysed 48 h later. ES cells were transfected using Xfect Mouse Embryonic Stem Cell Transfection Reagent (631321, Clontech) according to the manufacturer's protocols.

### Ubiquitination and kinase assays

TRAF2, TNIP1, TNIP2, TAB1 and STAM1 were overexpressed in HEK 293 cells, immunoprecipitated, washed extensively and protein eluted. Subsequently, they were incubated for 1 h at 37°C with the ubiquitination assay enzymes E1 (100 nM), UBE2N:UBE2V1 (0.36 μM) or UBE2D2 (0.5 μM), Ub or no K Ub (150 μM) and ATP, with or without WT MEKK1 PHD (100 ng) or MEKK1 mPHD (100 ng) (Matsuzawa *et al*, 2008). All ubiquitination assay reagents were from Boston Biochem. Kinase assays were performed as previously described (Gallagher *et al*, 2002).

### Western blotting and immunoprecipitation

Cells were lysed in whole-cell lysis buffer (50 mM Tris pH 7.6, 150 mM NaCl and 1% Triton X-100) (Gao *et al*, 2004). For detection of ubiquitination, 20 mM N-ethylmaleimide (NEM) (E1271, Sigma) was added to the buffer. For immunoprecipitation, cells were lysed in buffer containing 20 mM Tris pH 7.6, 120 mM NaCl, 0.5 mM EDTA, 1.5 mM MgCl_2_ and 0.5% Triton X-100. All buffers were supplemented with protease and phosphatase inhibitors (Sigma). Following cell lysis, proteins were resolved in SDS polyacrylamide gels and transferred to PVDF membranes, blocked in 5% milk, incubated with specific primary and secondary antibodies and detected with ECL solution (32106, Pierce) or immunoprecipitated with 1–2 μg of the antibody of interest overnight at 4°C (Gao *et al*, 2004). Immunoprecipitates were captured with protein A/G Plus agarose beads (sc-2003, Santa Cruz). Beads were washed three times in lysis buffer without Triton X-100, and proteins were eluted in elution buffer (1858606, Pierce) or released by boiling in Laemmli Sample buffer (161-0737, Bio-Rad).

### Protein purification

BL21 (DE3) *E. coli* cells were transformed with pGEX KG MEKK1 PHD and were grown in Luria Broth (LB) media at 37°C (Lu *et al*, 2002). At an OD_600_ of 0.6 protein production was induced with 1 mM IPTG. Cells were further grown for approximately 18 h at 18°C. After bacterial cell lysis, supernatant was applied to GST column (GE Healthcare) to capture MEKK1 PHD. Protein was eluted from the column with a buffer containing PBS, 50 mM reduced glutathione and 1 mM DTT, pH 7.8. Full-length His-MEKK1, His-MEKK1 mPHD, His-TAB1 and His-TAK1 were overexpressed and purified from Sf9 cells as previously described (Gallagher *et al*, 2002). Proteins expressed in HEK 293 cells were IP, bound to HiTrap Protein G columns (GE Healthcare Life Sciences), washed extensively and then eluted for further assays according to the manufacturer's protocols.

### Proliferation assays

Proliferation was measured using a CellTrace CFSE Cell Proliferation kit (Life Technologies) according to the manufacturer's protocols.

### Protein microarray

UBE1 (100 nM), UBE2N:UBE2V1 (500 nM) and purified MEKK1 PHD-GST (50 or 250 nM) were used in ubiquitination assays in the presence of biotin-Ub (100 μg/ml) (Invitrogen). Arrays probed with buffer only or UBE1 and UBE2N:UBE2V1 without MEKK1 PHD served as negative controls. Ubiquitination of the immobilised proteins on the arrays treated with UBE1, UBE2N:UBE2V1, and MEKK1 PHD-GST was evaluated by the *Z*-score and background subtracted signal values within the array relative to the control assays.

### Real-time PCR

Total RNA was extracted using the RNeasy Midi kit (Qiagen) according to the manufacturer's instructions. RNA was converted to cDNA using the High-Capacity cDNA Reverse Transcription kit (Applied Biosystems). cDNA was amplified using SYBR Green PCR Master Mix (Applied Biosystems) and primer pairs of interest (Supplementary Table S4) (Gao *et al*, 2004).

### Affymetrix microarray global gene expression screening

Total RNA was converted to cRNA using the WT Expression kit (Ambion). Quality of cRNA was assessed with a 2100 Bioanalyser. cRNA was converted to second-strand cDNA using the WT Expression kit (Ambion). cDNA was fragmented and labelled using the GeneChip WT Terminal Labelling kit (Affymetrix). Labelled cDNA was hybridised to a GeneChip Mouse Gene 1.0 ST Array. GeneSpring software was used for data analysis and quality control. Probes were normalised by quantile normalisation among all microarray data.

### Molecular modelling of the MEKK1 PHD

The amino acid sequence of mouse MEKK1 PHD (residues 432–485) was submitted to the Phyre^2^ server to produce the WT MEKK1 PHD model (Kelley & Sternberg, 2009). The side chain of residues 438 and 440 was then manually truncated to an alanine to create the mutant MEKK1 PHD model.

### Bioinformatics

GeneSpring software was used to create heat maps of the Protoarray and Affymetrix data according to the software vendor's instructions. Ingenuity IPA and iReport were used according to the software vendor's protocols for bioinformatics analysis.

### Accession numbers

ArrayExpress accession: E-MTAB-1679. IMEx accession: IM-22822.

## References

[b1] Barruet E, Hadadeh O, Peiretti F, Renault VM, Hadjal Y, Bernot D, Tournaire R, Negre D, Juhan-Vague I, Alessi MC, Binetruy B (2011). p38 mitogen activated protein kinase controls two successive-steps during the early mesodermal commitment of embryonic stem cells. Stem Cells Dev.

[b2] Bonnesen B, Orskov C, Rasmussen S, Holst PJ, Christensen JP, Eriksen KW, Qvortrup K, Odum N, Labuda T (2005). MEK kinase 1 activity is required for definitive erythropoiesis in the mouse fetal liver. Blood.

[b3] Cardone MH, Salvesen GS, Widmann C, Johnson G, Frisch SM (1997). The regulation of anoikis: MEKK-1 activation requires cleavage by caspases. Cell.

[b4] Chen Z, Gibson TB, Robinson F, Silvestro L, Pearson G, Xu B, Wright A, Vanderbilt C, Cobb MH (2001). MAP kinases. Chem Rev.

[b5] Dang SM, Kyba M, Perlingeiro R, Daley GQ, Zandstra PW (2002). Efficiency of embryoid body formation and hematopoietic development from embryonic stem cells in different culture systems. Biotechnol Bioeng.

[b6] Doetschman TC, Eistetter H, Katz M, Schmidt W, Kemler R (1985). The *in vitro* development of blastocyst-derived embryonic stem cell lines: formation of visceral yolk sac, blood islands and myocardium. J Embryol Exp Morphol.

[b7] Dressel R, Schindehutte J, Kuhlmann T, Elsner L, Novota P, Baier PC, Schillert A, Bickeboller H, Herrmann T, Trenkwalder C, Paulus W, Mansouri A (2008). The tumorigenicity of mouse embryonic stem cells and *in vitro* differentiated neuronal cells is controlled by the recipients' immune response. PLoS ONE.

[b8] Enzler T, Chang X, Facchinetti V, Melino G, Karin M, Su B, Gallagher E (2009). MEKK1 binds HECT E3 ligase Itch by its amino-terminal RING motif to regulate Th2 cytokine gene expression. J Immunol.

[b9] Gallagher ED, Xu S, Moomaw C, Slaughter CA, Cobb MH (2002). Binding of JNK/SAPK to MEKK1 is regulated by phosphorylation. J Biol Chem.

[b10] Gallagher E, Gao M, Liu YC, Karin M (2006). Activation of the E3 ubiquitin ligase Itch through a phosphorylation-induced conformational change. Proc Natl Acad Sci USA.

[b11] Gallagher E, Enzler T, Matsuzawa A, Anzelon-Mills A, Otero D, Holzer R, Janssen E, Gao M, Karin M (2007). Kinase MEKK1 is required for CD40-dependent activation of the kinases Jnk and p38, germinal center formation, B cell proliferation and antibody production. Nat Immunol.

[b12] Gao M, Labuda T, Xia Y, Gallagher E, Fang D, Liu YC, Karin M (2004). Jun turnover is controlled through JNK-dependent phosphorylation of the E3 ligase Itch. Science.

[b13] Gao M, Karin M (2005). Regulating the regulators: control of protein ubiquitination and ubiquitin-like modifications by extracellular stimuli. Mol Cell.

[b14] Gaur M, Ritner C, Sievers R, Pedersen A, Prasad M, Bernstein HS, Yeghiazarians Y (2010). Timed inhibition of p38MAPK directs accelerated differentiation of human embryonic stem cells into cardiomyocytes. Cytotherapy.

[b15] Ge B, Gram H, Di Padova F, Huang B, New L, Ulevitch RJ, Luo Y, Han J (2002). MAPKK-independent activation of p38alpha mediated by TAB1-dependent autophosphorylation of p38alpha. Science.

[b16] Gibson S, Widmann C, Johnson GL (1999). Differential involvement of MEK kinase 1 (MEKK1) in the induction of apoptosis in response to microtubule-targeted drugs versus DNA damaging agents. J Biol Chem.

[b17] Gossler A, Doetschman T, Korn R, Serfling E, Kemler R (1986). Transgenesis by means of blastocyst-derived embryonic stem cell lines. Proc Natl Acad Sci USA.

[b18] Hochstrasser M (1996). Protein degradation or regulation: Ub the judge. Cell.

[b19] Ikeda F, Crosetto N, Dikic I (2010). What determines the specificity and outcomes of ubiquitin signaling?. Cell.

[b20] Kanayama A, Seth RB, Sun L, Ea CK, Hong M, Shaito A, Chiu YH, Deng L, Chen ZJ (2004). TAB2 and TAB3 activate the NF-kappaB pathway through binding to polyubiquitin chains. Mol Cell.

[b21] Kang YJ, Seit-Nebi A, Davis RJ, Han J (2006). Multiple activation mechanisms of p38alpha mitogen-activated protein kinase. J Biol Chem.

[b22] Karin M, Gallagher E (2005). From JNK to pay dirt: jun kinases, their biochemistry, physiology and clinical importance. IUBMB Life.

[b23] Karin M, Gallagher E (2009). TNFR signaling: ubiquitin-conjugated TRAFfic signals control stop-and-go for MAPK signaling complexes. Immunol Rev.

[b24] Kelley LA, Sternberg MJ (2009). Protein structure prediction on the Web: a case study using the Phyre server. Nat Protoc.

[b25] Kravtsova-Ivantsiv Y, Ciechanover A (2012). Non-canonical ubiquitin-based signals for proteasomal degradation. J Cell Sci.

[b26] Ledermann B (2000). Embryonic stem cells and gene targeting. Exp Physiol.

[b27] Lu Z, Xu S, Joazeiro C, Cobb MH, Hunter T (2002). The PHD domain of MEKK1 acts as an E3 ubiquitin ligase and mediates ubiquitination and degradation of ERK1/2. Mol Cell.

[b28] Lu Z, Hunter T (2009). Degradation of activated protein kinases by ubiquitination. Annu Rev Biochem.

[b29] Matsuzawa A, Tseng PH, Vallabhapurapu S, Luo JL, Zhang W, Wang H, Vignali DA, Gallagher E, Karin M (2008). Essential cytoplasmic translocation of a cytokine receptor-assembled signaling complex. Science.

[b30] Minamino T, Yujiri T, Papst PJ, Chan ED, Johnson GL, Terada N (1999). MEKK1 suppresses oxidative stress-induced apoptosis of embryonic stem cell-derived cardiac myocytes. Proc Natl Acad Sci USA.

[b31] Minamino T, Yujiri T, Terada N, Taffet GE, Michael LH, Johnson GL, Schneider MD (2002). MEKK1 is essential for cardiac hypertrophy and dysfunction induced by Gq. Proc Natl Acad Sci USA.

[b32] Ono K, Ohtomo T, Sato S, Sugamata Y, Suzuki M, Hisamoto N, Ninomiya-Tsuji J, Tsuchiya M, Matsumoto K (2001). An evolutionarily conserved motif in the TAB1 C-terminal region is necessary for interaction with and activation of TAK1 MAPKKK. J Biol Chem.

[b33] Raman M, Chen W, Cobb MH (2007). Differential regulation and properties of MAPKs. Oncogene.

[b34] Reyes-Turcu FE, Ventii KH, Wilkinson KD (2009). Regulation and cellular roles of ubiquitin-specific deubiquitinating enzymes. Annu Rev Biochem.

[b35] Shibuya H, Yamaguchi K, Shirakabe K, Tonegawa A, Gotoh Y, Ueno N, Irie K, Nishida E, Matsumoto K (1996). TAB1: an activator of the TAK1 MAPKKK in TGF-beta signal transduction. Science.

[b36] Shim JH, Xiao C, Paschal AE, Bailey ST, Rao P, Hayden MS, Lee KY, Bussey C, Steckel M, Tanaka N, Yamada G, Akira S, Matsumoto K, Ghosh S (2005). TAK1, but not TAB1 or TAB2, plays an essential role in multiple signaling pathways *in vivo*. Genes Dev.

[b37] Takaesu G, Kishida S, Hiyama A, Yamaguchi K, Shibuya H, Irie K, Ninomiya-Tsuji J, Matsumoto K (2000). TAB2, a novel adaptor protein, mediates activation of TAK1 MAPKKK by linking TAK1 to TRAF6 in the IL-1 signal transduction pathway. Mol Cell.

[b38] Walczak H, Iwai K, Dikic I (2012). Generation and physiological roles of linear ubiquitin chains. BMC Biol.

[b39] Wang C, Deng L, Hong M, Akkaraju GR, Inoue J, Chen ZJ (2001). TAK1 is a ubiquitin-dependent kinase of MKK and IKK. Nature.

[b40] Wang H, Matsuzawa A, Brown SA, Zhou J, Guy CS, Tseng PH, Forbes K, Nicholson TP, Sheppard PW, Hacker H, Karin M, Vignali DA (2008). Analysis of nondegradative protein ubiquitylation with a monoclonal antibody specific for lysine-63-linked polyubiquitin. Proc Natl Acad Sci USA.

[b41] Warr N, Bogani D, Siggers P, Brixey R, Tateossian H, Dopplapudi A, Wells S, Cheeseman M, Xia Y, Ostrer H, Greenfield A (2011). Minor abnormalities of testis development in mice lacking the gene encoding the MAPK signalling component, MAP3K1. PLoS ONE.

[b42] Weston CR, Davis RJ (2002). The JNK signal transduction pathway. Curr Opin Genet Dev.

[b43] Witowsky JA, Johnson GL (2003). Ubiquitylation of MEKK1 inhibits its phosphorylation of MKK1 and MKK4 and activation of the ERK1/2 and JNK pathways. J Biol Chem.

[b44] Wu J, Kubota J, Hirayama J, Nagai Y, Nishina S, Yokoi T, Asaoka Y, Seo J, Shimizu N, Kajiho H, Watanabe T, Azuma N, Katada T, Nishina H (2010). p38 Mitogen-activated protein kinase controls a switch between cardiomyocyte and neuronal commitment of murine embryonic stem cells by activating myocyte enhancer factor 2C-dependent bone morphogenetic protein 2 transcription. Stem Cells Dev.

[b45] Xia Y, Makris C, Su B, Li E, Yang J, Nemerow GR, Karin M (2000). MEK kinase 1 is critically required for c-Jun N-terminal kinase activation by proinflammatory stimuli and growth factor-induced cell migration. Proc Natl Acad Sci USA.

[b46] Xia Y, Karin M (2004). The control of cell motility and epithelial morphogenesis by Jun kinases. Trends Cell Biol.

[b47] Xia Y, Wang J, Xu S, Johnson GL, Hunter T, Lu Z (2007). MEKK1 mediates the ubiquitination and degradation of c-Jun in response to osmotic stress. Mol Cell Biol.

[b48] Xu P, Davis RJ (2010). c-Jun NH2-terminal kinase is required for lineage-specific differentiation but not stem cell self-renewal. Mol Cell Biol.

[b49] Yamaguchi K, Shirakabe K, Shibuya H, Irie K, Oishi I, Ueno N, Taniguchi T, Nishida E, Matsumoto K (1995). Identification of a member of the MAPKKK family as a potential mediator of TGF-beta signal transduction. Science.

[b50] Yamashita M, Fatyol K, Jin C, Wang X, Liu Z, Zhang YE (2008). TRAF6 mediates Smad-independent activation of JNK and p38 by TGF-beta. Mol Cell.

[b51] Yin Q, Lin SC, Lamothe B, Lu M, Lo YC, Hura G, Zheng L, Rich RL, Campos AD, Myszka DG, Lenardo MJ, Darnay BG, Wu H (2009). E2 interaction and dimerization in the crystal structure of TRAF6. Nat Struct Mol Biol.

[b52] Yujiri T, Fanger GR, Garrington TP, Schlesinger TK, Gibson S, Johnson GL (1999). MEK kinase 1 (MEKK1) transduces c-Jun NH2-terminal kinase activation in response to changes in the microtubule cytoskeleton. J Biol Chem.

[b53] Zhang L, Wang W, Hayashi Y, Jester JV, Birk DE, Gao M, Liu CY, Kao WW, Karin M, Xia Y (2003). A role for MEK kinase 1 in TGF-beta/activin-induced epithelium movement and embryonic eyelid closure. EMBO J.

