# Supplementary material for: The MEKK1 PHD ubiquitinates TAB1 to activate MAPKs in response to cytokines
Source: EMBO J. 2014 Sep 26;33(21):2581–96. doi: 10.15252/embj.201488351 (PMC4282369; doi:10.15252/embj.201488351)
Supplement: Supplementary file 1 [file embj0033-2581-sd1.pdf]

THE EMBO JOURNAL

Manuscript EMBO-2014-88351

The MEKK1 PHD ubiquitinates TAB1 to activate MAPKs in response to cytokines

Nikolaos Charlaftis, Tesha Suddason, Xuefeng Wu, Saba Anwar, Michael Karin

Corresponding author: Ewen Gallagher, Imperial College London

Review timeline:

Submission date:	27 February 2014
Editorial Decision:	26 March 2014
Revision received:	23 June 2014
Editorial Decision:	18 July 2014
Revision received:	13 August 2014
Accepted:	22 August 2014

Transaction Report:

(Note: With the exception of the correction of typographical or spelling errors that could be a source of ambiguity, letters and reports are not edited. The original formatting of letters and referee reports may not be reflected in this compilation.)

1st Editorial Decision 26 March 2014

Thank you for submitting your manuscript "The MEKK1 PHD ubiquitinates TAB1 to control stem cell differentiation and tumorigenesis" to The EMBO Journal editorial office.

We have now received the comments from three referees copied below for your information. As you will see, all the referees consider your findings potentially interesting and significant, but do ask for specific mechanistic experiments as well as for control experiments.

We would, therefore, like to invite you to revise your manuscript and we will consider publishing your work, in case you should be willing to extend the mechanistic insight how MEKK1 potentiates TAK1 kinase activity. This is most clearly highlighted by referee #3 and is fully in line with our own reading. In particular, although the manuscript already contains a lot of data, we would like to stress that providing some additional insights into how the K63 Ub chains on TAB1 result in enhanced TAK1 activation, would be an important prerequisite for further consideration.

In this respect, referee #3's insightful and constructive suggestions in specific points 1 and 4 should be followed and taken as incentive to extend biochemical evidence for TAK1 activation by ubiquitinated Tab1. This might include a dedicated interaction study to delineate the role of protein domains and Ub itself in stabilizing the ternary TAB1/TAK1/TAB2/3 complex. Additionally, to be more conclusive, the sequential ubiquitination/kinase reactions in E4i should definitely be extended and additional appropriate controls performed.

In addition, we would like to stress that also referee #1 had mechanistic concerns. While we do not necessarily agree with all of his requests in specific points 2/3, at least the kinase activity of C438.I440A mutant MEKK1 should be quantified - this also relates to referee #3's specific point 2. Moreover, mRNA microarray data could be better presented and detailed expression levels for Egf and Tgf family receptors provided.

I would, therefore, be pleased if you would invest the necessary time and efforts to address the reviewers' concerns and specifically focus on the major points outlined above.

We generally allow three months as standard revision time. In the light of the experiments requested here, we would however be open to extend the deadline in case this would be required to accommodate the requested experimentation.

Please note that during invited revisions competing manuscripts would have no negative impact in assessing the novelty of your revised study. However, we request that you contact the editor as soon as possible upon publication of any related work, to discuss how to proceed.

REFEREE COMMENTS

Referee #1:

In this manuscript, Charlaftis et al. examined the role of the MEKK1 PHD as an E3 Ub ligase in ES cells. First, the authors identified the key amino acid residues that are important for the function of the MEKK1 PHD as an E3 Ub ligase. Using knock-in ES cell lines, which have an inactive mutant PHD (Map3k1mPHD), they found that MEKK1 PHD controls downstream MAPK activation following TGF-beta, EGF and Nocodazole stimuli. Next, they performed large-scale protein microarray screening and identified TAB1 as a novel substrate for MEKK1 PHD. Moreover, they found that MEKK1 PHD-dependent K63-linked ubiquitination of TAB1 is critical for MAPK activation in response to EGF and TGF-beta. Furthermore, they showed that Map3k1mPHD or TAB1-/- ES cells have ability to produce tumors of much smaller size and mass compared to wild-type ES cells, suggesting that MEKK1 PHD and TAB1 are critical for ES cell differentiation. Although the topic is very interesting, the current version of this manuscript is immature and several points should be addressed before publication in the EMBO Journal.

Major points

1. There are too many 'unpublished data' in this manuscript (P9 L24, P10 L22, P13 L5, P13 L8, P13 L20, P13 L23, P14 L21, P15 L11). All 'unpublished data' should be displayed.

2. The role of MEKK1 kinase activity for MAPK signaling in ES cells is not clear. Although the authors examine the kinase activity of MEKK1 mPHD mutant in HEK293 cells (Fig 1b), MEKK1 kinase activity in ES cells is not examined. Is MEKK1 kinase activity involved in EGF or TGF-beta-induced MAPK signaling in ES cells? Also, does MEKK1 C438A, I440A double mutant have the kinase activity?

3. Although the authors argue that microarray data revealed normal expression of TGF-beta receptors and EGF family receptors in Map3k1mPHD ES cells, microarray data are not convincing to persuade the expression level of those receptors. Also, the authors should describe the expression level of those receptors in TAB1-/- ES cells.

4. In Fig 4a and Fig 4b, is TAK1 activation inhibited by those inhibitors?

Minor points

1. In Fig E4, immunoblot of TAB1 should be shown.

2. In Fig E7b, the result is presented without quantification. Quantification of the data from different experiments should be shown.

3. In Fig 6a, Fig E7c and Fig E7d, scale bars should be present. Also, molecular weight markers should be shown in western blot data.

4. In Fig E7d, the figure legend does not help much. What does the arrow indicate?

Referee #2:

MEKK1 (Map3k1) has E3 ligase activity through its PHD motif. To study the role of MEKK1 PHD motif, mutant PHD Map3k1 (with inactive PHD domain) ES cells and knockin mice were generated. Through microarray profiling of wild type and mutant PHD Map3k1 ES cells, TAB1 was identified as a novel MEKK1 ubiquitination substrate. TGFβ and EGF promoted the ubiquitination of TAB1 by MEKK1 PHD motif and activate phosphorylation of TAK1, p38, and JNK. Furthermore, MEKK1 was shown to regulate ES cell differentiation, embryonic body formation and tumorigenesis by ubiquitinating TAB1. In addition, mutant PHD Map3k1 knockin mice exhibited development defects in cardiac tissues, B cell development, spermatogenesis and TCR signaling. Overall, the study is very novel and revealed a novel substrate for MEKK1. The characterization of the role of MEKK1 PHD motif in ES cell culture, mouse development and tumorigenesis was well executed. There are some minor issues needs to be resolved before it can be published in EMBOJ.

1. In vitro ubiquitination assay in Fig E3b-f: The author should clearly describe or label which ubiquitination signal is from the substrates (e.g. TAB1 and others), but not from MEKK1 autoubiquitination.
2. The interaction between MEKK1 and TAB1 was shown under overexpression conditions (Fig E4b). The authors may want to prove this notion under the physiological conditions.
3. TAB1, but not mTAB1, restoration rescued the Mash1 level in TAB1 deficient ES cells in Fig. 6f. How about the levels of other defective genes, such as Nestin, Pax6?
4. To study the function of mutant PHD Map3k1 in ES cells, mutant PHD Map3k1 knockin ES cells have been generated. Are these cells homozygous? Since ES cell targeting usually generates heterozygous cells, it is encouraging to describe how homozygous ES cells are generated. The author should also provide the information about how to identify homozygous knockin ES cells. In Fig1d (Southern blotting), there is no clear labeling indicating which allele corresponds to which band.

Referee #3:

In this paper the authors have extended their characterization of the MEKK1/MAP3K1 MAP3K that can activate ERK, JNK and p38 MAPKs. They have previously shown that MAP3K1 is important for T/B cell activation, with the C-terminal kinase domain and its kinase activity being required. Here, they have examined the function of the PHD domain in the N-terminal half of MEKK1/MAP3K1, which has a RING domain like fold and is known to possess E3 ubiquitin ligase activity. For this purpose, they made a mutant MEKK1 lacking PHD function, by converting Cys438 and Ile440 to Ala, based on the predicted key structural roles for these residues in E2 binding and E3 activity, showing that transiently expressed full length mPHD MEKK1 lacked K63 chain autoubiquitylaton activity readily detectable with WT MEKK1. They went on to generate mPHD mutant mouse ES cells, as a prelude to making mPHD mice. Comparison of the mPHD ES cells with the corresponding WT ES cells showed that the PHD domain function was not required for hyperosmotic stress or activation of ERK, JNK and p38 MAPKs, but was required for TGFβ, and EGF activation of Tak1, JNK and p38 but not ERK MAPK. No change in stability of ERK, JNK or p38 proteins was noted in response to hyperosmotic stress, despite an earlier report that ERK is destabilized by MEKK1 in a PHD domain dependent manner, albeit in a different cell type. Next they used in vitro screening of 9400 arrayed human proteins to identify new MEKK1 E3 ligase substrates, identifying several candidates including TAB1, TNIP1 and 2, TRAF2, and STAM1, and showing that MEKK1 WT but not mPHD could promote K63 polyubiquitylation of these proteins in transfected 293T cell. Since TAB1 is subunit of the TAK1 MAP3K responsible for TGFβ activation

of MAPKs, they focused on TAB1. TGFβ stimulation of WT but not mPHD ES cells led to increased K63 Ub chains on MEKK1 itself and TAB1, but not the other four candidate substrates. They generated Tab1-/- ES cells and showed that neither EGF nor TGFβ could activate MAPKs in these cells, and went on to map MEKK1-dependent ubiquitylation sites in TAB1 to K294/319/335/350. Expression of WT but not a K294/319/335/350A mutant TAB1 restored EGF and TGFβ activation of TAK1 and p38 MAPK in the Tab1-/- ES cells. Although the growth of the mPHD ES cells was normal, these cells showed an altered gene expression pattern, with some genes upregulated and others downregulated, a few of these changes were confirmed by qPCR. Next they showed that mPHD and Tab1-null ES cells were defective in embryoid body formation and in neuroectodermal and mesodermal differentiation. Finally, they used these ES cells to create mPHD mice, and found that the mPHD/mPHD genotype was embryonic lethal, which is contrast to Mekk1-/- and Mekk1ΔK/Mekk1ΔK mice lacking the catalytic domain where at least some mice survive. The mPHD/+ heterozygous mice exhibited some subtle phenotypes, including cardiac fibrosis, as well as defects in Itch phosphorylation, which is JNK-mediated, in response to anti-CD3/CD28 stimulation of T cells.

These studies provide some of the first evidence for an important biological function for the E3 Ub ligase activity of the RING-like PHD domain of the MEKK1 MAP3K, which shows that the PHD domain is needed for activation of the JNK and p38 MAP kinases downstream of TAK1 in response to activation of the TGFβ and EGF receptors, and that this is mediated through MEKK1 PHD-catalyzed formation of K63 Ub chains on TAB1, an activating subunit of the TAK1 complex.

1. The major unanswered question is what K63 Ub chains on TAB1 do to increase ligand-induced MAPK activation. The authors suggest that TAB1 ubiquitylation potentiates the interaction between TAB1 and TAK1, based on the finding that the quadruple Ala mutation reduces the binding of TAB1 to TAK1. However, while mTAB1 bound TAK1 less efficiently and MAPK was not activated by TGFβ in mTAB1 expressing Tab1-/- ES cells (Figure E4h), it is not certain that these defects were due to a lack of TAB1 ubiquitylation as opposed to the lack of the four Lys positive charges. In this regard, the TAK1 that was coprecipitated with WT TAB1 in Figure E4h seems to be largely in an unmodified state. No direct evidence is provided to show that the ubiquitylated TAB1 species binds TAK1, and this needs to be included. Is it possible that TAK1 interacts directly with K63 Ub chains on TAB1, or is the effect due to ubiquitylation-induced conformational changes in TAB1 promoting its binding to TAK1? In this regard, what domain of TAB1 binds TAK1 and where on TAB1? Alternatively, could K63 Ub chains on TAB1 recruit TAB2/3 via their ZnF domains, and thereby stabilize the ternary complex with TAK1, leading to increased activation in the cell. In this regard, evaluation of whether the TAB2/3 ZnF domains can bind MEKK1-ubiquitylated TAB1 and are needed for TAB1 function in the activation of MAPKs would be informative.

2. Another issue is why mPHD homozygous mice are embryonic lethal, when at least some Mekk1-null and homozygous Mekk1ΔK mice are born. Could this be a result of unregulated MEKK1 activity due to lack of the PHD domain? The discussion of this issue on page 18 needs fleshing out. In this regard, it would have been better if the authors had generated a conditional mPHD mouse designed so that mPHD MEKK1 expression could have been turned on in specific cell types, either inducibly or in a tissue-specific fashion, which could be done either in a WT or a Mekk1-/+ background.

3. In this regard, the authors do not indicate at what day of embryogenesis the mPHD/mPHD mice die, which is important information, particularly because it would be important to test whether the TGFβ/EGF signaling pathway defects observed in the mPHD ES cells are also evident in other cell types, e.g. MEFs (if they can be generated, which depends on when the embryos die).

Points: 1. Tables E2 and E3: It is not clear whether the array proteins that became ubiquitylated in the absence of MEKK1 PHD were subtracted from the list of proteins in Table E3.

2. Figures 3 and E3: How were the TAB1, TRAF2, TNIP1, TNIP2 and STAM1 proteins purified from 293 cells for the ubiquitylation assays shown in Figure E3b-f, i.e. were they tagged, and if so what was the tag, and was the ubiquitylation assay done on the beads or in solution after elution?

© European Molecular Biology Organization

Likewise, for the in vivo ubiquitylation assays in Figure 3d-h, were these proteins immunoprecipitated by their tags or by anti-target protein antibodies?

3. Figure 4: It would help the reader if each of these panels indicated what cytokine was used on the figure. Why was TGFβ stimulation of ERK phosphorylation reduced by the NSC697923 UBE2N E2 and TAK1 inhibitors, i.e. what is pathway is involved that requires ubiquitylation activity? How selective is oxozeaenol as a TAK1 inhibitor? As the authors indicate, TGFβ did not stimulate TRAF2 K63 ubiquitylation in vivo, although MEKK1 could ubiquitylate TRAF2 in vitro in a PHD domain-dependent manner - do they have an explanation?

4. Figures 4 and E4: How was TAK1 purified out of the in vitro ubiquitylation reaction with MEKK1 WT and mPHD, shown in Figure E4i - were these MEKK1 constructs kinase-active and could they have been carried over into the TAK1 kinase assay (can MEKK1 phosphorylate TAB1?)? The details of how this experiment was done are not included in the Methods section. More importantly, what fraction of the input TAB1 was ubiquitylated in these assays - from Figure 4F its looks as though it was <5%, and in Figure E4i there was no change in the level of TAB1 running at the size of the unmodified TAB1 when MEKK1 WT was present. If possible, the fraction of TAB1 that became ubiquitylated in these assays should be quantified. Ideally, the authors should purify the ubiquitylated species of TAB1 and compare the ability of this to activate TAB1 with the activity of unubiquitylated TAB1. In addition, they need to carry out this experiment with the non-ubiquitylatable mutant of TAB1.

5. Figures 5 and E5: What were the phospho-MAPK levels in WT versus mPHD ES cells like under these growth conditions? Is LIF activation of MAPKs reduced in mPHD ES cells? What about the gene expression patterns of WT and mPHD ES cells in response to TGFβ? It would be very helpful if in addition to the color coding, each of the bars in histograms were labeled with the cell type and treatment time/condition on the figure, rather than forcing the reader to read and re-read the legend!

6. Figure 7: Is the smaller teratoma size with mPHD ES cells due to a failure of the cells to proliferate or survive, or a result of premature differentiation?

7. The title of the paper is a little overstated, since the consequences of mutation so the MEKK1 PHD domain on ES differentiation is a minor part of the paper, and not fooled up in depth. It might be better to mention its role in MAPK pathway activation in response to TGFβ and EGF.

1st Revision - authors' response 23 June 2014

Referee #1: *"Although the topic is very interesting, the current version of this manuscript is immature and several points should be addressed before publication in the EMBO Journal."*

We would thank the referee for their encouraging comments about our work.

Major points
1. There are too many 'unpublished data' in this manuscript (P9 L24, P10 L22, P13 L5, P13 L8, P13 L20, P13 L23, P14 L21, P15 L11). All 'unpublished data' should be displayed.

We have now incorporated a large amount of the unpublished data listed above and also new findings into the manuscript.

2. The role of MEKK1 kinase activity for MAPK signaling in ES cells is not clear. Although the authors examine the kinase activity of MEKK1 mPHD mutant in HEK293 cells (Fig 1b), MEKK1 kinase activity in ES cells is not examined. Is MEKK1 kinase activity involved in EGF or TGF-beta-induced MAPK signaling in ES cells? Also, does MEKK1 C438A, I440A double mutant have the kinase activity?

The papers by Xia *et al*. (PNAS, 2000) and Yujiri *et al*. (Science, 1998) have looked extensively at the role of MEKK1 kinase signaling in response to agonists using ES cells. Here our work instead

© European Molecular Biology Organization

focuses on the PHD motif, which is much less well understood. We have now included kinase assay quantitation of the WT and MEKK1 C438A, I440A mutant in Fig E1A to further improve our results, and this is in concordance with the findings reported by Lu *et al*. (Mol Cell, 2002).

3. Although the authors argue that microarray data revealed normal expression of TGF-beta receptors and EGF family receptors in Map3k1mPHD ES cells, microarray data are not convincing to persuade the expression level of those receptors. Also, the authors should describe the expression level of those receptors in TAB1-/- ES cells.

Our ES cell bioinformatics data in the original manuscript shows gene hits with a difference of expression of 2-fold or more between *WT* and $Map3k1^{mPHD}$ ES cells. This means that the genes the reviewer is requesting information on outside of this analysis have small and insignificant differences between *WT* and $Map3k1^{mPHD}$ data sets. As a control we have now shown *TGFβR* gene expression analysis for *WT* and $Map3k1^{mPHD}$ ES cells in Fig E7A and B. *EGFR* gene expression similarly shows no significant differences between *WT* and $Map3k1^{mPHD}$ ES cells, but we have left their analysis out of the expanded view figure as the focus of the mechanism looks at TGF-β signaling and the data adds little, since it is a negative result. Our global gene expression analysis is also available at ArrayExpress (accession: E-MTAB-1679).

4. In Fig 4a and Fig 4b, is TAK1 activation inhibited by those inhibitors?

TAK1 activation is ablated by the UBE2N and TGFβR inhibitors (Fig 5B).

Minor points
1. In Fig E4, immunoblot of TAB1 should be shown.

Fig E4D shows expression of TAB1 in *WT and* $Tab1^{-/-}$ ES cells by immunoblot.

2. In Fig E7b, the result is presented without quantification. Quantification of the data from different experiments should be shown.

We have added quantitation for this result (Fig 9B).

3. In Fig 6a, Fig E7c and Fig E7d, scale bars should be present. Also, molecular weight markers should be shown in western blot data.

We have added-in scale bars to improve the presentation of our microscopy data. Our Western data uses previously characterized antibodies (listed in the Materials and methods section with catalogue numbers or references) so we have not added this detail as we think it adds little to the manuscript figures.

4. In Fig E7d, the figure legend does not help much. What does the arrow indicate?

We have now rewritten the legend for this data figure. The arrows in the figure indicate the Leydig cells within the testis.

Referee #2: *"Overall, the study is very novel and revealed a novel substrate for MEKK1. The characterization of the role of MEKK1 PHD motif in ES cell culture, mouse development and tumorigenesis was well executed. There are some minor issues needs to be resolved before it can be published in EMBOJ"*

We would thank the reviewer for their positive critique of our manuscript.

1. In vitro ubiquitination assay in Fig E3b-f: The author should clearly describe or label which ubiquitination signal is from the substrates (e.g. TAB1 and others), but not from MEKK1 autoubiquitination.

We have amended the manuscript text to address this detail.

2. The interaction between MEKK1 and TAB1 was shown under overexpression conditions (Fig E4b). The authors may want to prove this notion under the physiological conditions.

An endogenous immunoprecipitation experiment has been added in Fig E4I.

3. TAB1, but not mTAB1, restoration rescued the Mash1 level in TAB1 deficient ES cells in Fig. 6f. How about the levels of other defective genes, such as 4j.Nestin, Pax6?

We have now added in the *Nestin* and *Pax6* real time PCR data into the manuscript (Fig E9).

4. To study the function of mutant PHD Map3k1 in ES cells, mutant PHD Map3k1 knockin ES cells have been generated. Are these cells homozygous? Since ES cell targeting usually generates heterozygous cells, it is encouraging to describe how homozygous ES cells are generated. The author should also provide the information about how to identify homozygous knockin ES cells. In Fig1d (Southern blotting), there is no clear labeling indicating which allele corresponds to which band.

For the *Map3k1* targeting we first generated $Map3k1^{mPHD/+}$ ES cell clones and then further selected positive clones for the loss of both *WT* alleles to generate the homozygous $Map3k1^{mPHD}$ clones. The selection of $Map3k1^{mPHD}$ clones was made by Southern blotting and genomic PCR of their DNA. The procedure for generating mutant *Map3k1* clones is previously described in Xia *et al*. (PNAS, 2000), and we have indicated this by reference in the manuscript. We have also amended Fig 1D to show the mutant *Map3k1* allele.

Referee #3: *"These studies provide some of the first evidence for an important biological function for the E3 Ub ligase activity of the RING-like PHD domain of the MEKK1 MAP3K, which shows that the PHD domain is needed for activation of the JNK and p38 MAP kinases downstream of TAK1 in response to activation of the TGFβ and EGF receptors, and that this is mediated through MEKK1 PHD-catalyzed formation of K63 Ub chains on TAB1, an activating subunit of the TAK1 complex."*

We would like to thank the reviewer for their critique and suggestions that have proven to be very useful in improving our work further.

1. The major unanswered question is what K63 Ub chains on TAB1 do to increase ligand-induced MAPK activation. The authors suggest that TAB1 ubiquitylation potentiates the interaction between TAB1 and TAK1, based on the finding that the quadruple Ala mutation reduces the binding of TAB1 to TAK1. However, while mTAB1 bound TAK1 less efficiently and MAPK was not activated by TGFβ in mTAB1 expressing Tab1-/- ES cells (Figure E4h), it is not certain that these defects were due to a lack of TAB1 ubiquitylation as opposed to the lack of the four Lys positive charges. In this regard, the TAK1 that was coprecipitated with WT TAB1 in Figure E4h seems to be largely in an unmodified state. No direct evidence is provided to show that the ubiquitylated TAB1 species binds TAK1, and this needs to be included. Is it possible that TAK1 interacts directly with K63 Ub chains on TAB1, or is the effect due to ubiquitylation-induced conformational changes in TAB1 promoting its binding to TAK1? In this regard, what domain of TAB1 binds TAK1 and where on TAB1? Alternatively, could K63 Ub chains on TAB1 recruit TAB2/3 via their ZnF domains, and thereby stabilize the ternary complex with TAK1, leading to increased activation in the cell. In this regard, evaluation of whether the TAB2/3 ZnF domains can bind MEKK1-ubiquitylated TAB1 and are needed for TAB1 function in the activation of MAPKs would be informative.

Ono *et al*. (JBC, 2001) have shown using a sensitive yeast-two hybrid assay that a small C-terminal fragment of TAB1 (480–495) can interact with TAK1. In our HEK 293 cell assays the binding and ubiquitination of TAB1 by the MEKK1 PHD is likely an initiating step in MAPK pathway activation, and represents a fraction of the total protein when overexpressed in HEK 293 cells. Indeed, in our *in vitro* analysis there are several deubiquitinating peptidases that can remove MEKK1 PHD ubiquitination very efficiently (Fig E1D).

To best detect ubiquitinated TAB1 in HEK 293 cell assays we have used HA tagged Ub, e.g. Fig E4A and E4G. We would propose a model whereby TAB1 binding and ubiquitination by MEKK1 leads to conformational changes within the proteins that assists TAK1 binding to the

MEKK1:ubiquitinated TAB1 complex in cells. We have now shown the small TAK1 binding domain on the full-length TAB1 schematic in Fig E4F.

To address whether TAB2 is also be recruited to the MEKK1:ubiquintated TAB1 complex we have used IP experiments to show that TAB2, but not TAB2 lacking the ZnF motif, is recruited to TAB1 ubiquitinated by the MEKK1 PHD (Fig 5A). This finding supports the results of Kanayama *et al.* (Mol Cell, 2004) that suggest the ZnF motif of TAB2 can bind ubiquitinated proteins. We have also added a schematic model of the MEKK1:TAB signaling complex based on our data set (Fig 5C).

2. Another issue is why mPHD homozygous mice are embryonic lethal, when at least some Mekk1-null and homozygous Mekk1ΔK mice are born. Could this be a result of unregulated MEKK1 activity due to lack of the PHD domain? The discussion of this issue on page 18 needs fleshing out. In this regard, it would have been better if the authors had generated a conditional mPHD mouse designed so that mPHD MEKK1 expression could have been turned on in specific cell types, either inducibly or in a tissue-specific fashion, which could be done either in a WT or a Mekk1-/+ background.

The change from partial lethality to early embryonic lethality between $Map3k1^{\Delta KD}$ and $Map3k1^{mPHD}$ genotypes likely reflects the fact that as well as altering MAPK activation from cytokine receptors the Ub-proteasome system also becomes imbalanced (Fig E2B and C), resulting in a more significant phenotype in mice. The rationale for germline mutation of *Map3k1* was to allow comparison with the $Map3k1^{\Delta KD}$ mice and conditional knockin approaches would be the next stage for the research. By analyzing a germline mutant of MEKK1 our work is comparable to the previous analyses of Xia *et al*. (PNAS, 2000) and Yujiri *et al*. (Science, 1998).

3. In this regard, the authors do not indicate at what day of embryogenesis the mPHD/mPHD mice die, which is important information, particularly because it would be important to test whether the TGFβ/EGF signaling pathway defects observed in the mPHD ES cells are also evident in other cell types, e.g. MEFs (if they can be generated, which depends on when the embryos die).

We report early embryonic lethality, with $Map3k1^{mPHD}$ embryos failing to progress beyond a few days at most, and at too early a stage for the production and analysis of MEFs. An extension of this work would be to differentiate the $Map3k1^{mPHD}$ ES cells into other cell types, e.g. cardiac myocytes, as was done by Minamino *et al*. (PNAS, 1999) with $Mekk1^{-/-}$ ES cells.

Points:
1. Tables E2 and E3: It is not clear whether the array proteins that became ubiquitylated in the absence of MEKK1 PHD were subtracted from the list of proteins in Table E3.

No screen hits have been subtracted from either data set table and the tables display the raw Z scores from the two protein array screens.

2. Figures 3 and E3: How were the TAB1, TRAF2, TNIP1, TNIP2 and STAM1 proteins purified from 293 cells for the ubiquitylation assays shown in Figure E3b-f, i.e. were they tagged, and if so what was the tag, and was the ubiquitylation assay done on the beads or in solution after elution? Likewise, for the in vivo ubiquitylation assays in Figure 3d-h, were these proteins immunoprecipitated by their tags or by anti-target protein antibodies?

The recombinant proteins listed above were immunopurified using antibodies specific for a tag or the protein of interest itself, and HiTrap Protein G columns were used for the purification step. After extensive washing the proteins of interest were then eluted for further assays.

3. Figure 4: It would help the reader if each of these panels indicated what cytokine was used on the figure. Why was TGFβ stimulation of ERK phosphorylation reduced by the NSC697923 UBE2N E2 and TAK1 inhibitors, i.e. what is pathway is involved that requires ubiquitylation activity? How selective is oxozeaenol as a TAK1 inhibitor? As the authors indicate, TGFβ did not stimulate TRAF2 K63 ubiquitylation in vivo, although MEKK1 could ubiquitylate TRAF2 in vitro in a PHD domain-dependent manner - do they have an explanation?

We have changed the figure panels to show the cytokines used in Fig 4 and E4. In a system-specific manner TAK1 inhibition or *Map3k7* genetic ablation can lead to deficient ERK activation, e.g. Ear *et al*. (Journal of Immunology, 2014) and Shim *et al*. (EMBOJ, 2009). The TAK1 inhibitor (5Z)-7-Oxozeaenol has an IC_{50} of 8 nM and displays more than 33-fold selectivity over MEKK1, and in our *in vitro* short-term stimulation assays of ES cells is a reasonable means for assessing the role of TAK1 kinase activity. Yamashita *et al*. (Mol Cell, 2008) have shown that MAPK activation induced by TGFβR can be ablated by a TRAF6 deletion mutant lacking the RING domain, but not by a similar TRAF2 mutant. Thus, while TRAF6 is critical for TGFβR signaling TRAF2 is not, and we would suggest this is why TRAF2 is not ubiquitinated following TGF-β stimulation in our hands.

4. Figures 4 and E4: How was TAK1 purified out of the in vitro ubiquitylation reaction with MEKK1 WT and mPHD, shown in Figure E4i - were these MEKK1 constructs kinase-active and could they have been carried over into the TAK1 kinase assay (can MEKK1 phosphorylate TAB1?)? The details of how this experiment was done are not included in the Methods section. More importantly, what fraction of the input TAB1 was ubiquitylated in these assays - from Figure 4F its looks as though it was <5%, and in Figure E4i there was no change in the level of TAB1 running at the size of the unmodified TAB1 when MEKK1 WT was present. If possible, the fraction of TAB1 that became ubiquitylated in these assays should be quantified. Ideally, the authors should purify the ubiquitylated species of TAB1 and compare the ability of this to activate TAB1 with the activity of unubiquitylated TAB1. In addition, they need to carry out this experiment with the non-ubiquitylatable mutant of TAB1.

We have further clarified the data description within the manuscript text, created a schematic of the assays involved (Fig E5A) and also moved and expanded this figure from its original compact form in Fig E4I into a more detailed form in Fig E5B. The Western blots of the input proteins are now more clearly labeled and the Lys63-linked Ub Western blot of the *in vitro* ubiquitination assay step has been included as a control. Non-ubiquitinated TAB1 is present in lanes 3 and 5, where lane 5 used a defective PHD mutant of MEKK1 as a control. Lane 4 shows MEKK1 PHD ubiquitinated TAB1, and this is purified for the *in vitro* TAK1 kinase assay. MEKK1 with a defective mutant PHD, and as shown in Fig 1 and E1 still possesses an active kinase domain, shows that MEKK1 kinase domain phosphorylation does not account for the enhanced TAK1 activation in the assay (lanes 4 and 5). Finally, using TAB1 and mTAB1 proteins in the assays we show that mutation of the lysines in TAB1 ubiquitinated by the MEKK1 PHD ablates TAK1 activation by TAB1 (Fig E5C).

5. Figures 5 and E5: What were the phospho-MAPK levels in WT versus mPHD ES cells like under these growth conditions? Is LIF activation of MAPKs reduced in mPHD ES cells? What about the gene expression patterns of WT and mPHD ES cells in response to TGFβ? It would be very helpful if in addition to the color coding, each of the bars in histograms were labeled with the cell type and treatment time/condition on the figure, rather than forcing the reader to read and re-read the legend!

The data in question (now Fig 6 and E6) examines global gene expression defects in pluripotent *Map3k1*^{mPHD} ES cells where LIF is used to maintain pluripotency. Under these conditions MAPK activation is at a basal state making it more difficult to detect by phospho-MAPK antibodies than by starving and stimulating ES cells with an agonist such as TGF-β. Under these conditions we can detect no significant difference in MEKK1 kinase activity between *WT*, *Map3k1*^{mPHD} and *Tab1*$^{-/-}$ ES cells (Fig E7C).

Our global gene expression analysis approach identified numerous genes that were aberrantly expressed in *Map3k1*^{mPHD} ES cells (e.g. *Acta1*, *Ddx3Y*, *Dusp4*, *Dusp14*, *Nnat*, *Otx2*, *Tec*, *Nes*, *Nuak1*, *Runx1* and *Tagin*), but there is no significant defect in the pluripotency or proliferation of *Map3k1*^{mPHD} ES cells. Fig 7 then looks at ES cell *in vitro* differentiation, but here the critical gene expression markers are already well established making the need for TGF-β global gene expression analysis in ES cells unnecessary, and we do after all use a TGFβR inhibitor in our analysis. We have added further labels to Fig E6 to improve the clarity of the data. In addition, we have added further bioinformatics analysis of TGFβR expression between *WT* and *Map3k1*^{mPHD} ES cells into the manuscript in Fig E7.

6. Figure 7: Is the smaller teratoma size with mPHD ES cells due to a failure of the cells to proliferate or survive, or a result of premature differentiation?

We have examined the tumors by histology and note an absence of cartilage-like tissue in the tumors formed by *Map3k1*^{mPHD} and *Tab1*$^{-/-}$ ES cells. This suggests significantly skewed ES cell differentiation and tissue formation within the teratomas (Fig 8E).

7. The title of the paper is a little overstated, since the consequences of mutation so the MEKK1 PHD domain on ES differentiation is a minor part of the paper, and not fooled up in depth. It might be better to mention its role in MAPK pathway activation in response to TGFβ and EGF. Re: EMBOJ-2014-88351 The MEKK1 PHD ubiquitinates TAB1 to control stem cell differentiation and tumorigenesis.

We agree with the reviewer and have now changed the title of our work to "The MEKK1 PHD ubiquitinates TAB1 to activate MAPKs in response to cytokines".

2nd Editorial Decision 18 July 2014

Thank you for submitting your revised manuscript. I appreciate the progress you made in your revision, and I am pleased to see that the referees' remarks are overall positive. Your manuscript has now been reviewed once more by the original referees. Two reviewers fully endorse now its publication. Referee #3 acknowledges that you have, in part, addressed critical concerns (i.e. relevant to this reviewer's request to provide additional insights into how the K63 chains on TAB1 could possibly enhance TAK1 activation (via Znf domain-dependent recruitment of TAB2) as well as have expanded on the TAK1 vitro kinase assay testing the impact of ubiquitylated Tab1. Reviewer #3 acknowledges that the presented data are consistent with your model, but does retain that the additional data have not fully conclusively and formally provided evidence that the species of TAB1 associated with TAB2 and TAK1 was ubiquitylated, or that the species of TAB1 stimulating TAK1 activity was indeed the ubiquitylated TAB1 species.

We are inviting you to address these two points raised by referee #3 - At the same time, after further consultation with reviewers, and given the multiple lines of evidence in support for the key conclusions in the presented dataset, the editorial team thinks that acknowledging the experimental caveats raised and responding to them by discussing them carefully in the form of textual changes in the manuscript would also be sufficient for acceptance of your manuscript at this point, without the need for generating additional experimental data.

Together, before we formally accept your manuscript for publication, we would like to invite you to attend to this final touch-up. I am thus returning the study to you for a final revision step, hoping you will be able to re-submit a final version of the manuscript as early as possible. Please also submit a document with a point-to-point response to referee #3 - we should then be in a position to accept the manuscript and swiftly proceed with its production for publication in The EMBO Journal. Should you have any further questions in this regard, please get back to me.

Referee #1:

In this revised version, the authors have addressed my main concerns and I have no further points.

Referee #2:

The revision has addressed our major concerns. It is suitable to be published.

Referee #3:

In response to the reviews the authors have added some new experimental data, and have included some data previously not shown as supplementary figures.
While in general this is now acceptable for EMBO Journal, the issue of how TAB1 ubiquitylation promotes TAK1 activation is still not fully resolved. In the new data in Figure 5A, they showed that association of TAB2 with TAB1 in cells expressing MEKK1 depended on the TAB2 NZF Ub-binding domain. However, they did not show that the species of TAB1 associated with TAB2 were in fact ubiquitylated. Moreover, this experiment lacks a key control with PHD mutant MEKK1, which is required to show that the observed association of TAB2 with TAB1 was dependent on MEKK1 E3 ligase activity. What they really need to do is to demonstrate that WT but not ΔZnF TAB2 brings down ubiquitylated (i.e. slower migrating species) of TAB1 from cells expressing WT but not mPHD MEKK1.
Admittedly, one would only expect a small fraction of the TAB1 population to be ubiquitylated, but unless TAK1 binds to ubiquitylated TAB1 (possibly itself bound to TAB2), and remains bound when TAB1 is deubiquitylated, one would expect to be able to show that TAK1 molecules bound to TAB1 are ubiquitylated. Moreover, if TAB2 does associate with TAB1 via its K63 Ub chains, then these chains should be protected from deubiquitylation by the NZF domain, and then one would expect to be able to detect ubiquitylated TAB1 in association with TAK1, as their model In Figure 5C posits. For this purpose, they need to immunoprecipitate TAK1, from cells co-expressing TAK1, TAB1 and TAB2, and show that ubiquitylated species of TAB1 are brought down with TAK1. In the same vein, the new Figure E5A/B shows that ubiquitylation of TAB1 increases TAK1 activity, but, since most of the TAB1 coming from the MEKK1 ubiquitylation step was not ubiquitylated, these data do not prove that the ubiquitylated TAB1 species per se are the TAB1 molecules responsible for stimulating TAK1 activity in the second step (also no exogenous TAB2 was included in this assay, and it is not clear whether any endogenous TAB2 was present in association with the ubiquitylated TAB1. Ideally, what the authors need to do is to identify the sites of ubiquitylation in TAB1 and then show that a non-ubiquitylatable mutant TAB1 does not support TAK1 activation by MEKK.
Point: 1. Based on the data in Figure E7C, the authors claim that the level of MEKK1 kinase activity was the same in Map3k1mPHD and Tab1-/- ES cells, but it actually looks as though the levels of pMEKK1 were significantly lower in these two cells compared to WT ES cells

2nd Revision - authors' response 13 August 2014

For the third reviewer comments we have the following point-by-point response.

"While in general this is now acceptable for EMBO Journal, the issue of how TAB1 ubiquitylation promotes TAK1 activation is still not fully resolved. In the new data in Figure 5A, they showed that association of TAB2 with TAB1 in cells expressing MEKK1 depended on the TAB2 NZF Ub-binding domain. However, they did not show that the species of TAB1 associated with TAB2 were in fact ubiquitylated. Moreover, this experiment lacks a key control with PHD mutant MEKK1, which is required to show that the observed association of TAB2 with TAB1 was dependent on MEKK1 E3 ligase activity. What they really need to do is to demonstrate that WT but not ΔZnF TAB2 brings down ubiquitylated (i.e. slower migrating species) of TAB1 from cells expressing WT but not mPHD MEKK1. Admittedly, one would only expect a small fraction of the TAB1 population to be ubiquitylated, but unless TAK1 binds to ubiquitylated TAB1 (possibly itself bound to TAB2), and remains bound when TAB1 is deubiquitylated, one would expect to be able to show that TAK1 molecules bound to TAB1 are ubiquitylated. Moreover, if TAB2 does associate with TAB1 via its K63 Ub chains, then these chains should be protected from deubiquitylation by the NZF domain, and then one would expect to be able to detect ubiquitylated TAB1 in association with TAK1, as their model In Figure 5C posits. For this purpose, they need to immunoprecipitate TAK1, from cells co-expressing TAK1, TAB1 and TAB2, and show that ubiquitylated species of TAB1 are brought down with TAK1. In the same vein, the new Figure E5A/B shows that ubiquitylation of TAB1 increases TAK1 activity, but, since most of the TAB1 coming from the MEKK1 ubiquitylation step was not ubiquitylated, these data do not prove that the ubiquitylated. TAB1 species per se are the TAB1 molecules responsible for stimulating TAK1 activity in the second step (also no exogenous

TAB2 was included in this assay, and it is not clear whether any endogenous TAB2 was present in association with the ubiquitylated TAB1."

In our protein array screening (Fig. 3) we identified TAB1 as a substrate for the MEKK1 PHD motif, but it is also important to point out that in this high throughput screening assay that TAB2 and TAB3 are not substrates for ubiquitination mediated by the MEKK1 PHD. In Fig. 5 TAB1 is ubiquitinated by overexpression of the MEKK1 PHD in cells and the ubiquitin (Ub) -modified TAB1 population detected via its HA tag. Deletion of the TAB2 zinc finger (ZnF) region, known to bind ubiquitinated proteins, is sufficient to prevent the association of TAB2 with TAB1 under these conditions of overexpression in cells (Kanayama et al., Mol. Cell 2004). We have clarified these points further in our manuscript.

The focus of our manuscript is analyzing how the MEKK1 PHD regulates MAPK signaling in ES cells from cytokine receptors by direct substrate modification with Ub. TAB1 alone is known to be sufficient to activate TAK1 when overexpressed in cells (Shibuya et al., Science 1996). The MEKK1 PHD motif ubiquitinates TAB1 with Lys63-linked poly-Ub and ubiquitinated TAB1 enhances TAK1 activation. Thus, while TAB2 is a known TAK1 binding partner and in our assays it can be recruited to the MEKK1:TAB1 complex, it is a downstream scaffolding event from the MEKK1 PHD motif, since TAB2 is not an actual substrate for MEKK1 PHD ubiquitination. We have amended our manuscript with regards this aspect of our results.

"Ideally, what the authors need to do is to identify the sites of ubiquitylation in TAB1 and then show that a non-ubiquitylatable mutant TAB1 does not support TAK1 activation by MEKK."

We have performed this analysis in Fig. 4 by deletion fragment mapping of the TAB1 region ubiquitinated by the MEKK1 PHD motif, and then point mutation of the lysines within this region of TAB1. Mutation of the lysines to alanine within the TAB1 ubiquitination region will reduce TAK1 activation. It has also previously been published that deletion of the TAB1 c-terminal 68 residues will ablate TAK1 activation (Ono *et al*., JBC 2001). We have refined our manuscript with regards these points.

"Point: 1. Based on the data in Figure E7C, the authors claim that the level of MEKK1 kinase activity was the same in Map3k1mPHD and Tab1-/- ES cells, but it actually looks as though the levels of pMEKK1 were significantly lower in these two cells compared to WT ES cells."

We agree with the reviewer that when continuously growing on serum and in the presence of LIF that endogenous phospho-MEKK1 is slightly reduced in *Map3k1*^{mPHD} and *Tab1*$^{-/-}$ ES cells and we have amended our manuscript text accordingly.
