# Supplementary figures and images for: The MEKK1 PHD ubiquitinates TAB1 to activate MAPKs in response to cytokines
Source: EMBO J. 2014 Sep 26;33(21):2581–96. doi: 10.15252/embj.201488351 (PMC4282369; doi:10.15252/embj.201488351)

## MG132

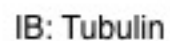

Supplement: Supplementary file 2 [file embj0033-2581-sd2.pdf]

TGF- $\beta$

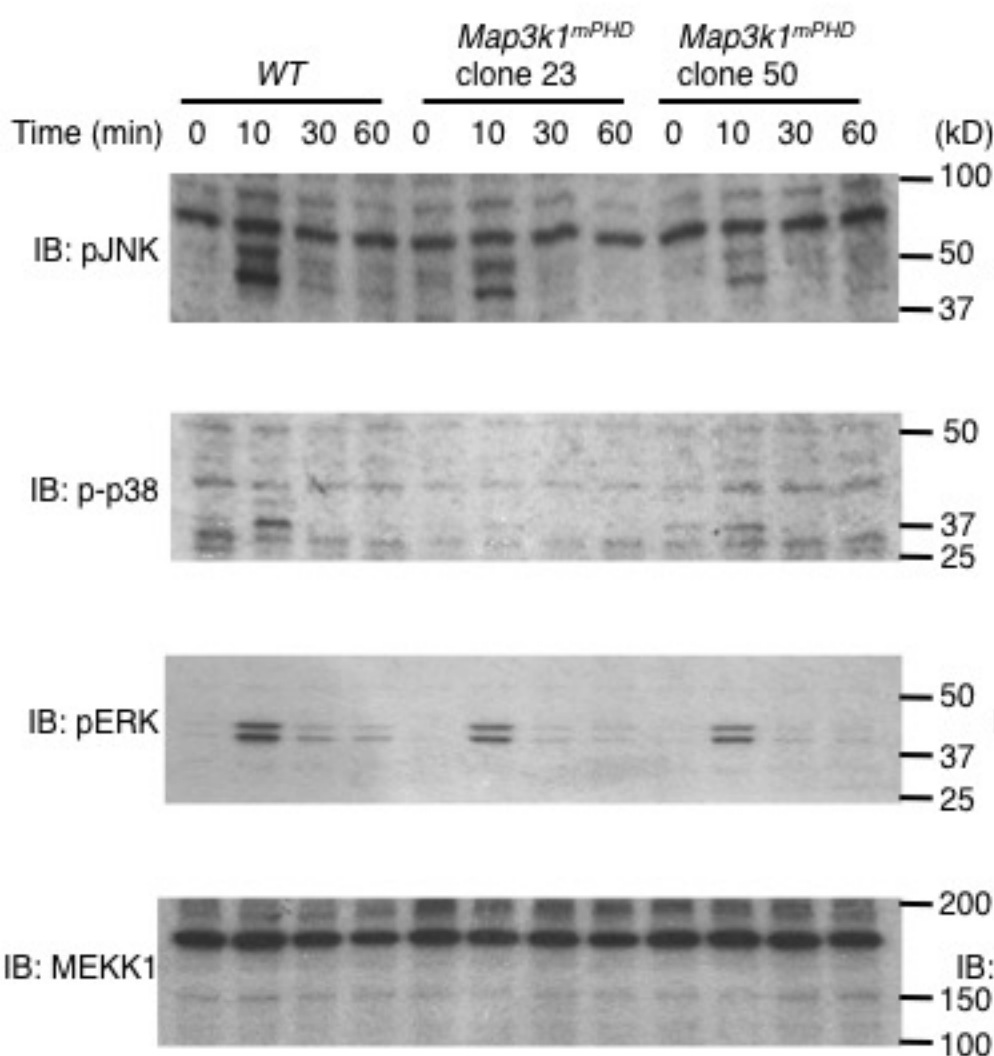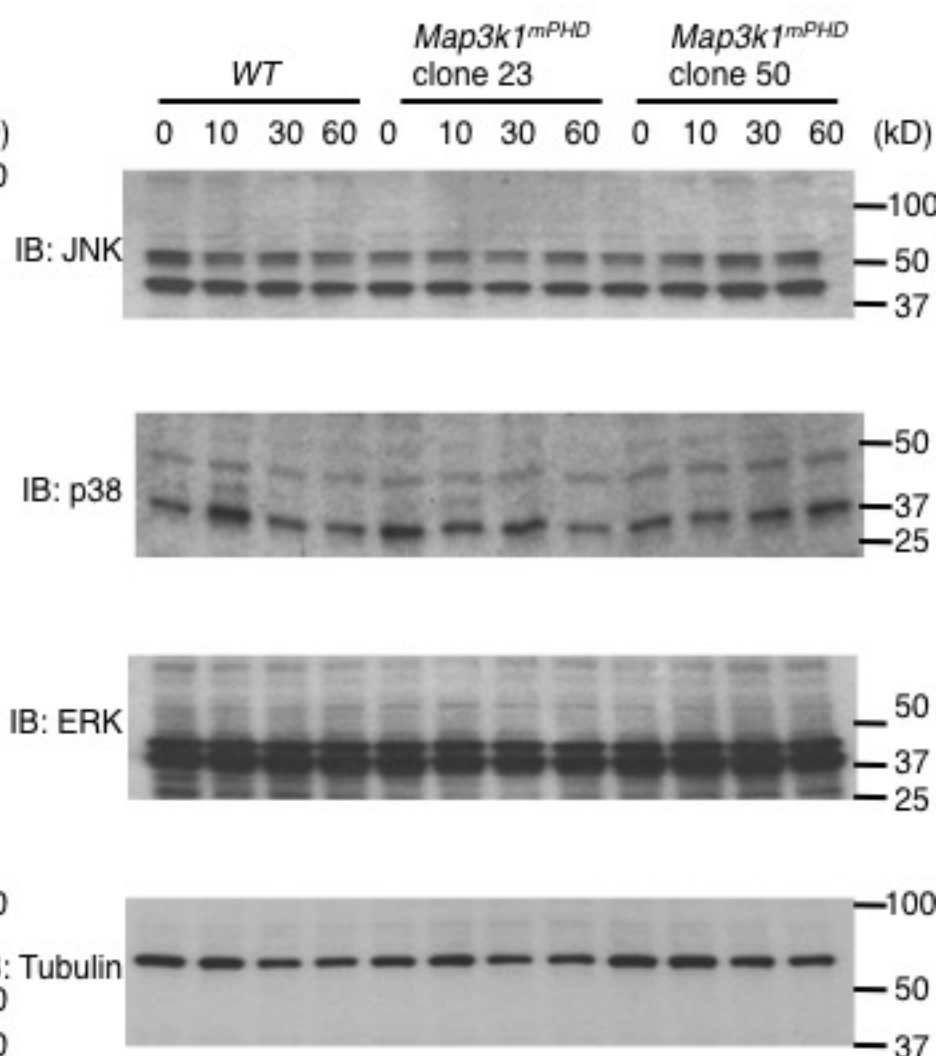

Supplement: Supplementary file 3 [file embj0033-2581-sd3.pdf]

TGF- $\beta$

Additional data 4

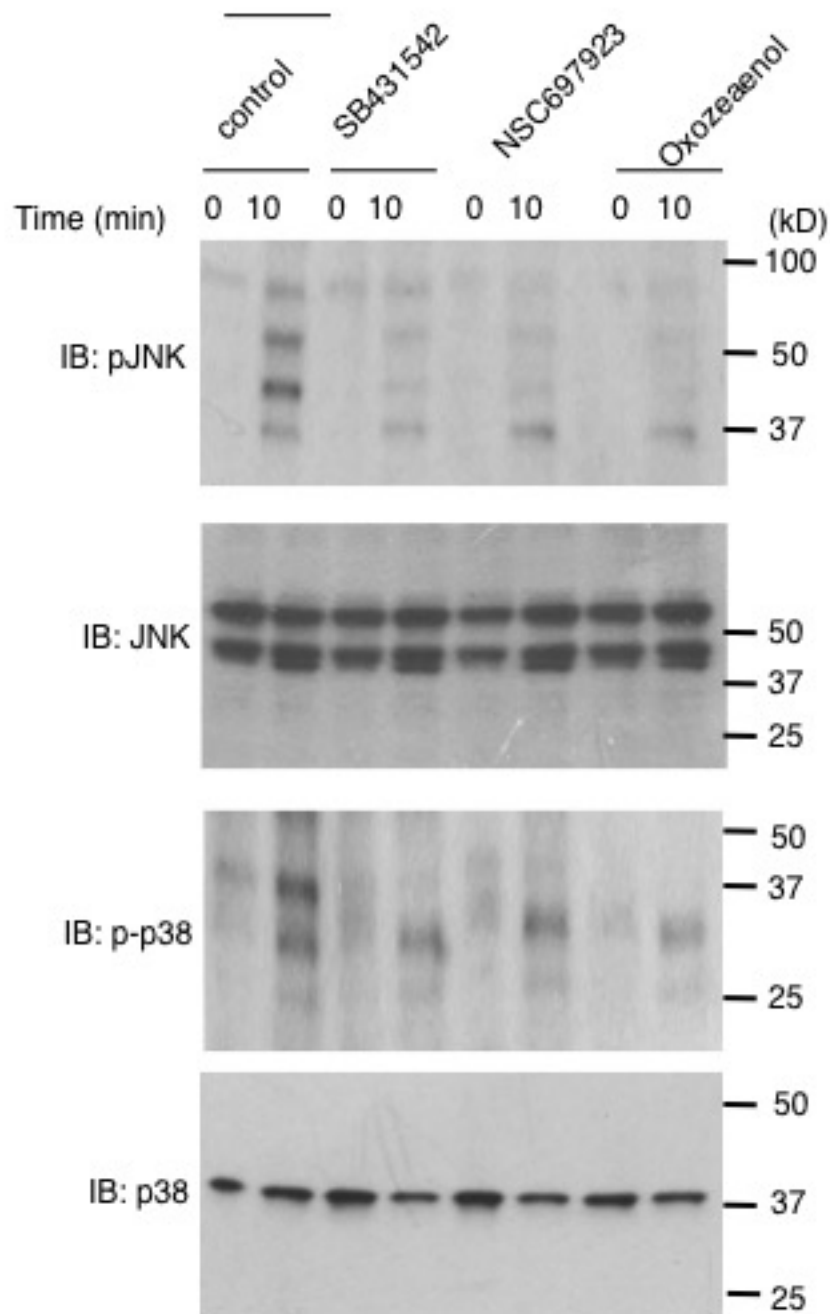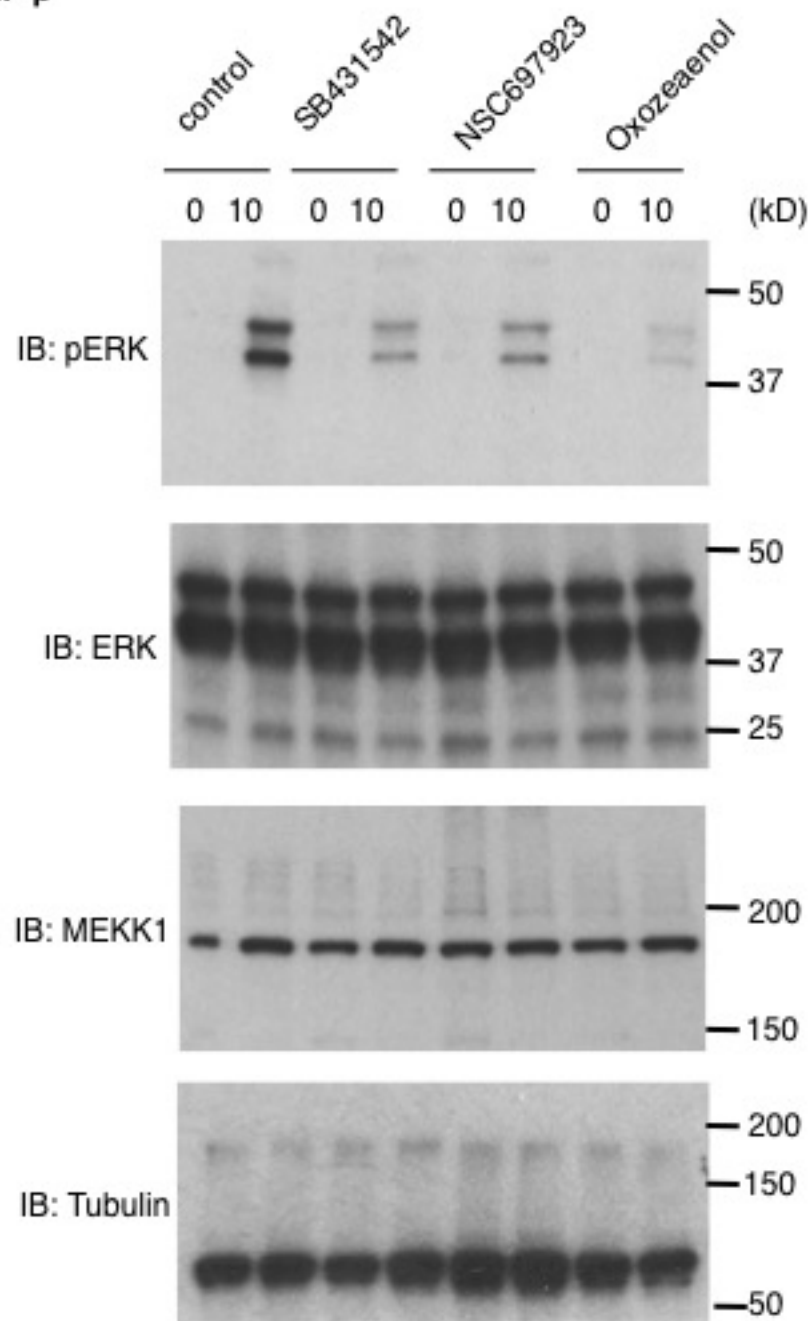

Supplement: Supplementary file 4 [file embj0033-2581-sd4.pdf]

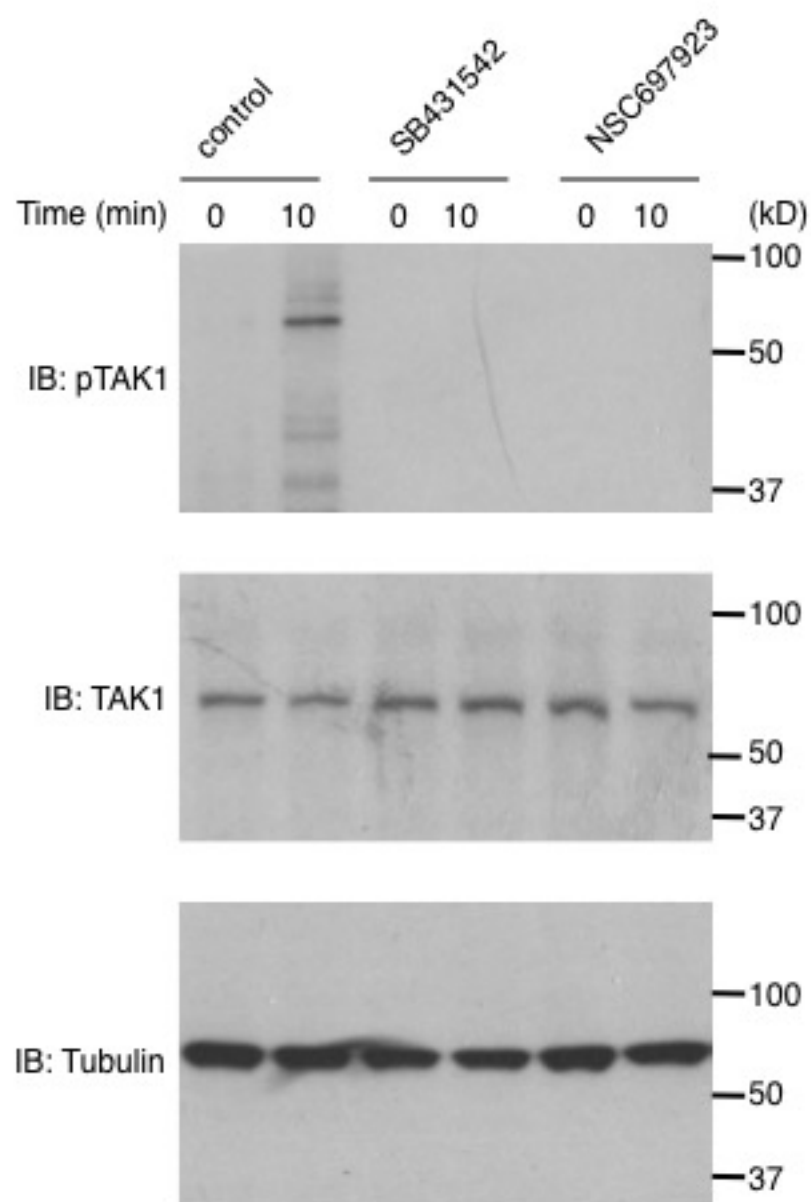

Supplement: Supplementary file 6 [file embj0033-2581-sd6.pdf]

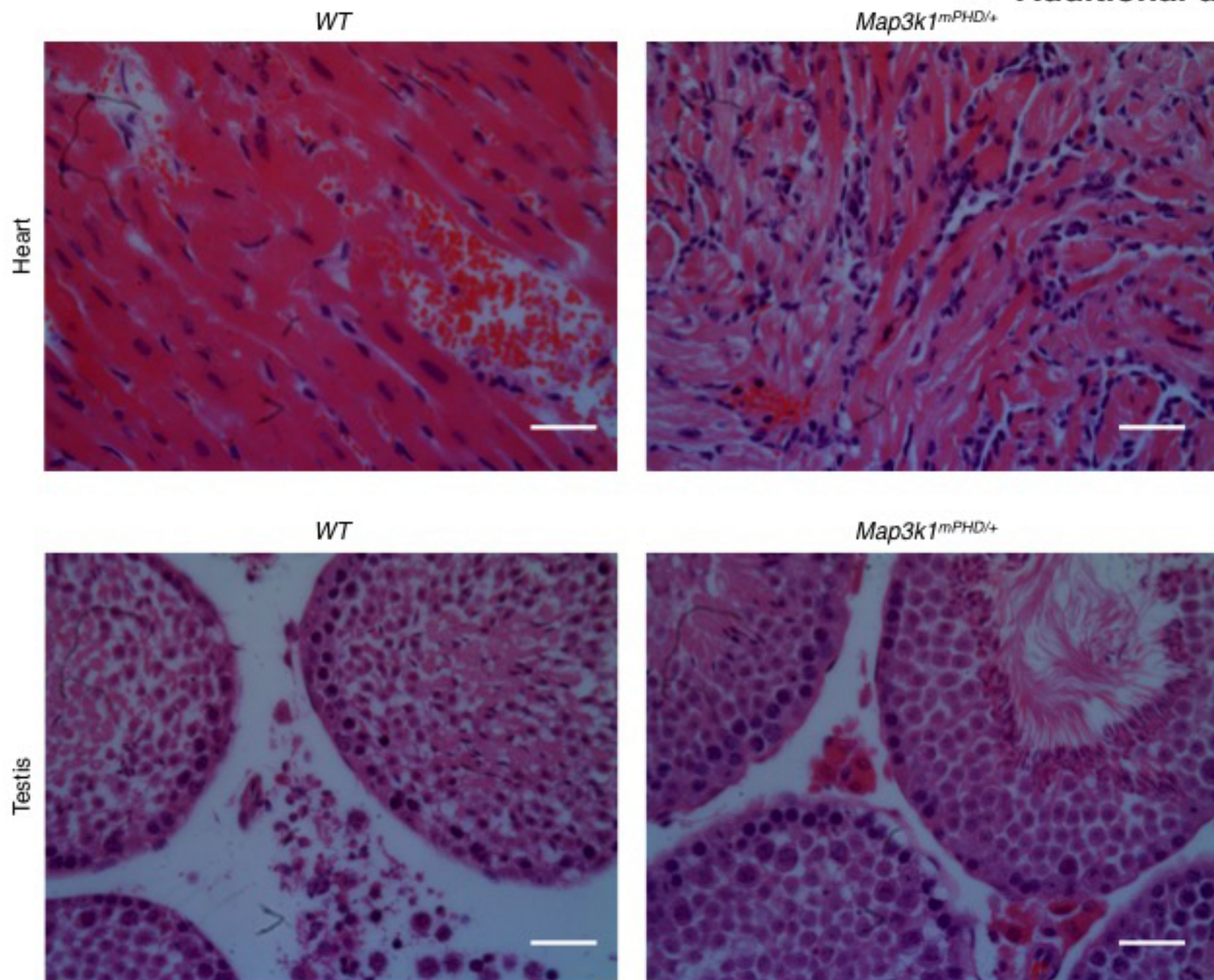

Supplement: Supplementary file 10 [file embj0033-2581-sd10.pdf]
