# Supplementary material for: The MEKK1 PHD ubiquitinates TAB1 to activate MAPKs in response to cytokines
Source: EMBO J. 2014 Sep 26;33(21):2581–96. doi: 10.15252/embj.201488351 (PMC4282369; doi:10.15252/embj.201488351)

Additional data 8

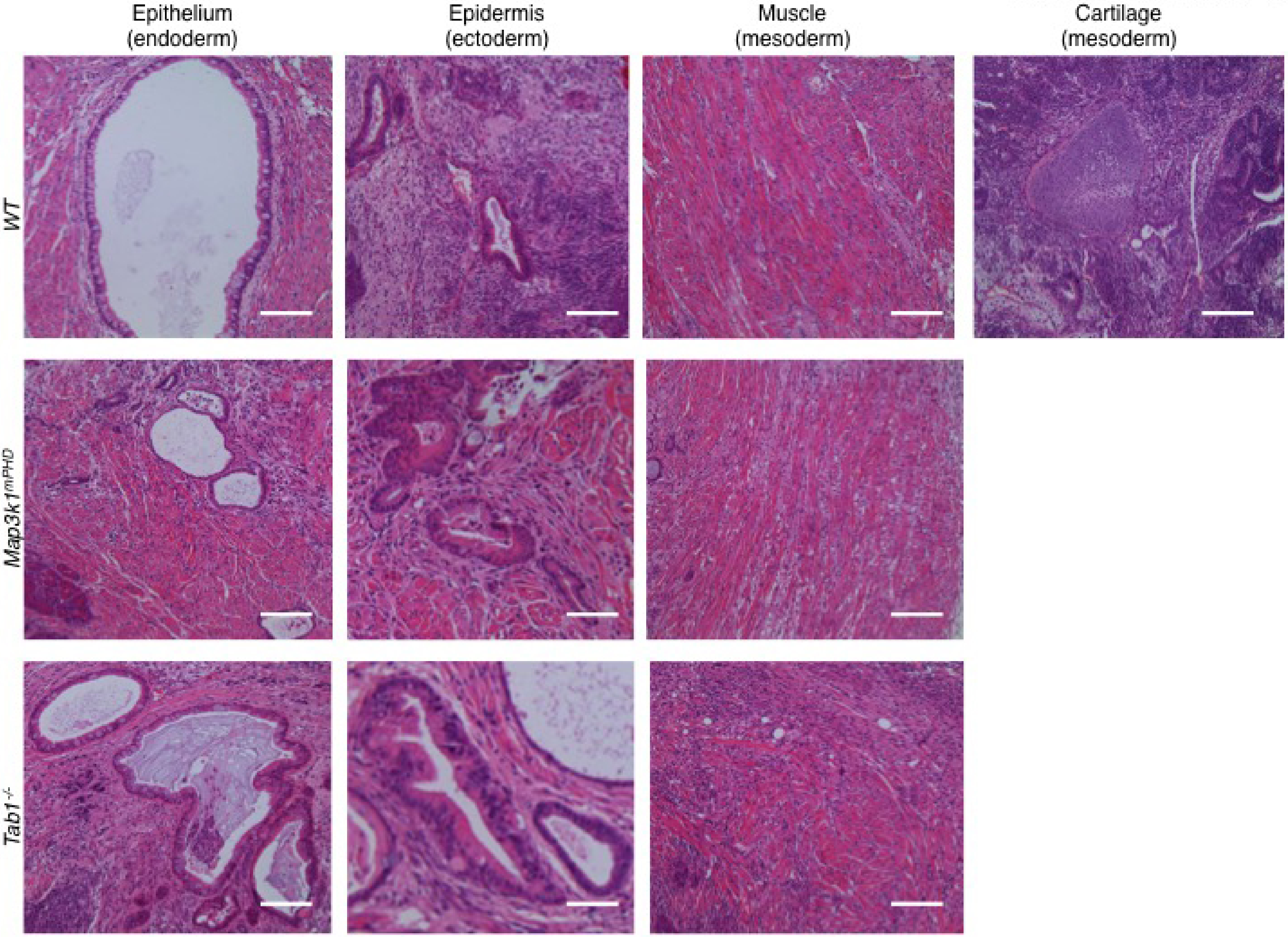

Supplement: Supplementary file 7 [file embj0033-2581-sd7.pdf]
