# Supplementary material for: The MEKK1 PHD ubiquitinates TAB1 to activate MAPKs in response to cytokines
Source: EMBO J. 2014 Sep 26;33(21):2581–96. doi: 10.15252/embj.201488351 (PMC4282369; doi:10.15252/embj.201488351)
Supplement: Supplementary file 11 [file embj0033-2581-sd11.pdf]

Supplementary Information

Figure S1

Characterization of the MEKK1 PHD motif. (A) Quantitation of the relative MEKK1 and MEKK1 mPHD kinase activity in cells (□ control, ■ MEKK1 and ■ MEKK1 mPHD). (B) Identification of E2 conjugating enzymes for the MEKK1 PHD. Ubiquitination reactions were performed containing MEKK1 PHD, UBE2D2, UBE2D3, UBE2J1 and UBE2N:UBE2V1 as indicated. Reactions were analysed by IB using the indicated antibodies. MEKK1 PHD loading was confirmed by silver staining. (C) Identification of types of Ub formed by the MEKK1 PHD and E2 conjugating enzymes. Ubiquitination reactions were performed containing Ub, lysineless Ub, MEKK1 PHD, UBE2D2, UBE2D3, UBE2J1 and UBE2N:UBE2V1 as indicated. Reactions were analysed by IB using the indicated antibodies. MEKK1 PHD loading was confirmed by silver staining. (D) Identification of the DUB enzymes that can remove Ub from the MEKK1 PHD. Ubiquitination reactions containing Ub, MEKK1 PHD and UBE2N:UBE2V1 were performed After the ubiquitination reaction the following DUB enzymes were added Otubain, USP14, USP8, USP7, USP2, BAP1, A20, Ataxin-3, UCH-L3, UCH-L1 and Isopeptidase T as indicated. Reactions were analysed by IB using the indicated antibodies. MEKK1 PHD loading was confirmed by silver staining. (E) Yeast-two hybrid assay identifying the interaction between UBE2N and MEKK1. Y190 yeast were transformed by the indicated constructs. Results are representative of three independent experiments.

Figure S2

Map3k1^{mPHD} ES cells have normal SMAD activation following TGF-β treatment. (A) *WT* and *Map3k1*^{mPHD} ES cells were plated onto low serum media, rested and stimulated with TGF-β (10 ng/ml) for 10, 30 and 60 mins or left unstimulated. Cells were lysed and IB performed using the indicated antibodies. (B) MEKK1 stability is partially dependent upon the MEKK1 PHD. *WT* and *Map3k1*^{mPHD} ES cell clones were left unstimulated or stimulated for up to 8 hrs with 500 mM sorbitol. Lysates were prepared and analysed by IB with the indicated antibodies (arrow heads indicate MEKK1). (C) Schematic diagram illustrating MEKK1 PHD signaling in ES cells. Results are representative of three independent experiments.

Figure S3

MEKK1 PHD bioinformatics and *in vitro* ubiquitination analyses of protein array screen hits. (A) Ingenuity IPA tree diagram showing interactions of E1+E2+E3 protein array hits with components of the TGF-β signaling pathways. Key: complex, enzyme/scaffold, growth factor, kinase, transcriptional regulator and complex/group/other. Solid lines indicate binding only. Solid arrows indicate direct interactions. Dotted arrows indicate indirect interactions. (B) Ubiquitination assay was performed using UBE1, TAB1, MEKK1, MEKK1 mPHD and UBE2N:UBE2V1. Reactions were analysed by IB using the indicated antibodies. Lane 3 indicates substrate ubiquitination by the MEKK1 PHD and lane 5 indicates MEKK1 PHD autoubiquitination. (C) Ubiquitination assay was performed using UBE1, TNIP1, MEKK1, MEKK1 mPHD and UBE2N:UBE2V1. Reactions were analysed by IB using the indicated antibodies. (D) Ubiquitination assay was performed using UBE1, TNIP2, MEKK1, MEKK1 mPHD and UBE2N:UBE2V1. Reactions were analysed by IB using the indicated antibodies. (E) Ubiquitination assay was performed using UBE1, TRAF2, MEKK1, MEKK1 mPHD and

UBE2N:UBE2V1. Reactions were analysed by IB using the indicated antibodies. (F) Ubiquitination assay was performed using UBE1, STAM1, MEKK1, MEKK1 mPHD and UBE2N:UBE2V1. Reactions were analysed by IB using the indicated antibodies. Results are representative of three independent experiments.

Figure S4

Mechanism of MEKK1 PHD activation of MAPK signaling in ES cells. (A) The MEKK1 mPHD mutant is unable to induce TAB1 ubiquitination *in vivo*. HEK 293 cells were transiently transfected as indicated. 48 hrs later cells were lysed and analysed by IP and IB using the indicated antibodies. (B) MEKK1 and TAB1 copurify from cells. HEK 293 cells were transfected with the indicated constructs, lysates made and IB performed using the indicated antibodies. (C) Schematic illustrating the generation of *Tab1*$^{-/-}$ ES cells. (D) Expression of TAB1 in *Tab1*$^{-/-}$ ES cells. Lysates were made from *WT* and *Tab1*$^{-/-}$ ES cells and analysed by the indicated antibodies. (E) *WT* or *Tab1*$^{-/-}$ ES cells were kept in low serum conditions and stimulated or not for 10 mins with EGF (100 ng/ml). Lysates were made and IB performed using the indicated antibodies. (F) Schematic showing the deletion fragments used to detect the region of TAB1 ubiquitination by MEKK1 PHD (TAK1 indicates the TAK1 binding domain). (G) Mutation of TAB1 residues K294A, K319A, K335A and K350A (mTAB1) ablates TAB1 ubiquitination by MEKK1 in cells. HEK 293 cells were transfected with the indicated constructs. Lysates were made and IP and IB performed using the indicated antibodies. (H) Mutation of TAB1 residues K294A, K319A, K335A and K350A (mTAB1) ablates the TAB1:TAK1 association in cells. HEK 293 cells were transfected with the indicated constructs. Lysates were made and IP and IB were performed using the indicated antibodies. (I) endogenous MEKK1 was IP from ES cell lysates stimulated by TGF-β for 10 mins. Lysates and IP were IB with the indicated antibodies (J) *WT* or *Map3k1*^{mPHD} ES cells were treated with TGF-β or not for 10 mins. Lysates were made and IB performed using the indicated antibodies. Results are representative of three independent experiments.

Figure S5

MEKK1 ubiquitination of TAB1 enhances TAK1 activation. (A) Schematic diagram showing the reaction steps and components of the MEKK1 ubiquitination and TAK1 kinase assays. (B) Recombinant MEKK1, TAK1, MEKK1 mPHD and TAB1 were expressed and purified from sf9 cells and a fraction of the resulting input proteins were IB with the indicated antibodies. The indicated purified recombinant proteins were then used in ubiquitination assays with UBE1, UBE2N:UBE2V1 and Ub for 60 mins. Lys63-linked Ub in the reactions were identified by IB. Finally, recombinant TAK1 was added to the ubiquitinated proteins and kinase assays performed as indicated. The reactions were stopped in loading buffer after 30 mins and analysed by IB using the indicated antibodies. (C) A TAK1 activation assay was performed as before using TAK1, UBE1, UBE2N:UBE2V1 and Ub, and also MEKK1, TAB1 and mTAB1 as indicated. IB was performed using the indicated antibodies.

Figure S6

Global gene expression analysis of *Map3k1*^{mPHD} ES cells. (A) *WT* and *Map3k1*^{mPHD} ES cell microarray data sets were analysed using Ingenuity iReport. (B) Ingenuity IPA tree diagram showing interactions of Affymetrix hits with components of the TGF-β signaling pathway. Key: ○ other, ◇ enzyme, ▽ kinase, ⬭ transcription

regulator, ⏢ transporter, ◇ peptidase, ◎ complex/group. Solid lines indicate binding only. Solid arrows indicate direct interactions. Dotted arrows indicate indirect interactions. (C) ■ *WT* (unstimulated), ■ *WT* (day 9), ■ *Map3k1*^{mPHD} (unstimulated), ■ *Map3k1*^{mPHD} (day 9), ■ *Tab1*$^{-/-}$ (unstimulated) and ■ *Tab1*$^{-/-}$ ES cells (day 9) were left unstimulated or differentiated for 9 days. Real time PCR was performed using primers specific for the genes *Nanog* and *Oct4*. The average relative expression (± SEM) of the indicated gene mRNA from 3 independent experiments was statistically analysed, where appropriate, by two-tailed Student's t test (*, $p \leq 0.05$; ** $p \leq 0.01$; ***, $p \leq 0.001$). (D) ■ *WT* (unstimulated), ■ *WT* (day 9), ■ *Map3k1*^{mPHD} (unstimulated), ■ *Map3k1*^{mPHD} (day 9), ■ *Tab1*$^{-/-}$ (unstimulated) and ■ *Tab1*$^{-/-}$ (day 9) ES cells were plated under differentiation conditions without LIF, proliferation was measured by CFSE assay and analysed by FACS.

Figure S7

TGFβR expression analysis of *Map3k1*^{mPHD} ES cells. (A) Heat map showing *Tgfbr1*, *Tgfbr2* and *Tgfbr3* expression between pluripotent *WT* and *Map3k1*^{mPHD} ES cells. (B) Real time PCR showing gene expression between pluripotent ■ *WT*, ■ *Map3k1*^{mPHD} and ■ *Tab1*$^{-/-}$ ES cells for *TGFβR*s. The average relative expression (± SEM) of the indicated gene mRNA from 3 independent experiments was statistically analysed, where appropriate, by two-tailed Student's t test (*, $p \leq 0.05$; ** $p \leq 0.01$; ***, $p \leq 0.001$). (C) *WT*, *Map3k1*^{mPHD} and *Tab1*$^{-/-}$ ES cells were grown continuously on serum in the presence of LIF. Lysates were made and analysed by IB using the indicated antibodies.

Figure S8

Map3k1^{mPHD} and *Tab1*$^{-/-}$ ES cells exhibit an altered differentiation pattern compared to *WT* ES cells. ■ *WT*, ■ *Map3k1*^{mPHD} and ■ *Tab1*$^{-/-}$ ES cells were plated under differentiation conditions without LIF for 9 days, mRNA was extracted and used for real-time PCR using primers specific for (A) neuroectoderm, (B) endoderm and (C) mesoderm genes. (D) ■ DMSO, ■ SB203580 and ■ SB431542 treated *WT* ES cells were plated under differentiation conditions without LIF for 9 days, mRNA was extracted and used for real time PCR using primers specific for a mesoderm gene marker. (E) *Tab1*$^{-/-}$ ES cells were transfected with empty vector, TAB1 and mTAB1 as indicated. Transfected *Tab1*$^{-/-}$ and *WT* ES cells were lysed and IB was performed using the indicated antibodies. (F) *Tab1*$^{-/-}$ ES cells were transfected with ■ vector, ■ TAB1 or ■ mTAB1, and plated, alongside ■ *WT* ES cells, under differentiation conditions without LIF for 9 days, mRNA was extracted and used for real time PCR using primers specific for a mesoderm gene marker. The average relative expression (± SEM) of the indicated gene mRNA from 3 independent experiments was statistically analysed, where appropriate, by two-tailed Student's t test (*, $p \leq 0.05$; ** $p \leq 0.01$; ***, $p \leq 0.001$).

Figure S9

Analysis of MEKK1 PHD-dependent differentiation of *Tab1*$^{-/-}$ ES cells. *Tab1*$^{-/-}$ ES cells were transfected with ■CMV, ■CMV TAB1 or ■CMV mTAB1 and used alongside ■ *WT* ES cells in differentiation assays for 6 days, mRNA was extracted and their RNAs analysed by real time PCR with primers specific for the neuroectoderm genes *Nestin and Pax6*. The average relative expression (± SEM) of the indicated gene mRNA from 3 independent experiments was statistically analysed,

where appropriate, by two-tailed Student's t test (*, $p \leq 0.05$; ** $p \leq 0.01$; ***, $p \leq 0.001$).

Figure S10

Characterization of *Map3k1*$^{mPHD/+}$ mice. (A) Bone marrow was harvested from *WT* and *Map3k1*$^{mPHD/+}$ mice. Cells were stained with antibodies for the B cell markers IL-7R, CD34, CD45, CD38 and IgM and analysed by FACS. (B) Thymi was extracted from *WT* and *Map3k1*$^{mPHD/+}$ mice. (C) Quantitation of the mass of thymi from ■ *WT* and ■ *Map3k1*$^{mPHD/+}$ mice. The average relative expression (± SEM) of the thymi from 3 independent experiments was statistically analysed by two-tailed Student's t test. Results are representative of three independent experiments.

Table S1. Listing of hits from Affymetrix screening (ArrayExpress accession: E-MTAB-1679). Hits indicate a 2-fold or more difference in gene expression between *Map3k1*^{mPHD} and *WT* ES cells.

ID	Fold change	p-value	Entrez Gene Name	Location	Type(s)
A2m	-2.0057547	0.001857916	alpha-2-macroglobulin	Extracellular Space	transporter
Acta1	2.7550673	1.65E-04	actin, alpha 1, skeletal muscle	Cytoplasm	other
Actc1	3.3069584	0.002083707	actin, alpha, cardiac muscle 1	Cytoplasm	enzyme
Anxa3	3.8823948	0.002311585	annexin A3	Cytoplasm	enzyme
Anxa5	2.3316228	0.009822967	annexin A5	Plasma Membrane	other
Car4	2.0156875	0.003673881	carbonic anhydrase IV	Plasma Membrane	enzyme
Cald1	2.2011347	0.008693812	caldesmon 1	Plasma Membrane	other
Capn2	2.4027152	0.008644544	calpain 2, (m/II) large subunit	Cytoplasm	peptidase
Ccl2	-2.390797	0.003938371	chemokine (C-C motif) ligand 13	Extracellular Space	cytokine
Chst15	2.0687666	0.020435842	carbohydrate (N-acetylgalactosamine 4-sulfate 6-O) sulfotransferase 15	Plasma Membrane	enzyme
Clcn5	2.0605805	0.007213574	chloride channel, voltage-sensitive 5	Plasma Membrane	ion channel
Clu	2.2093937	0.001087975	clusterin	Extracellular Space	other
Cnn1	3.1327963	0.002744793	calponin 1, basic, smooth muscle	Cytoplasm	other
Cntfr	2.2550151	3.65E-05	ciliary neurotrophic factor receptor	Plasma Membrane	transmembrane receptor
Colec12	2.4210675	0.002105767	collectin sub-family member 12	Plasma Membrane	transmembrane receptor
Cryab	2.0318692	0.012150836	crystallin, alpha B	Nucleus	other
Csrp1	2.3107538	0.002873714	cysteine and glycine-rich protein 1	Nucleus	other
Cxcl11	2.048385	0.018570151	chemokine (C-X-C motif) ligand 11	Extracellular Space	cytokine
Cyr61	2.2087812	7.37E-04	cysteine-rich, angiogenic inducer, 61	Extracellular Space	other
Ddx3y	-2.9596515	5.51E-05	DEAD (Asp-Glu-Ala-Asp) box polypeptide 3, Y-linked	Cytoplasm	other
Dock11	2.1563225	8.86E-04	dedicator of cytokinesis 11	Cytoplasm	other
Dsc2	2.1575687	0.008196608	desmocollin 2	Plasma Membrane	other
Dsp	3.303479	0.0052969	desmoplakin	Plasma Membrane	other
Dusp14	2.0176184	6.26E-04	dual specificity phosphatase 14	unknown	phosphatase
Dusp4	2.5989609	6.06E-04	dual specificity phosphatase 4	Nucleus	phosphatase
Edn1	3.0703537	0.004326449	endothelin 1	Extracellular Space	cytokine
Efemp1	2.4148428	0.004399272	EGF containing fibulin-like extracellular matrix protein 1	Extracellular Space	enzyme
Efna5	2.0107615	0.01767725	ephrin-A5	Plasma Membrane	kinase
Eif2s3y	-2.784723	0.005807369	eukaryotic translation initiation factor 2, subunit 3, structural gene Y-linked	Cytoplasm	translation regulator
F3	2.4208796	0.009203905	coagulation factor III (thromboplastin, tissue factor)	Plasma Membrane	transmembrane receptor
Fbln2	-2.2116966	0.004935371	fibulin 2	Extracellular Space	other
Fermt1	2.1215284	0.001388512	fermitin family member 1	Plasma Membrane	other
Flnc	2.6198037	5.60E-04	filamin C, gamma	Cytoplasm	other
Fndc3c1	2.887473	0.001202381	fibronectin type III domain containing 3C1	unknown	other
Gbp1	3.264705	1.87E-04	guanylate binding protein 2, interferon-inducible	Nucleus	other
Gbp2	4.6560364	0.006907613	guanylate binding protein 2	unknown	enzyme
Gprc5c	2.4646301	0.012449834	G protein-coupled receptor, family C, group 5, member C	Plasma Membrane	G-protein coupled receptor
Grem1	-2.8356338	0.010599915	gremlin 1	Extracellular Space	other
Igfbp3	4.294441	1.47E-04	insulin-like growth factor binding protein 3	Extracellular Space	other
Itga3	2.5575054	0.004522102	integrin, alpha 3 (antigen CD49C, alpha 3 subunit of VLA-3 receptor)	Plasma Membrane	other
Krt18	5.0943055	8.91E-04	keratin 18	Cytoplasm	other
Krt19	4.536249	0.001772012	keratin 19	Cytoplasm	other
Krt8	6.0431256	5.95E-04	keratin 8	Cytoplasm	other
Lamc2	2.3562734	0.011176528	laminin, gamma 2	Extracellular Space	other
Lphn2	2.8834867	4.44E-05	latrophilin 2	Plasma Membrane	G-protein coupled receptor
Mir92-1	-2.2976387	0.00830584	microRNA 25	Cytoplasm	microRNA
Nefl	3.2824752	0.004686247	neurofilament, light polypeptide	Cytoplasm	other
Nes	3.2208774	2.86E-04	nestin	Cytoplasm	other
Nnat	2.564566	3.38E-05	neuronatin	Plasma Membrane	transporter
Nt5e	3.767016	3.96E-04	5'-nucleotidase, ecto (CD73)	Plasma Membrane	phosphatase
Nuak1	2.382953	0.003435363	NUAK family, SNF1-like kinase, 1	unknown	kinase
Otx2	-2.4149704	8.42E-05	orthodenticle homeobox 2	Nucleus	transcription regulator
Parva	2.030465	0.008783761	parvin, alpha	Cytoplasm	other
Pcdh18	2.0676134	0.015142876	protocadherin 18	Extracellular Space	other
Phldb2	2.2002082	0.002353882	pleckstrin homology-like domain, family B, member 2	Cytoplasm	other
Ppp4r4	2.6705232	0.00665947	protein phosphatase 4, regulatory subunit 4	Cytoplasm	other
Prmt8	-2.31923	0.011715327	protein arginine methyltransferase 8	Nucleus	enzyme
Prtg	2.2732003	8.05E-04	protogenin	unknown	other
Ptgs2	-2.0603693	0.006673997	prostaglandin-endoperoxide synthase 2 (prostaglandin G/H synthase and cyclooxygenase)	Cytoplasm	enzyme
Rdh10	2.228082 \		retinol dehydrogenase 10 (all-trans)	Nucleus	enzyme
Rnf128	2.1034398	0.014772559	ring finger protein 128, E3 ubiquitin protein ligase	Cytoplasm	enzyme
Runx1	2.1293156	4.98E-04	runt-related transcription factor 1	Nucleus	transcription regulator
Sema6a	2.8991046	0.004744235	sema domain, transmembrane domain (TM), and cytoplasmic domain, (semaphorin) 6A	Plasma Membrane	transmembrane receptor
Sfn	4.6045985	0.002139192	stratifin	Cytoplasm	other
Slc25a24	2.5912123	0.001341933	solute carrier family 25 (mitochondrial carrier; phosphate carrier), member 24	Cytoplasm	other
Sp5	2.2794976	0.040503327	Sp5 transcription factor	Nucleus	other
Sprr2a1	4.5782948	5.27E-05	small proline-rich protein 2G	Cytoplasm	other
T	7.005557	0.002097362	T, brachyury homolog (mouse)	Nucleus	transcription regulator
Tagln	2.737775	0.004483641	transgelin	Cytoplasm	other
Tax1bp3	2.0935597	0.003977545	Tax1 (human T-cell leukemia virus type I) binding protein 3	Nucleus	transcription regulator
Tec	2.0375817	7.56E-04	tec protein tyrosine kinase	Cytoplasm	kinase
Tes	2.0066843	1.40E-04	testis derived transcript (3 LIM domains)	Plasma Membrane	other
Tgfb2	2.9551053	0.011359884	transforming growth factor, beta 2	Extracellular Space	growth factor
Tpm1	2.6363387	0.001206219	tropomyosin 1, alpha	Plasma Membrane	other
Uty	-3.2841432	7.41E-04	ubiquitously transcribed tetratricopeptide repeat gene, Y chromosome	Nucleus	other
Wnt3	2.3104796	0.003809417	wingless-type MMTV integration site family, member 3	Extracellular Space	other

Table S2. List of hits from the E1+E2 protein array ranked by their Z score from highest to lowest.

**Gene ID**	**Z score**
PELI1	18.719
RNF4	18.703
RNF34	18.692
UBE3A	18.687
BIRC4	18.685
TRIM11	18.681
BIRC7	18.679
BIRC7	18.667
RNF111	18.652
BIRC3	18.645
UBE2O	18.641
RNF34	18.639
STUB1	18.627
RNF13	18.615
UBE3A	18.609
UBE2C	17.609
LRSAM1	15.968
LRSAM1	14.790
TRIM21	14.143
RFWD3	13.342
PELI2	13.169
NHEDC2	11.861
RNF135	11.674
NUDT6	11.541
CBLB	10.908
UBE2E2	10.631
TSPAN17	10.090
UBE2O	9.253
POLI	8.642
FLJ20160	7.630
UBE2T	7.282
DBF4B	6.787
ACOX1	6.471
UBE2D3	5.724
UBE2B	4.650
UBE2E1	4.599
UBE2S	4.353
ANKRD13A	4.308
CALM2	4.277
INTS3	4.190
UBE1	4.132
ANKRD13D	4.129
TRIM52	3.855
UBE2D2	3.764
RNF11	3.721

RNF122	3.649
UBE2R2	3.632
UBQLN4	3.558
RHBDD1	3.438
TRIM23	3.393
SMURF1	3.333
RIOK3	3.277
RBP2	3.181
THRA	3.052
RNF182	3.026

Table S3. List of hits from the E1+E2+E3 protein array ranked by their Z score from highest to lowest.

**Gene ID**	**Z score**
BAP1	10.806
FKBP6	10.784
NPLOC4	10.782
RIOK3	10.781
ASCC2	10.780
UBE2V1	10.779
UBADC1	10.778
DNAJB6	10.776
TOM1L2	10.776
CUEDC1	10.776
HBS1L	10.775
SQSTM1	10.774
RHBDD1	10.774
APPL1	10.774
MSL3L1	10.773
ZFAND5	10.773
TRAF2	10.771
TNIP2	10.770
TOM1	10.770
RAD23A	10.770
TNIP2	10.769
UBXD1	10.768
STAM1	10.768
UBQLN4	10.767
CD74	10.765
HIP1	10.764
UBXN7	11.275
HGS	11.267
C6orf106	11.262
RAB3IL1	11.250
STS-1	11.208
TAB1	10.240
DNAJB2	9.964
RNF141	9.858
FAM188A	9.835
UBAP1	9.219
DCUN1D1	9.652
NEK10	9.152
STAM2	8.848
GRIPAP1	8.688
YAF2	8.640
UBXD1	9.057
EPHA1	8.760
SQSTM1	8.237
UBE2D3	7.894

RAD18	8.142
RBCK1	8.121
IMPACT	7.651
HIP1R	7.921
PSMD4	7.475
HIP1R	7.842
YAF2	7.424
SPG20	7.779
ZFAND3	7.509
EPN1	6.548
ZNF313	6.528
UBE2G1	6.425
DCUN1D2	6.664
UCHL5	6.275
CRELD1	6.252
ATXN3	6.538
UBXD8	6.045
CCDC21	5.837
UFD1L	5.431
UBE2K	5.060
MSL3L1	5.145
USP28	4.785
FAM184A	4.371
GGA2	4.437
PLEKHB2	4.236
KRT36	4.142
RNF126	3.905
APEX2	3.857
C20orf18	4.057
ODF2	3.623
FAM116B	3.508
CARD14	3.398
UBQLN1	3.275
CCDC69	3.225
RABEP2	3.210
RAD51AP1	4.228
TNIP1	3.899

Table S4. List of primers used in this study.

Gene	Forward (5' – 3')	Reverse (5' – 3')
Brachyury	CCGGTGCTGAAGGTAAATGT	CCTCCATTGAGCTTGTTGGT
Gata4	AACACCCTGAGCAGGCCTC	TTTCTGGGAAACTGGAGCTGG
Gata6	ATATGGCCTTGACTGACGGCG	ATTCAGGCCAGGGCCAGAGCAC
L19	TGATCTGCTGACGGAGTTG	GGAAAAGAAGGTCTGGTTGGA
Mash1	CCAGGCTGGAGCAAGGGA	CGGTTGGCTTCGGGAGC
Mixl1	GACAGACCATGTACCCAGAC	GCTTCAAACACCTAGCTTCAG
Nanog	CTTACAAGGGTCTGCTACTGA	CTGCTTCCTGGCAAGGACC
Nestin	CTACATACAGGACTCTGCTGG	CTCAGACATAGGTGGGATGG
Oct4	CACGAGTGGAAAGCAACTCA	AGATGGTGGTCTGGCTGAAC
Pax6	ACACGCCCTGGTTGGTATC	CATCTGAGCTTCATCCGAGTC
Ddx3y	GGAAACAGGTCTACGGTGGC	GCGCCCTTTGCTCTCTGTATT
Otx2	GGGTGCAGGTATGGTTTAAGA	GCAATGGTTGGGACTGAGGTA
Dusp14	CTGTAACAAGCACCGCTCCC	CCTCCTTCGTGCTAAGGATTTC
Tec	TCTGTCTTGGCTTGTCTCGG	ACGATCTCCGGATTCCCTCT
Nnat	GCGAGAAGTGAGGTGTTCAG	CAGGAGCACCTGATGACACG
Dusp4	ACCAGTACAAGTGCATCCCC	GTCCTTTACTGCGTCGATGT
Acta1	CATGTGCGACGAAGACGAGA	CATACCTACCATGACACCCTGG
Tgfb2	AATGGCTCTCCTTCGACGTG	AGGTGCCATCAATACCTGCAA
Nuak1	ATTGTCAACGGCAGGCCTTA	CCGAGCATCTGAGGGTTGTG
Runx1	CAGGCAGGACGAATCACACT	CTCGTGCTGGCATCTCTCA
Tagln	CGGCCTTTAAACCCCTCACC	CATGTTGAGGCAGAGAAGGCT
Tgfbr1	GGCGAAGGCATTACAGTGTTT	ATGACAGTGCGGTTATGGCA
Tgfbr2	TTTCCTGTTTCCCTCTCGGC	GCTTCCATTTCCACATCCGAC
Tgfbr3	GCGGAGTACCTTCAACCCAA	CCGAGTAGCCATTGGTCTGG

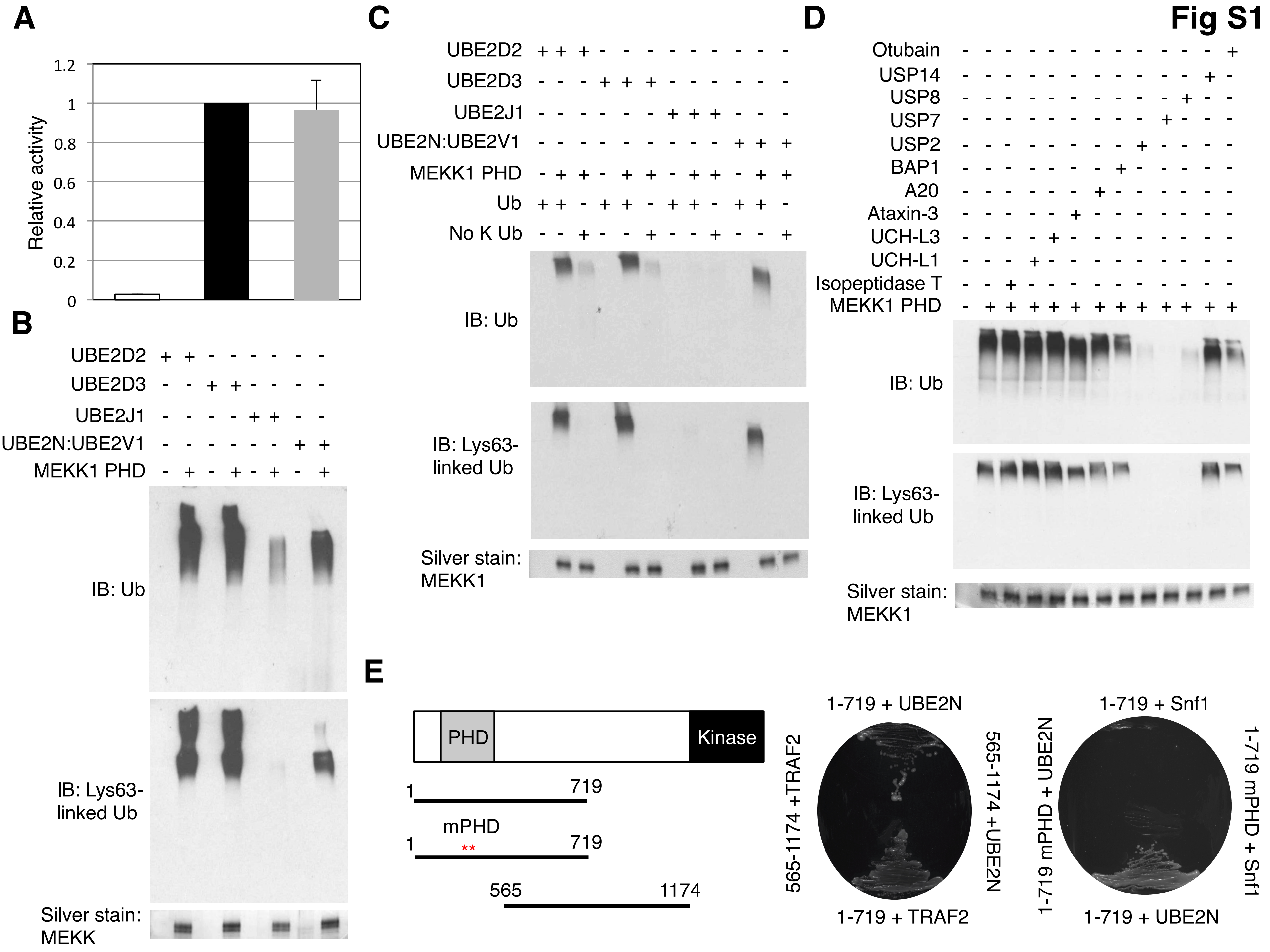

Fig S1
A
Relative activity
1.2
1
0.8
0.6
0.4
0.2
0
B
UBE2D2 + + - - - - - -
UBE2D3 - - + + - - - -
UBE2J1 - - - - + + - -
UBE2N:UBE2V1 - - - - - - + +
MEKK1 PHD - + - + - + - +
IB: Ub
IB: Lys63-linked Ub
Silver stain: MEKK
C
UBE2D2 + + + - - - - - - - - -
UBE2D3 - - - + + + - - - - - -
UBE2J1 - - - - - - + + + - - -
UBE2N:UBE2V1 - - - - - - - - - + + +
MEKK1 PHD - + + - + + - + + - + +
Ub + + - + + - + + - + + -
No K Ub - - + - - + - - + - - +
IB: Ub
IB: Lys63-linked Ub
Silver stain: MEKK1
D
Otubain - - - - - - - - - - - - +
USP14 - - - - - - - - - - - + -
USP8 - - - - - - - - - - + - -
USP7 - - - - - - - - - + - - -
USP2 - - - - - - - - + - - - -
BAP1 - - - - - - - + - - - - -
A20 - - - - - - + - - - - - -
Ataxin-3 - - - - - + - - - - - - -
UCH-L3 - - - - + - - - - - - - -
UCH-L1 - - - + - - - - - - - - -
Isopeptidase T - - + - - - - - - - - - -
MEKK1 PHD - + + + + + + + + + + + +
IB: Ub
IB: Lys63-linked Ub
Silver stain: MEKK1
E
PHD
Kinase
1
719
mPHD
1
719
565
1174
1-719 + UBE2N
565-1174 +TRAF2
565-1174 +UBE2N
1-719 + TRAF2
1-719 + Snf1
1-719 mPHD + UBE2N
1-719 mPHD + Snf1
1-719 + UBE2N

Fig S2

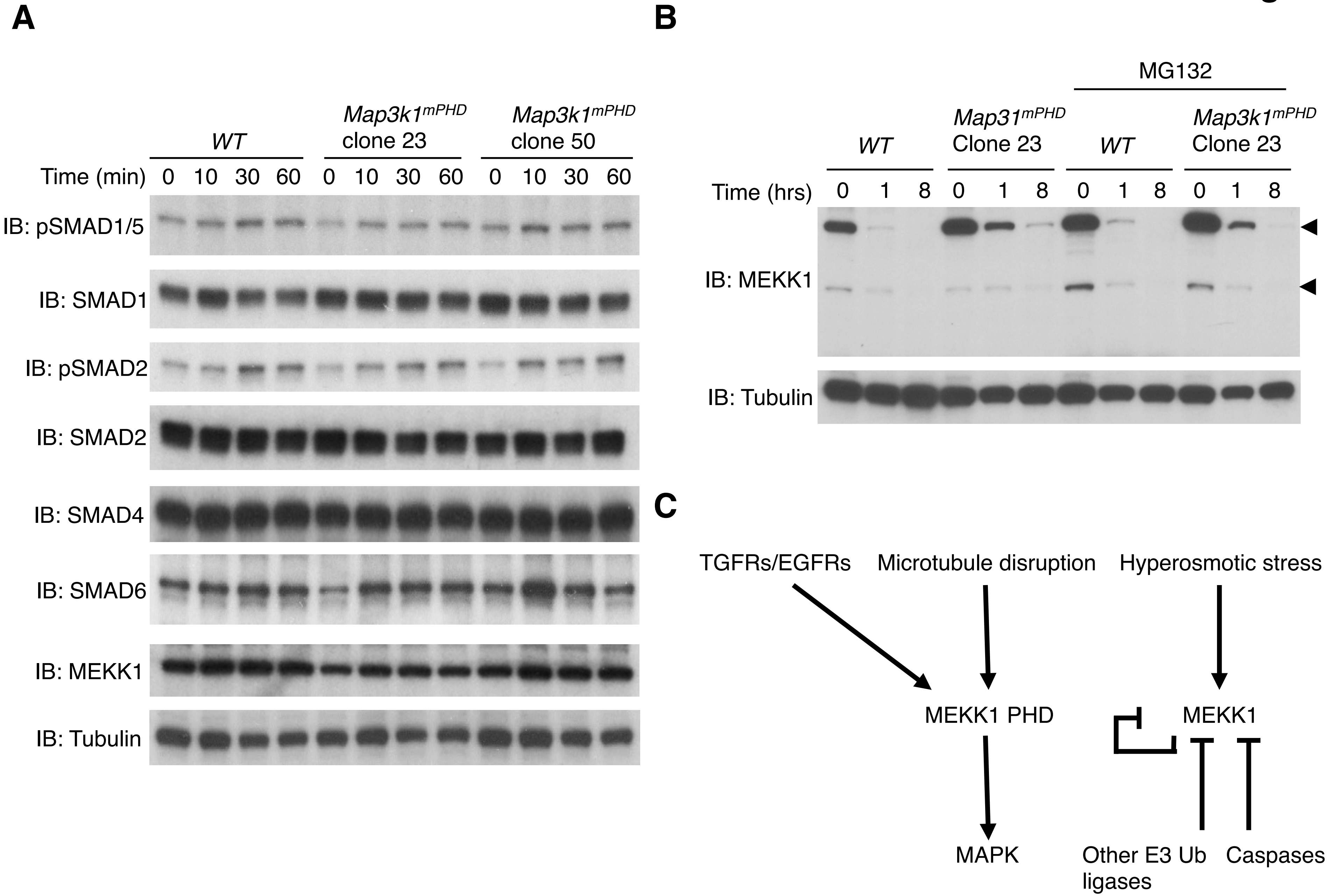
A
Map3k1mPHD
Map3k1mPHD
WT
clone 23
clone 50
Time (min) 0 10 30 60 0 10 30 60 0 10 30 60
IB: pSMAD1/5
IB: SMAD1
IB: pSMAD2
IB: SMAD2
IB: SMAD4
IB: SMAD6
IB: MEKK1
IB: Tubulin
B
MG132
Map31mPHD
Map3k1mPHD
WT
Clone 23
WT
Clone 23
Time (hrs) 0 1 8 0 1 8 0 1 8 0 1 8
IB: MEKK1
IB: Tubulin
C
TGFRs/EGFRs
Microtubule disruption
Hyperosmotic stress
MEKK1 PHD
MEKK1
MAPK
Other E3 Ub ligases
Caspases

Fig S3

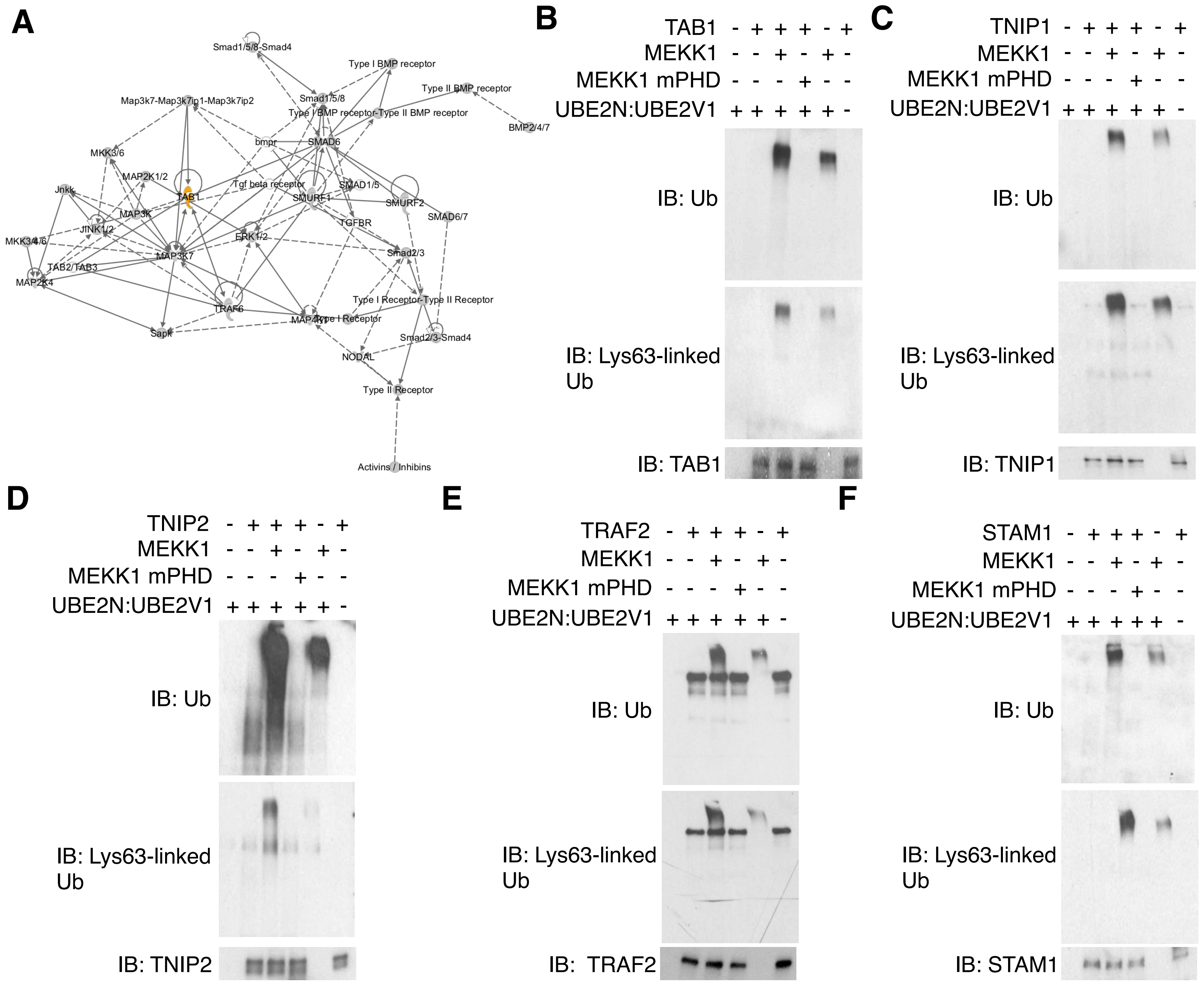

A
Smad1/5/8-Smad4
Type I BMP receptor
Type II BMP receptor
Smad1/5/8
Map3k7-Map3k7ip1-Map3k7ip2
Type I BMP receptor-Type II BMP receptor
BMP2/4/7
bmpr
SMAD6
MKK3/6
MAP2K1/2
Jnkk
TAB1
Tgf beta receptor
SMAD1/5
SMURF1
SMURF2
MAP3K
SMAD6/7
JNK1/2
TGFBR
MKK3/4/6
ERK1/2
MAP3K7
Smad2/3
TAB2/TAB3
MAP2K4
Type I Receptor-Type II Receptor
TRAF6
MAP4K1
Type I Receptor
Sapk
Smad2/3-Smad4
NODAL
Type II Receptor
Activins / Inhibins
B
TAB1 - + + + - +
MEKK1 - - + - + -
MEKK1 mPHD - - - + - -
UBE2N:UBE2V1 + + + + + -
IB: Ub
IB: Lys63-linked Ub
IB: TAB1
C
TNIP1 - + + + - +
MEKK1 - - + - + -
MEKK1 mPHD - - - + - -
UBE2N:UBE2V1 + + + + + -
IB: Ub
IB: Lys63-linked Ub
IB: TNIP1
D
TNIP2 - + + + - +
MEKK1 - - + - + -
MEKK1 mPHD - - - + - -
UBE2N:UBE2V1 + + + + + -
IB: Ub
IB: Lys63-linked Ub
IB: TNIP2
E
TRAF2 - + + + - +
MEKK1 - - + - + -
MEKK1 mPHD - - - + - -
UBE2N:UBE2V1 + + + + + -
IB: Ub
IB: Lys63-linked Ub
IB: TRAF2
F
STAM1 - + + + - +
MEKK1 - - + - + -
MEKK1 mPHD - - - + - -
UBE2N:UBE2V1 + + + + + -
IB: Ub
IB: Lys63-linked Ub
IB: STAM1

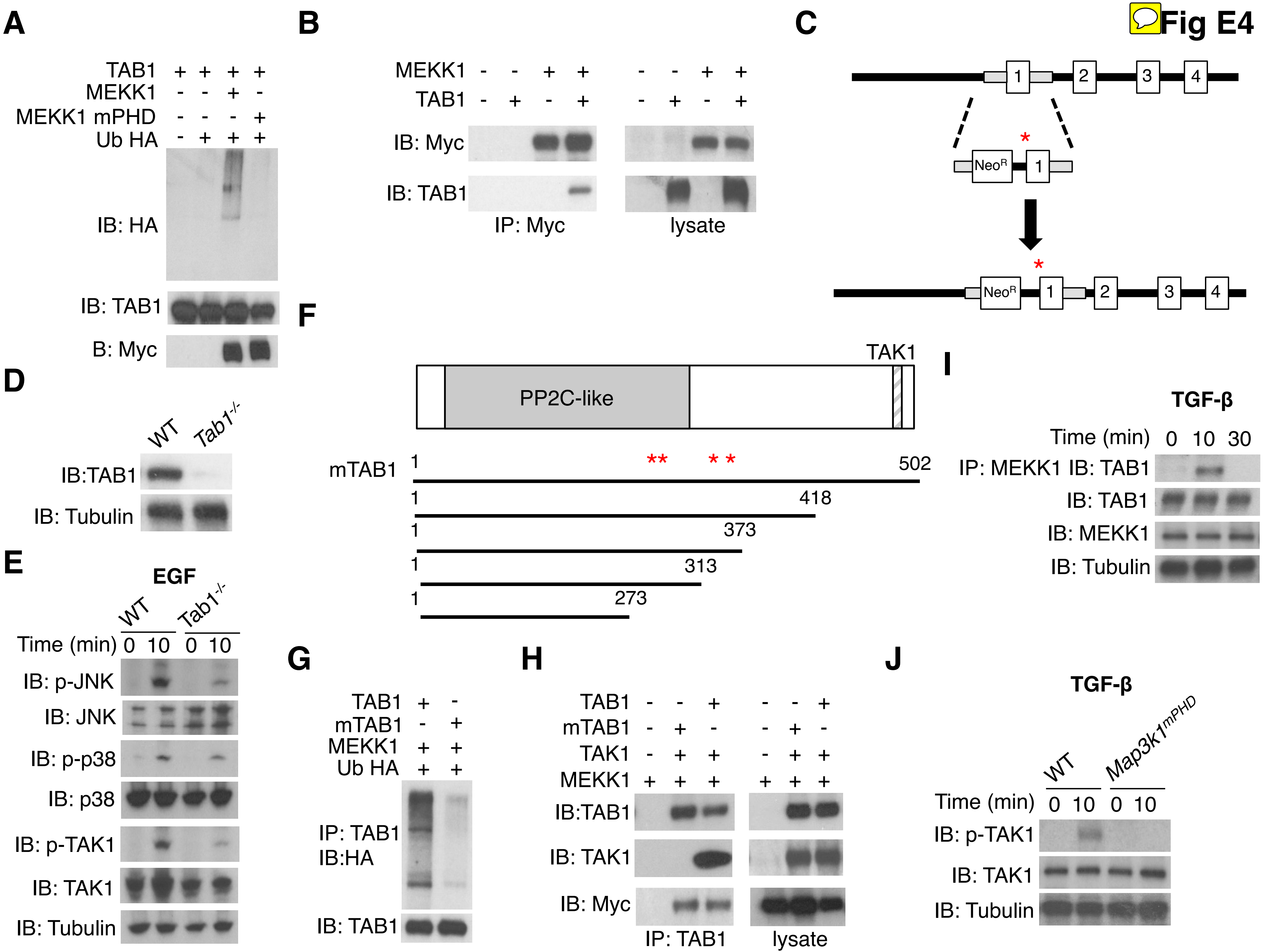
Fig E4
A
TAB1 + + + +
MEKK1 - - + -
MEKK1 mPHD - - - +
Ub HA - + + +
IB: HA
IB: TAB1
B: Myc
B
MEKK1 - - + + - - + +
TAB1 - + - + - + - +
IB: Myc
IB: TAB1
IP: Myc
lysate
C
1
2
3
4
NeoR
D
WT
Tab1-/-
IB:TAB1
IB: Tubulin
E
EGF
WT
Tab1-/-
Time (min) 0 10 0 10
IB: p-JNK
IB: JNK
IB: p-p38
IB: p38
IB: p-TAK1
IB: TAK1
IB: Tubulin
F
TAK1
PP2C-like
mTAB1
1
502
418
373
313
273
G
TAB1 + -
mTAB1 - +
MEKK1 + +
Ub HA + +
IP: TAB1
IB:HA
IB: TAB1
H
TAB1 - - + - - +
mTAB1 - + - - + -
TAK1 - + + - + +
MEKK1 + + + + + +
IB:TAB1
IB: TAK1
IB: Myc
IP: TAB1
lysate
I
TGF-β
Time (min) 0 10 30
IP: MEKK1 IB: TAB1
IB: TAB1
IB: MEKK1
IB: Tubulin
J
TGF-β
WT
Map3k1mPHD
Time (min) 0 10 0 10
IB: p-TAK1
IB: TAK1
IB: Tubulin

Fig S5

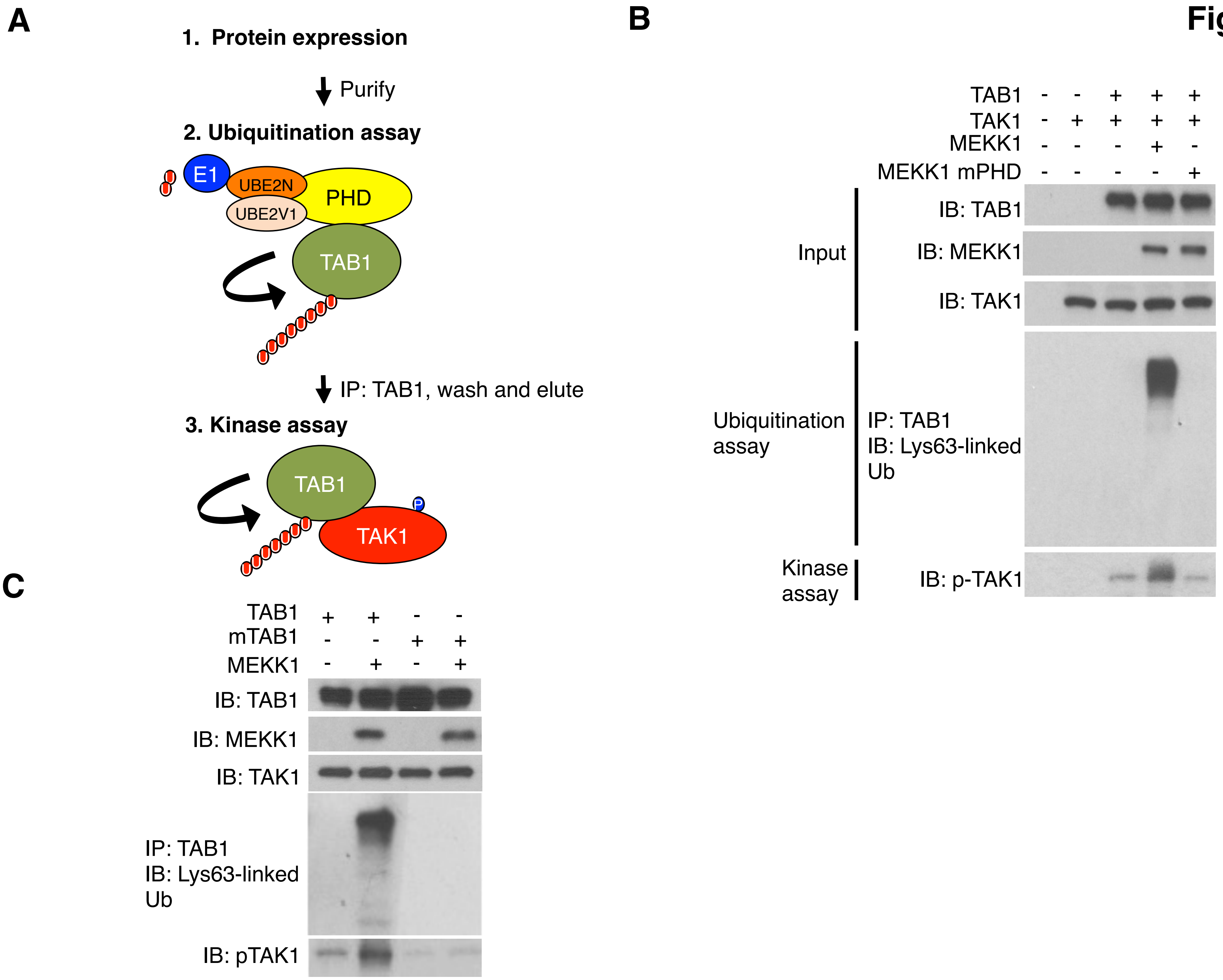
A
1. Protein expression
Purify
2. Ubiquitination assay
E1
UBE2N
UBE2V1
PHD
TAB1
IP: TAB1, wash and elute
3. Kinase assay
TAB1
P
TAK1
B
TAB1 - - + + +
TAK1 - + + + +
MEKK1 - - - + -
MEKK1 mPHD - - - - +
Input
IB: TAB1
IB: MEKK1
IB: TAK1
Ubiquitination assay
IP: TAB1
IB: Lys63-linked Ub
Kinase assay
IB: p-TAK1
C
TAB1 + + - -
mTAB1 - - + +
MEKK1 - + - +
IB: TAB1
IB: MEKK1
IB: TAK1
IP: TAB1
IB: Lys63-linked Ub
IB: pTAK1

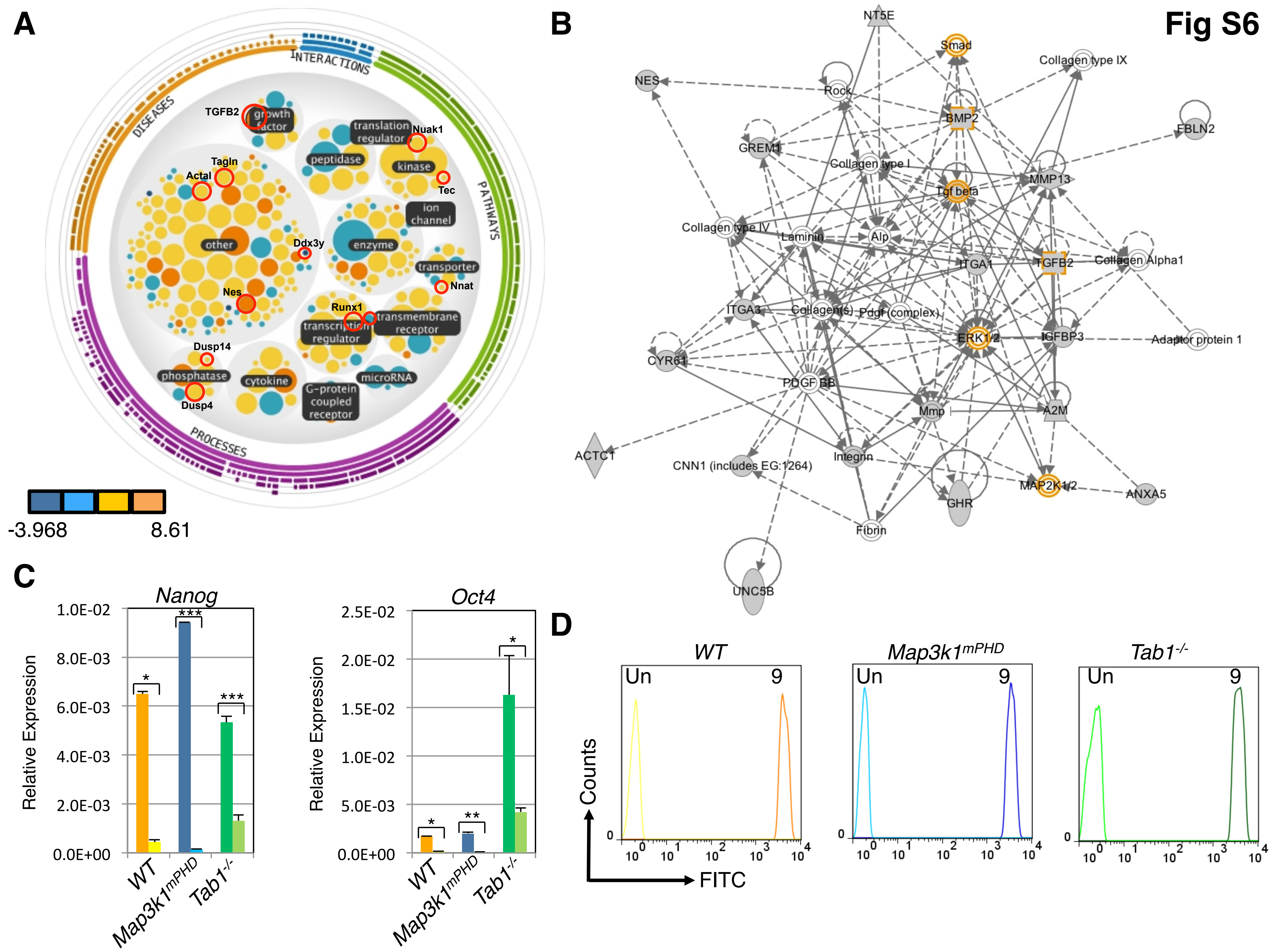
Fig S6
A
INTERACTIONS
DISEASES
PATHWAYS
PROCESSES
TGFB2
growth factor
translation regulator
Nuak1
peptidase
kinase
Tec
Tagln
Actal
ion channel
other
Ddx3y
enzyme
transporter
Nnat
Nes
Runx1
transcription regulator
transmembrane receptor
Dusp14
phosphatase
cytokine
microRNA
G-protein coupled receptor
Dusp4
-3.968
8.61
B
NT5E
Smad
Collagen type IX
NES
Rock
BMP2
FBLN2
GREM1
Collagen type I
MMP13
Tgf beta
Collagen type IV
Laminin
Alp
ITGA1
TGFB2
Collagen Alpha1
ITGA3
Collagen(s)
Pdgf (complex)
ERK1/2
IGFBP3
Adaptor protein 1
CYR61
PDGF BB
Mmp
A2M
ACTC1
CNN1 (includes EG:1264)
Integrin
MAP2K1/2
ANXA5
GHR
Fibrin
UNC5B
C
Nanog
Oct4
Relative Expression
1.0E-02
8.0E-03
6.0E-03
4.0E-03
2.0E-03
0.0E+00
2.5E-02
2.0E-02
1.5E-02
1.0E-02
5.0E-03
*
***
**
WT
Map3k1mPHD
Tab1-/-
D
WT
Map3k1mPHD
Tab1-/-
Un
9
Counts
FITC
0
10^0 10^1 10^2 10^3 10^4

Fig S7

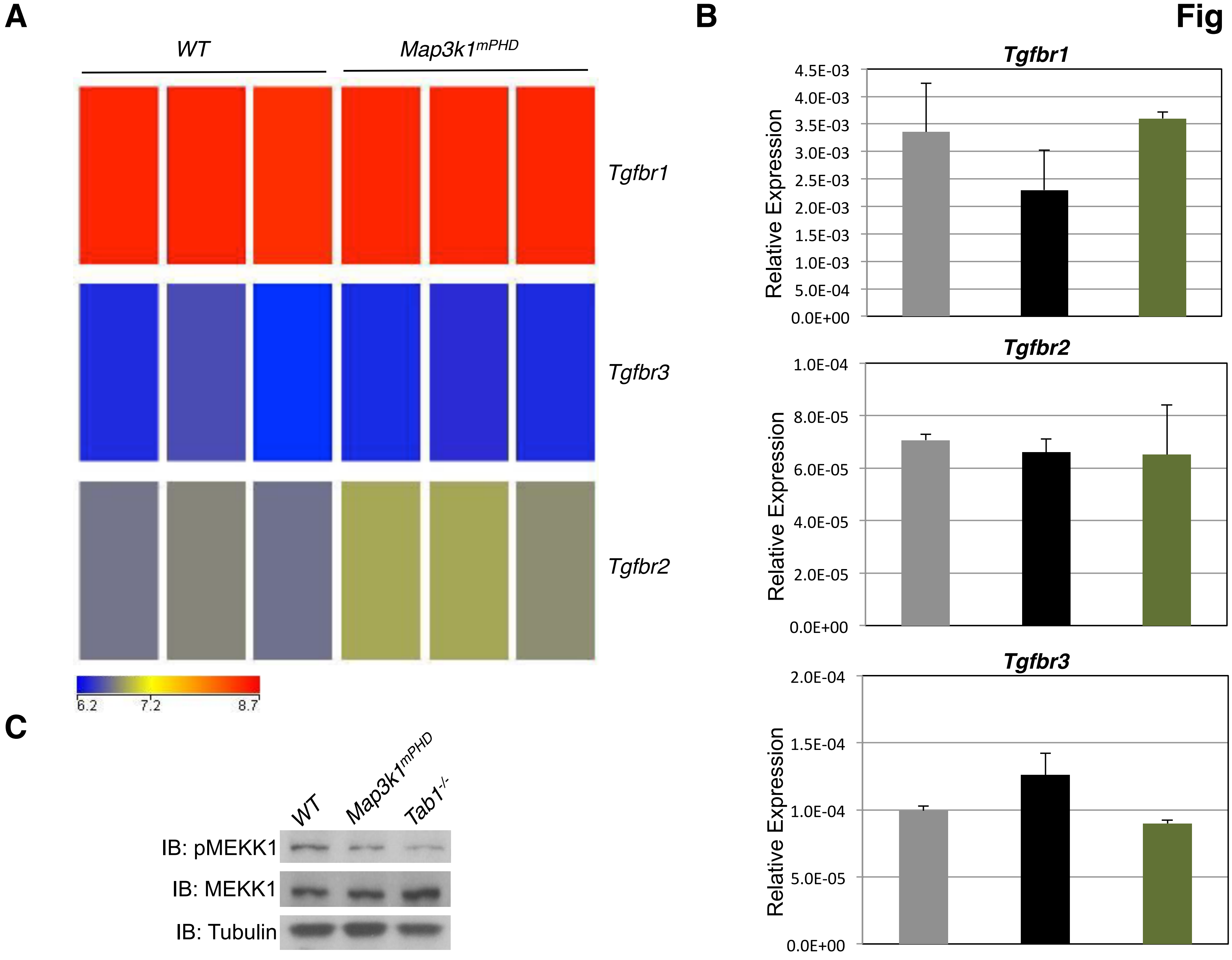
A
WT
Map3k1mPHD
Tgfbr1
Tgfbr3
Tgfbr2
6.2
7.2
8.7
B
Tgfbr1
Relative Expression
4.5E-03
4.0E-03
3.5E-03
3.0E-03
2.5E-03
2.0E-03
1.5E-03
1.0E-03
5.0E-04
0.0E+00
Tgfbr2
1.0E-04
8.0E-05
6.0E-05
4.0E-05
2.0E-05
0.0E+00
Tgfbr3
2.0E-04
1.5E-04
1.0E-04
5.0E-05
0.0E+00
C
WT
Map3k1mPHD
Tab1-/-
IB: pMEKK1
IB: MEKK1
IB: Tubulin

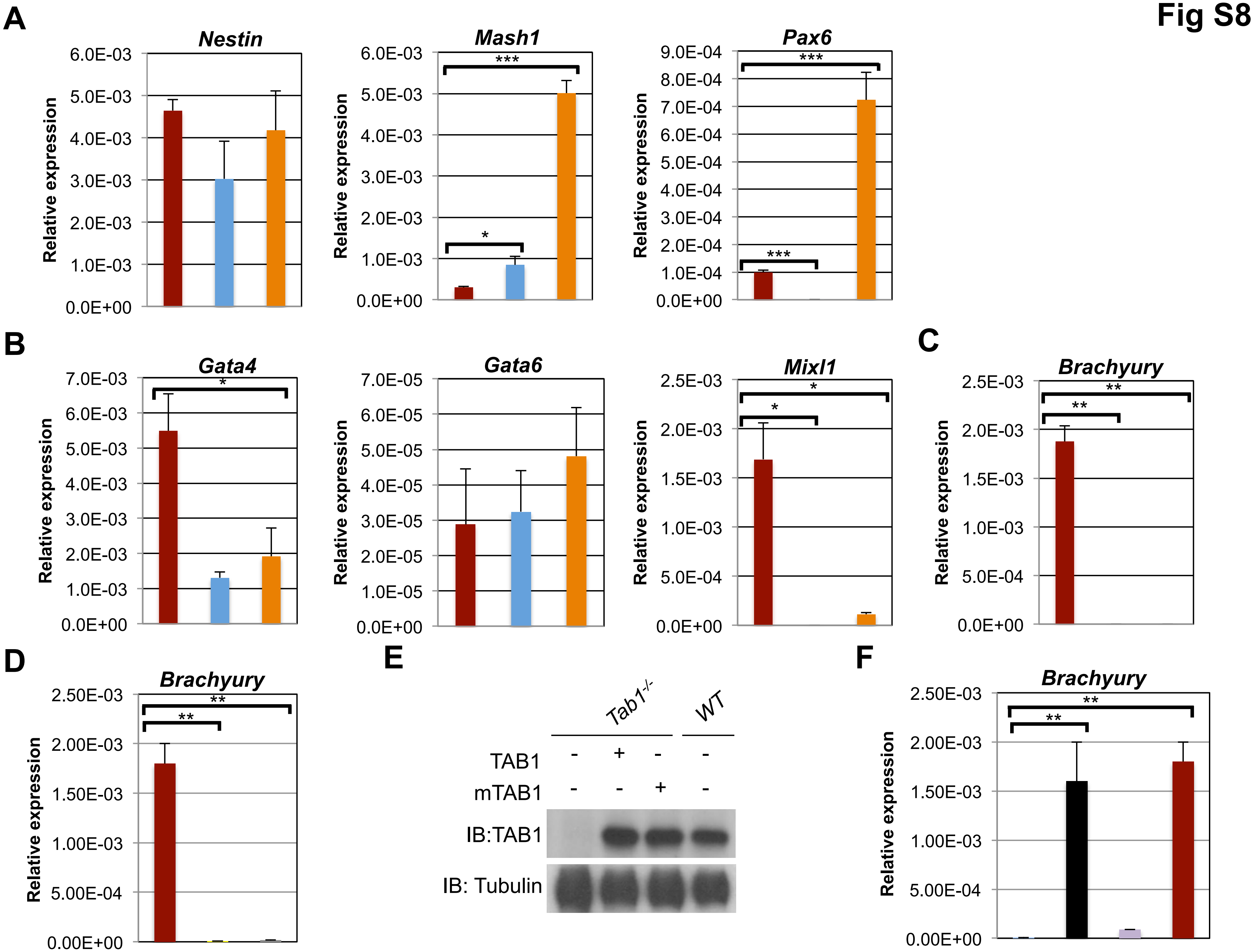
Fig S8
A
Nestin
Mash1
Pax6
B
Gata4
Gata6
Mixl1
C
Brachyury
D
Brachyury
E
Tab1-/-
WT
TAB1
mTAB1
IB:TAB1
IB: Tubulin
F
Brachyury
Relative expression

Fig S9

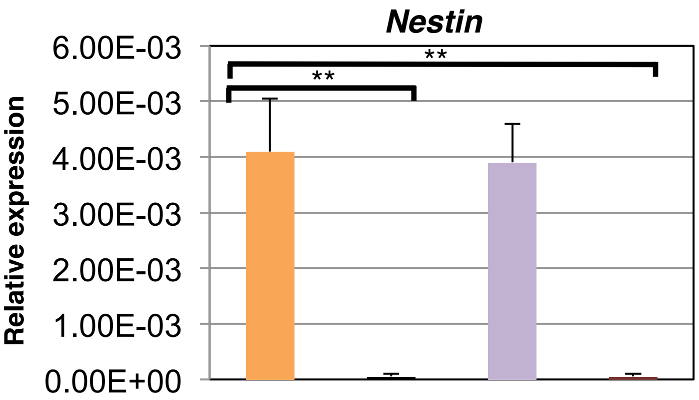
Nestin
Relative expression
6.00E-03
5.00E-03
4.00E-03
3.00E-03
2.00E-03
1.00E-03
0.00E+00
**
**

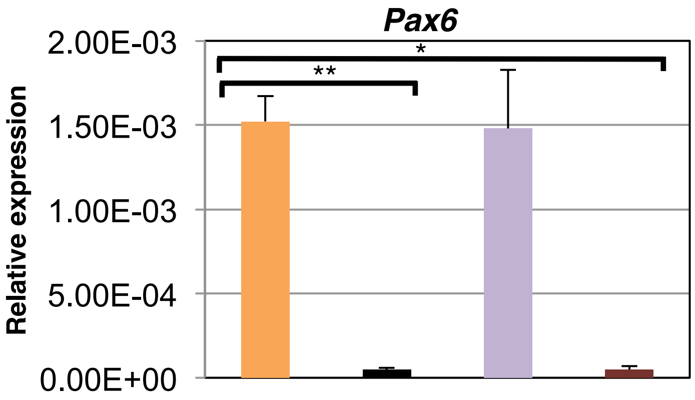
Pax6
Relative expression
2.00E-03
1.50E-03
1.00E-03
5.00E-04
0.00E+00
*
**

Fig S10

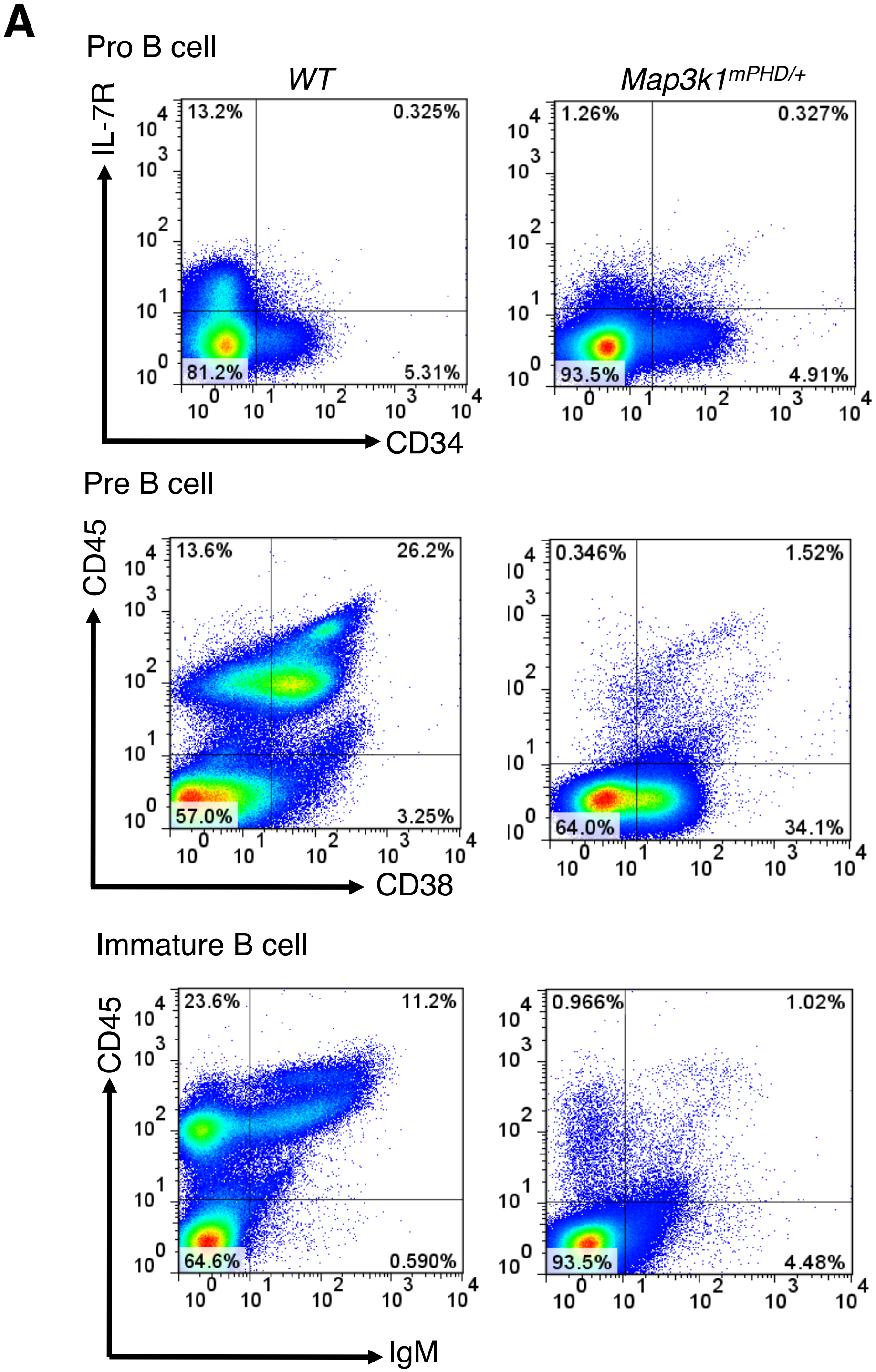
A
Pro B cell
WT
Map3k1mPHD/+
IL-7R
CD34
13.2%
0.325%
81.2%
5.31%
1.26%
0.327%
93.5%
4.91%
Pre B cell
CD45
CD38
13.6%
26.2%
57.0%
3.25%
0.346%
1.52%
64.0%
34.1%
Immature B cell
CD45
IgM
23.6%
11.2%
64.6%
0.590%
0.966%
1.02%
93.5%
4.48%

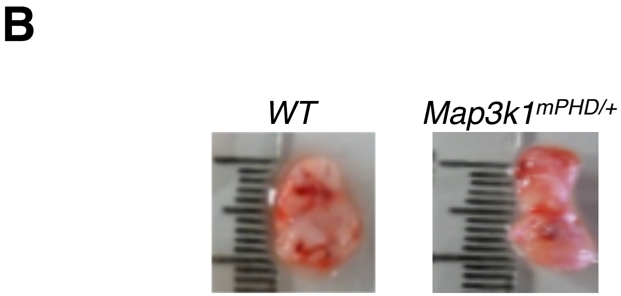
B
WT
Map3k1mPHD/+

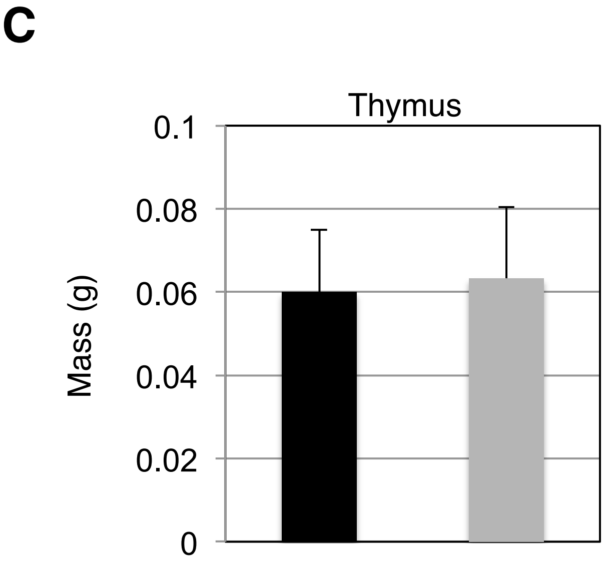
C
Thymus
Mass (g)
0.1
0.08
0.06
0.04
0.02
0
